# The burden of treatment in people living with type 2 diabetes: A qualitative study of patients and their primary care clinicians

**DOI:** 10.1371/journal.pone.0241485

**Published:** 2020-10-30

**Authors:** Pilar Espinoza, Camila A. Varela, Ivonne E. Vargas, Galo Ortega, Paulo A. Silva, Kasey B. Boehmer, Victor M. Montori

**Affiliations:** 1 School of Nursing, Pontificia Universidad Católica de Chile, Santiago, Chile; 2 Mental Community Health Center Pedro Aguirre Cerda, Santiago, Chile; 3 Andes Family Health Center, Santiago, Chile; 4 School of Nutrition, Universidad San Sebastian, Santiago, Chile; 5 Knowledge and Evaluation Research Unit, Division of Endocrinology, Diabetes, Metabolism and Nutrition, Mayo Clinic, Rochester, New York, United States of America; Queen's University Belfast, UNITED KINGDOM

## Abstract

**Background:**

The burden of treatment can overwhelm people living with type 2 diabetes and lead to poor treatment fidelity and outcomes. Chronic care programs must consider and mitigate the burden of treatment while supporting patients in achieving their goals.

**Objective:**

To explore what patients with type 2 diabetes and their health providers consider are the workload and the resources they must mobilize, i.e., their capacity, to shoulder it.

**Methods:**

We conducted focus groups comprised of 30 patients and 32 clinicians from three community health centers in Chile implementing the Chronic Care Model to reduce cardiovascular risk in patients with type 2 diabetes. Transcripts were analyzed using thematic content analysis techniques illuminated by the Minimally Disruptive Medicine framework.

**Findings:**

Gaining access to and working with their clinicians, implementing complex medication regimens, and changing lifestyles burdened patients. To deal with the distress of the diagnosis, difficulties achieving disease control, and fear of complications, patients drew capacity from their family (mostly men), social environment (mostly women), lay expertise, and spirituality. Clinicians found that administrative tasks, limited formulary, and protocol rigidity hindered their ability to modify care plans to reduce patient workload and support their capacity.

**Conclusions:**

Chronic primary care programs burden patients living with type 2 diabetes while hindering clinicians’ ability to reduce treatment workloads or support patient capacity. A collaborative approach toward Minimally Disruptive Medicine may result in treatments that fit the lives and loves of patients and improve outcomes.

## Introduction

Type 2 diabetes is a prevalent condition globally and in Chile, affecting 12% of adults [1] and costing US$2 billion/year or 0.9% of that country’s gross domestic product [2]. In response, the Chilean Ministry of Health has implemented a cardiovascular care program using the Chronic Care Model (CCM) [3].

The CCM, first proposed by Wagner in 1996 [4] sought to improve the primary care of patients with chronic conditions, by ensuring that proactive and well-supported teams worked together with activated patients to achieve disease targets following evidence-based protocols of care. Chronic care programs, to be successful, should consider the work patients must do to access and use healthcare services and to enact self-care tasks, as the feasibility of their processes and their outcomes depend on this work. This workload includes activities such as arranging and attending in-person visits with the care team, obtaining and organizing the administration of medications, making dietary changes, and performing regular physical activity [5, 6].

To shoulder the workload, patients must mobilize their capacity understood as the collection of abilities and resources to enact care. This capacity also encompasses the contributions of the patient’s family and community [6, 7]. Because this capacity is also used to face other demands of life, self-care competes with life demands for the same resources, abilities and social support [8]. A systematic review and meta-synthesis inquiring about the ability of the CCM to meet the needs of patients with multimorbidity, however, reported that its implementations offer minimal support for patients in reformulating their lives, implementing treatment work, or in improving their functional status and quality of life [9]. In other words, CCM implementations are often blind to the burden of treatment.

The burden of treatment refers to “the personal workload of healthcare, including treatment and self-management of chronic health conditions, and the impact of this workload on patient functioning and well-being.” [10]. As patients become overwhelmed under the burden of treatment, they may not be able to implement care with adequate fidelity, complicating their care, wellbeing, and the relationship with their clinicians [11]. To deal with this complication, Minimally Disruptive Medicine (MDM) has been proposed as a model of care that pursues patient-centered care goals while minimizing the burden of treatment [5, 12]. MDM is particularly well suited for the care of patients with chronic multimorbidity and psychosocial complexity.

We sought to conduct a qualitative analysis of workload and capacity in patients with Type 2 diabetes receiving care in the community health care system in Chile. This study seeks to uncover their experience, along with clinicians’ perspectives, of burden of treatment and of the capacity they have to shoulder it. This knowledge should support the implementation of MDM in the care of patients living with Type 2 diabetes and its treatment.

## Methods

We chose a qualitative descriptive approach and adopted a subtle realist stance to explore and describe thematically treatment burden, allowing an understanding of the lived experience, free of presuppositions and intuitive of the reality [13].

The study, conducted between April and December of 2017, was carried out in three Family Health Care Centers belonging to the public health system in Santiago, Chile. While convenient, these Centers are also representative of centers within the healthcare system, in that they share similar number of patients with Type 2 diabetes, socioeconomic characteristics, quality of diabetes control, and staffing. In Chile, patients with type 2 diabetes are admitted to the cardiovascular program. In this program, physicians conduct a 30-minute consultation every 6 months, while patients visit with a diabetes nurse educator and with a dietitian once a year. To respond to medical concerns, patients can receive additional 15-minute appointments with physicians who see approximately 10 patients per day.

After socialization with the health center's authorities, a purposive sample of patients with Type 2 diabetes enrolled in the cardiovascular health program were approached in the waiting room. After explaining the study objectives, we invited them to participate in a focus group that same day while sharing a healthy breakfast with researchers. The majority of patients and clinicians accepted the invitation. The ones who declined cited lack of time as their reason. We hosted one patient focus group at each participating health center. A purposive sample of clinicians at each center were also invited to participate in a separate and local focus group. All participants completed written informed consent procedures. The Metropolitan South-Orient Health Service Ethics-Scientific Committee, the Metropolitan Occident Health Service of the Ethics-Scientific Committee and the Pontific Catholic University of Chile Ethics Committee approved all study procedures.

Participants completed a sociodemographic survey at the beginning of their group session (~5 minutes). These sessions followed a script “S1 Text” based on MDM and on the items in the Burden of Treatment Questionnaire [14]. Health professionals reviewed the script for content validity and language suitability [15, 16].

The core research team consisted of a Registered Nurse with a PhD (PE), a Nursing Sciences master's student with experience in community health (CV), a Professor at the School of Nursing with a master's degree in Bioethical Sciences (IV), a Family Physician (GO), and a Registered Dietitian pursuing a PhD in Nutrition Sciences (PS). Each member of the research team has prior qualitative research experience. No member of the research team worked at the community health centers in the study. P.E. moderated each focus group session (~50 minutes) while C.V. and I.V., also members of the research team, took field notes. The sessions were audio-recorded and transcribed verbatim, using the NVivo qualitative data analysis software (QSR International Pty Ltd. Version 12, 2018). NVivo was also used to analyze the latent content of the speech with depth and abstraction allowing interpretation of underlying meaning [16].

The thematic content analysis uses both an inductive and a deductive approach, providing a rich description of the overall situation and also a more detailed analysis of essential aspects of the narratives. In this analysis, context helps to make sense of the manifest and latent aspects of the data, while retaining the meaning of the experience and emotions of participants [13, 16].

This process achieved identification, indexing, and retrieval of relevant categories and organized the text around the broader themes of workload and capacity, and finished by data saturation and triangulating the results [12]. Two researchers, P.E. and C.V., working independently, conducted the content classification process of the all transcripts, with a third researcher, IV and PS., intervening when doubts or disagreements in coding emerged. Thus, the conduct of the focus groups and their analyses sought to optimize credibility, confirmability and transferability, key methodological requirements for qualitative rigor [16].

## Results

### Patient focus groups

All patient participants had Type 2 diabetes and at least one other chronic condition, were aged 35–75 years, and participated in a focus group at one of the three participating Health Centers (HC): HC1 (n = 12), HC2 (n = 8), and HC3 (n = 10 joined by 2 relatives).

### Clinician focus groups

Family physicians and specialists, registered nurses, nutritionists and a pharmacist took part in clinician focus groups at each site: HC1 (n = 12), HC2 (n = 8), and HC3 (n = 12).

### Themes

The analyses of the narratives “S2 Text” uncovered six themes related to treatment workload and five themes related to patient capacity.

#### Workload- barriers on the health system

Patients and clinicians agreed on the multiple challenges of accessing health care, including overcoming numerous administrative hurdles to access initial and follow-up consultations. Clinicians reported feeling constrained by rigid protocols, limited treatment options, and limited time in consultation.

#### Workload- relationship with the healthcare team

Patients reported brief encounters and communication barriers during the consultation in which clinicians spent limited time supporting and educating, emphasizing instead the review of test results and the need for adherence to medications. Clinicians agreed with this characterization of their practice.

#### Workload-patient education

Patients felt uninformed, for example about drug adverse effects and interactions, turning to lay people for information. Clinicians reported feeling unable to offer adequate information and support to their patients or to adjust the protocols to their particular situations

#### Workload- healthy diet

Adherence to diet recommendations was particularly difficult in social gatherings, in which patients preferred to deviate than have their restrictions interfere with pleasure or interaction. Clinicians are aware of this reality but felt unable to change it because is part of the cultural background, to associate social get-togethers to food consumption.

#### Workload-drug treatment

Patients described the difficulties of taking with too many drugs and implementing complex regimens and complicated routes of administration. Clinicians recognized the limited treatment options and also acknowledged the lack of information available to the patient.

#### Workload- physical activity

Patients recounted the difficulties to follow clinicians’ rigid physical activity advice, which they considered blind to the realities of these patients’ lives, including physical limitations and unsafe neighborhoods. Clinicians felt that patients don’t seem to understand the importance of exercise, or patients are unable to see the difference between physical activity and house chores or workload. Direct quotations from participants are used to illustrate each theme related to treatment workload in “Table 1”.

**Table 1:** The work of patients living with type 2 diabetes.

	Patients	Clinicians
**Barriers on the health system**	Of course, it's not easy, you have to get up very early to get an appointment with the physician. (HC1P5).	I do not know if it is the optimal program [cardiovascular]. A well-controlled patient is seen in three months, and after that, a lot of time passes! (HC1HP2)
	They say, "it's going to be a while because the agenda for the health professionals is replete." (HC2P5)	
		On the consultation, you have to write everything down. (documentation for the health center] (HC2HP4)
	It was a year without seen a physician, or the nutritionist, they gave me the medicines every month. . . but I had no consultation. (HC2P4)	
		Our system is too rigid, clinical judgment should be more important than a standard protocol! (HC3HP9)
		One must review test results, complications, drugs, lifestyle changes in 30 minutes. I cannot do magic! (HC3HP6)
**Relationship with the healthcare team**	They [physicians] ask you 'what are you taking?' and when you tell them, they give you the same, and then they call the next patient [not more communication or relationship] (HC1P1)	Health professionals don’t speak the same language and sometimes they contradict each other confusing the patient. (HC2HP1)
	Suddenly we can, not with scientific bases, but with our instinct, discuss with the doctor, before that wasn’t allowed (HC3P5) because the physician was always right! (HC2P3)	Here, the physician is God, whatever they say the patients do. (HC2HP4)
		Patients could skip appointments with the nutritionist or the nurse, but not with the physician, because they give you the medications. (HC3HP10)
	A physician could give you confidence with his kindness and disposition. (HC3P5).	
**Patient education**	They [health team] didn’t explain to me [that I had diabetes]. . . so I didn’t know how to care for myself. I had no idea! (HC1P3)	The time for patient education is very little, that is why we have a growing number of decompensated diabetics. (HC2HP6)
	The greatest difficulty in dealing with the disease is not knowing the symptoms and what to do about them (HC1P2)	There isn’t formal education. I try to explain everything to them, and they seem interested, but it is difficult for them to understand. (HC1HP3)
	[Where is the insulin placed?] In this part [showing]? (HC2P2) No! it is not placed there! (HC2P3) The insulin is placed in the arms and the tummy, here. (HC3P6). There is fat diabetes and skinny diabetes, she has the skinny because she was chubby, and she became thin [laughs] I have fat diabetes because I'm still the same. (HC1P4) [patients educating each other during the focus groups]	
		I believe that both the patient and their family should be educated. Patients who have an average level of education and support from their family have better results controlling the disease. (HC3HP8)
**Healthy Diet**	You can take care of yourself, take the medicines, but the food, no! It is too delicious [everybody laughs and nods]. You cannot leave the food that you love. (HC1P6)	The cultural reality, where bread, dough, flour, is very important. (HC1HP5)
		They spend on soda drinks more than 500 dollars a month! (HC3HP1)
	I still eat everything, beef, pork, sausages. (HC3P4)	
		It makes the patient happy to eat something rich with the family, to take that away that, is like taking away their world! (HC2HP7)
	One cannot go to parties, because we have to only talk now, not eat or dance, nothing more. (HC2P3)	
		As long as they feel fine [physically], they don’t change their habits. (HC1HP4)
**Drug treatment**	Before they use to give the patient everything on the physician 's offices [they mention the glucose self-monitoring strips]. Now you have to buy the strips and they last nothing. (HC2P2)	Patients complain a lot about metformin, it is too big, they cannot swallow it or produces gastric intolerances, but there aren’t options! (HC1HP4)
	Metformin is a big tablet and you have to swallow it anyway. (HC1P7).	The way they manage their medicines depends on their health education, otherwise it could be chaos. (HC2HP1)
	Those pills [metformin] are huge. I feel like chocking, every time I try to swallow them [showing with her finger how large the pill is] (HC2P4).	Patients get bored and confused on the right doses, they confuse the colors, they have a hard time reading the labels. (HC3HP7)
	When you start [medicines], you get like weird feeling, but when you stop taking them it also feels strange, like the body asks for the medicine. (HC3P10)	
**Physical activity**	One is told that walking in the house isn’t physical work, you have to walk outside. (HC1P7)	“Doctor, I'm always moving around the house. . .” Every patient says that [Everyone agrees]. (HC1HP1) or they said I walk half an hour to go to take the bus. (HC3HP2).
	I used to walk around before, now the neighborhood is too dangerous, people drinking on the streets or smoking pot, even selling marijuana. (HC2P5)	
		Some people are not interested in exercising, others complain about knee or hip osteoarthritis. (HC2HP5)
	[They insist I walk regularly but] I don't feel half of my feet, even if the shoe is good, I do not feel it. (HC3P4)	Some patients attend exercise class at the health center, and it works super well, but the number of members is limited. (HC1HP6)

HC: family health center; HP: Health Provider; P: Patient. e.g., (HC1P5) = Patient 5 at the first family health center.

#### Capacity–biography

Patients and clinicians agreed on the challenges that patients face when dealing with the initial diagnosis of Type 2 diabetes. Patients remember feeling unable to cope and overwhelmed by grief and fear, especially for disease complications like the amputation of a leg, comparing the disease to a death sentence. Clinicians recognized that the diagnosis of diabetes along with insufficient education soon after diagnosis brought about a sense of crisis into patients’ lives. Myths and misconceptions often occupied the place that evidence-based information should have filled.

#### Capacity–resources

Patients reported drawing from two sources that clinicians did not consider. Patients reported using diets and herbs as part of their treatment, assuming they are efficacious, and sharing their experience with other patients. Also, they drew from their religious practices to gain comfort, community, and strength to deal with the difficulties that the illness brings to their lives.

#### Capacity–environment

Patients report, and clinicians recognized, that the work environment could be hostile to patients with Type 2 diabetes. Patients reported fear of being considered a burden by their employer and opting instead to hide their diagnosis. This made it difficult for patients to reconcile treatment demands with work responsibilities, prioritizing the latter for their families’ sake.

#### Capacity–social support

Patients and clinicians recognized the importance of family support. Family support differed, however, depending on the gendered role of the patient. For example, family members (wife, daughters) supported fathers while sharing the responsibility with them of changing habits. Husbands recognized the important disease management role their spouses played, but also assumed that their wives were simply fulfilling their duty to do so. Mothers were expected to take care of themselves while continuing to routinely support their families.

#### Capacity–realization of necessary work

Participants recognized the value that mastery and success has in empowering patients to manage their condition. Patients, particularly women, were proud of their ability to enact self-care, even when others failed to understand their struggle or to offer support. Conversely, disempowered patients frustrated clinicians, who felt they could only support them with information.

Direct quotations from participants are used to illustrate each theme related to patient capacity in “Table 2”.

**Table 2:** Capacity in patients with type 2 diabetes.

	Patients	Clinicians
**Biography**	The physician told me, “Type 2 diabetes was like cancer, a silent cancer” [all claim to have heard that phrase]. (HC2P1)	Patient first reject the diagnosis, they have an emotional crisis, denial, they consider it a death sentence, the evolution depends on the family support. (HC2HP3)
	It is cheaper to amputate the leg than treat and cure it. (HC3P4)	
		Patients knows and sees other patients and the consequences of the disease and it scares them. (HC1HP1)
	I cried a lot, because I have relatives who have had their legs amputated because of wounds that do not heal. (HC2P3)	
		They worry about never be able to eat a sweet again. (HC2HP5)
	The other thing is the leather shoes, that is very expensive. (HC1P7)	
		Patients have a lot of beliefs in myths about diabetes. (HC3HP3)
**Resources**	I used to have to inject myself with insulin three times a day, but I received from a friend a herb called Alcampuri and now I'm controlling myself with that. I used to have glucoses of 400, 500, and now I have them between 85 and 110 no more. (HC3P9)	
	In my life, I used to be a heavy drinker, but one day I met the Lord, maybe it's not the time to talk about this? [other people: "It's fine, it's good "] And God told me that I was going to be healed of my diabetes. I still take good care of myself, but I am more flexible!! [laughs] (HC2P7)	
**Environment (work)**	I knew of a person who was fired for saying he had Type 2 diabetes. (HC1P2)	For the patients that work, coming to the health centers for consultation is very difficult [everyone agrees]. (HC1HP5)
	People at work consider a person with diabetes a high risk for the job. (HC1P5)	
	I have to take all my medicines together in the morning, because when you start working you cannot leave to take them. (HC2P1)	Patients might get fired because of the disease. (HC3HP4).
		Patients often work very far from home, having to endure a long bus journey, without much time for breakfast or to take their medicines! It’s super complicated! (HC2HP2)
	I believe organizations should give workers with Type 2 diabetes permission to go to the physician. (HC3P4)	
**Social (family) support**	[My wife] is better than a nurse, she cares about me, she could be very demanding, making sure I am following the health team indications. (HC3P3)	There are patients who say "in my house I swim against the current and I am very tempted specially by food. (HC2HP4)
	My children worried about all the medicines I need to take. (HC2P1)	There is a lot of machismo, when the man gets sick there is more support, the diet of the whole family is modified”. (HC1HP6)
	My family worries, “did you inject the insulin? Did you take the pills? Take care, sit for a little while.” (HC1P7)	
		Men by culture don’t take care of their disease as women do, they expect their family to support them. (HC3HP7)
		[Management of Type 2 diabetes] depends on the family, mostly housewives or grandmothers. Their children work and support them, while they take care of their own children. Sometimes there is no support at all. (HC1HP5)
**Realization of necessary work**	I lost my vision. . . but they gave me a dog and we both have the same motivation to keep going! Diabetes is something that happens to me and I have to face it. (HC3P7)	They throw the ball [responsibility] at you, because they will not make their own decisions. They aren’t empowered to do their self-care. . . when we talk to the patients, they begin to understand. . . but they have many wrong ideas about how to manage they self-care. (HC2HP6)
	In my house they do not believe that I have Type 2 diabetes because I do everything, I take care of six people, but above all I have to take care of my son who is sick. (HC2P3)	
	I don’t go out much. When I go to my son to spend a night, I have to take a little bag with all my medicines. . .You have to walk with everything [laughs] like a living pharmacy. (HC2P6)	
	For me it has not been so difficult because my husband and I eat the same food, with little sugar and salt. This routine helped a lot when he had a heart attack, because he had already gotten used to eating healthy. (HC1P2)	

HC: family health center; HP: Health Provider; P: Patient. e.g., (HC1P5) = Patient 5 at the first family health center.

## Discussion

### Our findings

Patients with Type 2 diabetes in this sample found seeking access, attending numerous appointments, and working with their clinician as contributors to their patient workload. Patients struggled to implement complex medication programs without adequate knowledge of their indications, interactions and adverse effects. Patients also struggled to become more physically active and to change eating habits, fearing isolation and experiencing frustration in a culture in which food is a source of pleasure and socialization. They also hid their disease at work to avoid stigma. To deal with the distress of the diagnosis, difficulties achieving disease control, and fear of complications, patients sought capacity from their family (mostly men from their spouses), social environment (mostly women from their friends), traditional medicines and foods, faith and religion.

Clinicians found that the completion of administrative tasks, limited range of therapeutic alternatives, and protocol inflexibility–for both cardiovascular and CCM protocols—impeded their ability to assess and respond to their patients’ burden of treatment by supporting them and modifying care plans. Although they reported recognizing their patient’s workload, clinicians expressed impotence to change the systems of care in which they worked.

### Comparison with other studies

Our findings are mostly consistent with the global literature. While studies have linked organization of care factors to poor treatment fidelity [17], most implementations of the CCM do not account for burden of treatment and capacity [9]. In particular, our findings within this implementation of the CCM studied seem incompatible with one of its core feature, namely, productive and participative interactions between patient and clinician to promote patient self-management and improve adherence to treatment [18, 19]. The rigidity of the protocols fails to account for the importance of considering patient characteristics–such as patient’s perceptions and beliefs [17] when creating a health care plan [20] that makes practical sense in the lives of patients [21]. Patients have to do additional work to negotiate with their clinician, with their jobs, and with friends and families, Women face additional challenges as our study and others indicate that women perform more self-care activities in their daily lives [22], supported by their community and take responsibility for the self-care of husbands and family members [23, 24].

Our findings related to patient capacity are also consistent with the review by [8] which identified Biography, Resources, Environment, Work (Self-efficacy), and Social support (BREWS) as the sources of patient capacity to address treatment workload.

### Strength and limitations

Our study is cross-sectional and of a nonrepresentative sample. Interviews could be limited by recall and social desirability biases. Furthermore, some issues, e.g., low literacy, financial hardships, may not emerge because of the group setting used. Indeed, low literacy was brought up only by the clinicians. Yet, this is the first evaluation of patient workload and capacity in Chile, if not in Latin America, with the joint consideration of patient and clinician views, a novel contribution to this literature globally.

### Implications

Our study has implication for the application of the CCM to the care of patients with multiple chronic conditions in Chile. Indeed, the rigid application of protocols in a manner unaware of or unable to respond well to patient workload and capacity issues fails the patient, frustrates clinicians, and wastes scarce healthcare system resources.

Our findings support the exploration of MDM as an alternative model for the care of patients with Type 2 diabetes and other chronic conditions. Its implementation may require substantial changes to the way healthcare is staffed, information resources organized, professionals trained, protocols designed, and process outcomes monitored [6]. For example, MDM needs clinicians to be aware of the burden of treatment their patients experience. Several tools, in need of translation and adaptation to the Chilean setting, are available to assess and monitor treatment burden along with other measures of care and disease control [25]. Treatments must be made available to allow for more flexible and feasible regimens that could be implemented without overwhelming patients and families. Shared decision making [26] and conversations supported by the instrument for patient capacity assessment [25, 27], properly adapted, may facilitate this process. Information systems must be attuned to signals that care may have become overwhelming, e.g., poor fidelity to treatment programs, missed appointments, poor disease control. And patients and their families should receive support that goes beyond disease education (using low-literacy material and peer support [28] to include self-management training, social support [29] and capacity coaching [30]. What may arise is care capable of advancing patient health goals with the smallest treatment burden possible [12, 21].

Patients with Type 2 diabetes experience substantial workload, in part imposed by the way healthcare is organized within the cardiovascular program in Chile. Patients deal with this without substantial contribution from their clinicians, who feel unable to respond by modifying disease-oriented protocols of care structured within the CCM.

The way forward, minimally disruptive medicine, would require the incorporation of the assessment of burden of treatment in routine care and in patient-clinician conversations, the incorporation of patient workload as a modifier of clinical protocols, and the provision of intervention to improve patient capacity through individual and social support to reduce burden of treatment. In turn, a collaborative approach may result in treatment programs that better fit the lives and loves of patients with Type 2 diabetes, which in turn will reduce burden of treatment and may improve adherence and outcomes.

## Supporting information

S1 Text Scrip for focus groups. (DOCX)

S2 Text Narratives from focus groups. (DOCX)
