# Supplementary material for: The burden of treatment in people living with type 2 diabetes: A qualitative study of patients and their primary care clinicians
Source: PLoS One. 2020 Oct 30;15(10):e0241485. doi: 10.1371/journal.pone.0241485 (PMC7598471; doi:10.1371/journal.pone.0241485)
Supplement: S1 Text — (DOCX) [file pone.0241485.s001.docx]

**S1 text. Scrip for focus groups:**

**SCRIP FOR FOCUS GROUPS WITH TYPE 2 DIABETES PATIENTS**

1.-¿What are your chronic diseases?

2.-¿What are the healthcare indications or drug treatment that your chronic illness requires?

3.-¿Could you please tell us about what do you do for selfcare and the amount of time and effort this requires?

4.-¿How does your daily life been affected by keeping up with indications or treatment given by the health team?

5.- ¿What aspects of your treatment are complicated, difficult or take too long, and cause discomfort in your daily life?

Support Questions:

A.-Drug treatment:

1.-Approximately how many tablets, capsules, sachets, injections or inhalations do you take per day? And more or less how many times a day? How long does it take you to organize your treatment?

2.-What things do you do so you don’t forget to take your medications when appropriate?

3.-What do you do when you need more medications?

B.-Exams and control

1.-How much time do you spend doing tests such as measuring your blood sugar or taking your blood pressure?

C.- Consultations with the health team

How many different doctors do you see on a regular basis?

How many times have you been to an appointment with a doctor or other health professional in the last month? How do you organize health visits? (time spent, difficulties in making an appointment or transportation)

D.- Food

How does it affect your life, having to follow a specific diet, stop eating certain foods and / or alcohol, stop smoking?

E.- Physical Activity

How does it affect your life following the recommendations of doing sports or physical exercises?

(for example: walk regularly ...)

F.-Social, Personal, Labor

What difficulties do you encounter in your daily life when integrating health care into family, social or work life? (for example, having the impression of being a burden to others, being ashamed to take your medication in public, making an effort to hide your illness, reconciling treatment with cultures and beliefs ...)

G.- Health System.

What difficulties would be related to the health system (financial support, access to medical care at the center and home, the operation of the hospital, health policies ...) Tell us about the impact of these annoyances on your daily life

**SCRIPT FOR FOCUS GROUPS WITH CLINICIANS**

1.-¿What are the chronic diseases of your patients?

2.-¿What are the healthcare indications or drug treatment that their chronic illness requires?

3.-¿Could you please tell us what do you think your patients should do to take care of themselves and how much time and effort they need for this?

4.-¿How do you perceive the activities required for your patients to follow the indications or treatment given by the health team? And, how do you believe it affect your patient’s daily life?

5.-¿What aspects of the treatment of your patients do you consider to be complicated and difficult for them to adhere, or can take too long to do it, and cause discomfort in their daily life?

Support Questions:

A.-Drug treatment:

1.-Approximately how many tablets, capsules, sachets, injections or inhalations does your patient take per day? And more or less how many times a day? How long does it take for them to organize your treatment?

2.-What things does your patient do so they don’t forget to take their medications when appropriate?

3.-What do they do when you need more medications?

B.-Exams and control

1.-How much time does your patient spend doing tests such as measuring their blood sugar or taking their blood pressure?

C.- Consultations with the health team

How many different doctors does your patient sees on a regular basis?

How many times do you think, have your patients been to an appointment with a doctor or other health professional in the last month? How does your patient organize their health visits? (time spent, difficulties in making an appointment or transportation)

D.- Food

How does it affect your patient’s life, having to follow a specific diet, stop eating certain foods and / or alcohol, stop smoking?

E.- Physical Activity

How does it affect your patient’s life following the recommendations of doing sports or physical exercises?

(for example: walk regularly ...)

F.-Social, Personal, Labor

What difficulties does your patient encounter in their daily life when integrating health care into family, social or work life? (for example, having the impression of being a burden to others, being ashamed to take their medication in public, making an effort to hide their illness, reconciling treatment with cultures and beliefs ...)

G.- Health System.

What difficulties would be related to the health system (financial support, access to medical care at the center and home, the operation of the hospital, health policies ...) Tell us about the impact of these annoyances on your patients’ daily life
