# Supplementary material for: The burden of treatment in people living with type 2 diabetes: A qualitative study of patients and their primary care clinicians
Source: PLoS One. 2020 Oct 30;15(10):e0241485. doi: 10.1371/journal.pone.0241485 (PMC7598471; doi:10.1371/journal.pone.0241485)
Supplement: S2 Text — (DOCX) [file pone.0241485.s002.docx]

**S2 text. Narratives from focus groups:**

**HEALTH CENTER 1- PATIENTS**

**Investigador (I):** Partir por agradecerles que quieran compartir su experiencia con nosotros. Como yo les decía para nosotros es súper importante tratar de comprender un poco más que significan las enfermedades crónicas en el día a día de las personas. Cuando a uno le dicen ‘sabe que usted se ha sentido mal porque les hicimos exámenes y tiene diabetes’, y el médico te dice ‘vamos a tener que hacer estos cambios en la vida de ustedes, en el estilo de vida’, queremos saber cómo enfrentan esa realidad, qué cosas son las más complejas, que pasa con la vida de ustedes cuando les hacen el diagnóstico.

**Participante (P):** primero que nada, cuando nos hacen el diagnóstico que nos encuentran el azúcar muy alto y todas esas cosas, uno primero se siente mal y después cuando ya pasamos al nivel de hacer los regímenes, todas esas cosas, uno primero piensa que es caro para uno, y muchas veces, lo que me paso a mí el año pasado que no trabaje durante todo el año, se me hace difícil, como para yo comprarla… me las invento, como corresponde.

**I:** Claro, las indicaciones de alimento

**P:** Claro, eso. Y ahí al no llevar el régimen se complica más la enfermedad

**I**: ¿y los medicamentos se los dan acá verdad?

los dan acá

**P:** No, cuesta. Por ser los primeros días tiriton…

**I**: ¿y eso lo esperaba?

**P:** No… Después cuando uno los deja de tomar también se siente raro, se siente raro también, empieza con los mismos síntomas, pero al revés.

**P**: Lo mismo, pero al revés, el cuerpo le pide el remedio… Eso.

**I**: ¿Cómo fue la experiencia?

**P:** Yo puedo contar mi experiencia de 15 años más o menos que me diagnosticaron diabetes.

**P:** …eeh tengo Mellitus II también, adquirida como a los…40 más o menos me lo…eeh…la mayor dificultad de la enfermedad es no saber los síntomas que uno tiene dependiendo de……porque muchas veces a uno le sube la azúcar y no tiene cómo saber …si alguien que no es preparado, que no sepa o que no estudies que … por qué tiene la enfermedad, qué les pasó, primero que nada no sabe qué es lo que le pasa, si sube el azúcar está la vista nubló… nublada, eeh muchas ganas de tomar agua…y a veces es el contrario, pero uno no sabe cuáles son los efectos que va a tener con eso, también uno tiene que estar esperando las veces que viene al consultorio para saber cuánto de azúcar uno tiene.

**P**: es que eso depende de cómo… cómo… no… de como esté el consultorio con horas. No depende de uno.

**P**: o sea, pero de todas maneras uno como paciente crónico tiene alguna prioridad

**I**: ya…

**P:** …o sea puede venir y decir y le buscarán ajuste de hora, o la mandan a tomar la glicemia de todas maneras como para saber…o lo de la hipertensión para ver como está. Eso por lo menos así, pero no es todos los meses para venir uno a ver cómo está todos los meses.

**P:** exacto, no es una… un, una metodología total

**P:** continuada (*complementa a respuesta anterior*)

**I:** Ya…

**P:** o sea que fuera de todos los meses que uno viniera…

**P:** no es un control permanente (*se sobrepone a respuesta*)

**P:** …usted viene cada dos meses

**P:** cada tres meses…le están dando la hora

**P:** cada tres meses, a menos que usted esté mal

**P:** cuando uno está bien cada tres meses, cuando está con la azúcar alterada asiste más…

**P:** Eso hace que uno muchas veces esté descompensado, en el sentido de que no tiene ganas de hacer nada, de repente está con la vista mala, que generalmente eso pasa con la diabetes.

**I:** si

**P:** que la vista molesta esta de una manera diferente

**P:** (*se generan conversaciones paralelas*) Cuanto se llama…la diabetes les entra a los ojos rojos porque yo tuve control en el San Rafael que me mandaron de aquí…

**I:** ya…

**P:** …aquí ando trayendo la receta, me encontraron este ojo que tengo diabetes.

**P:** Eso se llama fondo de ojo

**P:** El fondo de ojo, sí

**P:** ahí a uno le hacen. A todos nos mandan hacer una vez los exámenes que tienen que hacer, pero eso no es como que lo hagan todos los meses.

**I:** Perdón no la alcancé a escuchar

**P:** El control es por ejemplo si uno está bien la citan cada tres meses...

**I:** ya

**P:** …a control, le hacen los exámenes, el perfil lipídico

**I:** Si

**P:** y todo pa que le den el [¿?], eeh no es un control que sea seguido. Al principio cuando a uno le detectan el...la diabetes es más seguido, pero cuando uno ya se estabiliza ahí la citan más a lo lejos.

**I:**¿y en el día a día?

**P:** claro porque uno de repente un mareo, un dolor a las piernas, dolor a los brazos, circulaciones, dolor de cabeza…

**I:** Ya

**P:** y uno no sabe.

**P:** Claro, no es fácil, porque a veces no hay hora, o hay que levantarse muy temprano para que le den una hora y dependiendo de cómo esté le dan la hora, si no dan hora para otro día.

**P:** pero cuando usted dice que es un paciente crónico aquí generalmente le dan la hora

**P:** no sé, por lo menos a mí no me ha pasado

**P:** yo sí, por eso

**P:** a mí tampoco

**P:** en mi caso nunca he tenido problemas de ninguna especie en cuanto a las horas. Todo lo contrario, porque vengo al dentista me encuentran la glicemia alta, me mandan a colocarme suero y a controlarme al tiro.

**P:** lo pasa que la diabetes es pasiva uno no se da cuenta de… yo por ejemplo tengo… póngale cuatrocientos y tanto

**I:** Ya

**P:** y yo me siento como avión, ando (sonido ascendente), en cambio me bajan la glicemia y ando pa allá y ando pal otro lado.

**I:** o sea ……..

**P:** claro depende de…cada cuerpo tiene un comportamiento distinto, una reacción distinta.

**P:** Yo he llegado tal… ¿tal que le llaman? ya no tiene numerito la máquina.

**P:** así he llegado aquí, me han puesto suero…

**P:** uno no se da ni cuenta…

**I:** ¿Cómo es llevar esta enfermedad?

**P:** la dieta (*risas*)

**I:** hablemos de la dieta…

**P:** no, no es la dieta

**P:** ¡Sí! es la dieta (*se genera discusión entre los participantes*)

**I:** ¿y experiencia de cada uno?

**P:** El desorden que uno hace en la alimentación que no come a sus horas, cosas…

**P:** que come azúcar…

**P:** si eso puede ser, pero no en todos los casos. A mí me sucede.

**P:** a mí me tiende a bajar el azúcar, a mí casi que me dio un coma.

**P:** Si

**I:**¿cómo se organizan?

**P:** Si / Yo creo que sí / Tener harta plata

**P:** tener harta harta pa poder comprar los…

**P:** y los horarios

**P:** primero que nada, porque tiene que comer más horas, más veces en el día, antes comía tomaba desayuno almorzaba y…

**P:** una colación

**P:** …y la once

**P:** eso era antes. Y el plato contundente, y comer cualquier cosa o comer al caminar. Eso un diabético no lo puede hacer. Por qué, porque tiene que tomar desayuno a un horario, almorzar a un horario, después comer una colación entre el desayuno y el almuerzo…

**P:** …no una colación fantástica pero chiquitita…

**P:** …después entre almuerzo y once también una colación, y después la cena que es la última a las 8

**I:** ¿y Cuando uno vuelve a la casa?

**P:** Sí, porque casi la mayoría, por ejemplo, del sector donde nosotros vivimos nosotros trabajamos casi la mayoría. La Sra. Raquel trabaja en la feria, yo trabajo en la feria, hay muchos otros que trabajan en la feria, y nosotros en la feria no tenimos…eehh…no podemos llevar un control, ¿por qué? Porque no tenimos un horario. La fuerza que nosotros hacimos es diferente a la que hacen otras personas.

**I:** ya….

**P:** más energía, y eso es lo que la diabetes le quita: la energía. Porque le duelen los músculos como decía el caballero, no tiene ganas de levantarse, no tiene ganas de… que le duelen los tobillos, que le duelen las rodillas, que si das un mal paso o te das vuelta muy brusca te vas al suelo…

**P:** pero eso generalmente es por azúcar alta

**P:** no puede correr, no puede hacer nada uno, no puede correr

**P:** no necesariamente

**I:** ¿no?

**P:** yo ya no puedo ni correr ya

**I:** ¿y qué hace uno? …

**P:** comer… comer

**P:** comer algo, y tener…eehh estar, quedarse quieto, respirar profundo y descansar

**I:** ¿y qué pasa en otro lugar?

**P:** desgraciadamente tenemos que hacerlo y tenemos que llegar…

**P:** hasta llegar a la casa (*sobrepone respuesta de P7*)

**P:** …yo por ejemplo yo aquí me he [*rajado*], porque he tenido problemas a los músculos entonces me tengo que hacer un examen que es súper caro y primero tengo que conseguirme la hora para el especialista que es un neurólogo que hace a los nervios…

**I:** ya

**P:** …y aquí no hay neurólogo, entonces hay que hacérselo particular

**P:** Si / si exacto.

**I:** ¿Y lo que tienen que comer?

**P:** esa es otra…

**I:** ¿hablemos de eso?

**P:** antes que pasemos a ese tema a mi me complica ahora porque yo todos los días a las cinco y media estoy en pie…

**P:** …estoy tomando a las cinco y media, un cuarto pa las seis estoy tomando desayuno, de ahí tengo que aguantarme, me salto la colación que piden, todas esas cosas, me las salto hasta la 1

**I:** ¿Por qué?

**P:** porque no dan permiso de comer ninguna cosa

**P:** claro, no hay permiso, hay que trabajar

**I:** ya

**P**: Después de la 1 hasta las 8 que llego a la casa, de la noche. lo mismo

**P:** si encuentro alguna cuestión donde comprar una sopaipilla, un pedazo de pan así…

**P:** …y lo que no tendría que comer… (*cabalga respuesta*)

**P:** …y lo que no tendría que comer justamente, entonces tengo que hacerlo porque el cuerpo me está pidiendo alimentos poh

**P:** frituras no son alimentos

**I:** ¿y en el trabajo uno puede avisar?

**P:** si dan po

**P:** no, pero en el trabajo mío…

**P:** una vez lo despidieron a el por decir que era diabético

**P:** dure tres días en el trabajo, porque yo era diabético

**P:** si lo que pasa que…

**P:** es una persona de alto riesgo

**P:** que a lo diabético no le dan trabajo porque puede darle… uno está trabajando bien, y le da cualquier desmayo…[*clotió*]…y por eso está estrictamente…

**P**: la diabetes no es una enfermedad invalidante, por lo tanto, es muy difícil de que el doctor te vaya a dar un certificado. Además, para hacer un trabajo como el que cumple el caballero, tiene que perder horario y muchas veces dependen de los horarios porque van de sección en sección

**I:** claro

**P:** entonces esos son los problemas que a él…

**P:** no, y muchas veces de pesados no más que son los jefes

**P:** qué es lo que pasa, que nadie conoce la enfermedad, nadie la ha vivido, y nadie la ha explicado. Porque yo misma cuando yo…a mí me diagnosticaron esto que fue por un eh…susto que me dio, y yo llegué aquí, me desmayé y ahí seguí con la historia, pero yo trabajaba y yo trabajaba en la cocina, pero yo me saltaba todas las comidas y mis comidas eran lo que lograba picar así cuando probaba algo.

**P:** el picoteo

**P:** en mi caso fue diferente, porque yo trabajé muchos años en laboratorio

**P:** de investigaciones tecnológicas, entonces nosotros teníamos un horario de llegada, y yo llegaba en las mañana nos daban permiso para tomar un té, café, y después yo entraba a una sala de… que era muy triste porque era todo esterilizado y ya no salía hasta las 11 más o menos y ya yo venía…mucha veces me fui al baño porque me sentía tan mal, y salía mojada en transpiración y todos me preguntaban que me pasaba, y a mí me daba cosa decir que yo era diabética, porque me iban a despedir porque el trabajo mío era complicado, y una enfermera que había ahí, que trabajó de enfermera me dijo ‘nunca hagas eso, te puede dar un coma diabético en el baño y nadie va a saber’. Y entonces yo estaba acostumbrada a comer en la mañana al desayuno, y después hasta la 1 y lo que le pasa al caballero, y después llegaba a la casa a las cinco, seis de la tarde y ahí comía de nuevo.

**P:** Yo creo que ese es el problema más grande que hay

**P:** y la desinformación.

**P:** señorita un día yo comiéndome por ser un día saqué un racimo de uva, lo compartí con un compañero de trabajo y me retaron

**I:** ¿Por qué?

**P:** porque yo les dije es mi colación y mi cuerpo me está pidiendo, y ‘no po no debís comer po, estay trabajando’

**P:** obvio

**P:** es menos problemático.

**P:** a mí, en mi caso como… fue que yo hable con mi jefa porque un día me encontraron casi desmayada en el baño y la enfermera, y que era química en realidad ella trabajo como enfermera, y le explico a la jefa y ahí ya me dieron…

**P:** por ejemplo, en vez de salir a las 11 salía a las 10, y a mí entre las diez y las once me daba el bajón

**I:** ¿alguien de aquí trabaja en su casa, como dueña de casa?

**P:** igual nomás porque bueno uno sale… yo salgo todo el día igual, yo cocino pa mi casa, pero igual salgo, voy a la feria, vuelvo, hago el almuerzo…todo igual.

**I:** ¿y cuando uno es diabético y el resto de la casa no?

**P:** Ese también es un problema, hay que hacer aparte

**P:** por eso uno no hace.

**P:** porque hay que hacer aparte, porque es distinto, porque a los otro teni que hacerle un plato bien aliñado porque o sino…ahí quedan

**P:** por eso uno hace las comidas todos igual.

**P:** sí

**I:** ¿usted cocina en su casa para todos?

**P:** no, pa mí no más y para mi nieto

**P:** es fácil pa mí

**I:** ¿sí?

**P:** como a mis horarios, todo

**I:** y si hubiese más gente …

**P:** aaayy, ahí sería otra cosa (*risas*)

**P:** o sea no es que sea por lo caro de repente, lo que pasa es que, si hay más personas en la casa, debería uno cocinarse para uno…

**P:** …y para los demás

**P:** ni tanto (*voz dudosa*), pero es que lo que pasa es nosotros no podemos comer masas, porque se transforman todas en azúcar, las masas

**P:** el pan es uno de los principales

**P:** o sea se puede comer pan, pero medido. Por ejemplo, parto una mitad en la mañana y la otra mitad en la once, ese es todo el pan que me como

**P:** hay diabetis gorda y diabetis flaca

**I:** ¿y cuál es la diferencia?

**P:** da como ansiedad…

**P:** uno no se nota que tiene diabetes, cuando de repente uno ya está mareado, ahí es cuando sabe que tiene diabetes…o que le duele la cabeza

**P:** a mí me da ansiedad

**I:** Ya

**P:** pero ella tiene la flaca porque ella era gordita, ella se adelgazó…ella era más gordita que yo casi.

**P:** Era bien gorda, ahora no

**P:** gordita si era bien gordita, mírela a ella como es ahora (*risas*) yo sigo siendo igual la misma, yo no bajo ni subo

**P:** y no hace dieta tampoco, ¿no hace dieta?

**P:** yo no po

**P:** yo tampoco

**P:** yo no subo ni bajo, yo me mantengo

**P:** pero tampoco hago lo que decía denante la señora Rosa Luz, no es que uno no coma el tomate por no comerlo, pero no como tomate

**P:** yo no como tomate

**P:** y si llego a comer una…

**P:** yo tomo el tomate pa pelarlo y se (irritan) las manos

**P:** yo no tenía idea que el tomate hace mal

**P:** no, si hace mal el tomate, mi marido…

**P:** pero es lo mismo que decía ella. O sea, mientras uno coma en mayor cantidad es lo que hace el problema

**(*barullo*)**

**P:** Para mi es el pan, el pan para mí fue trauma

**P:** me comía un pan entero en la mañana para irme al trabajo, porque después sabía que no podía comer más. Entonces, comerme la mitad del pan con los medicamentos me venían los bajones muy luego. Cuando me dieron la Glibenclamida era una cosa que botaba saliva, salivaba, salivaba, y era una cosa terrible lo que sentía, me sentía muy mal. Eso fue trauma, hasta que mi cuerpo se acostumbró.

**P:** Es el remedio más horroroso que puede existir. Yo no lo tolero, el olor…

**P:** al tragar…es que después, si viene después que usted se toma eso, usted abre la boca y todo el mundo… (*realiza gesto de taparse boca-nariz*)

**P:** es horrible, uno no lo siente

**P:** es que a lo mejor a todos no les pasa lo mismo

**P:** no po

**P:** uno no lo siente

**P:** a remedio. Yo me lo sentía, porque como yo trabajaba en la cocina, entonces yo usaba mascarilla…entonces cuando me dieron eso y más encima que me daban náuseas pareciera que estaba embarazada.

**I:** ¿y el resto?

**P:** no yo no

**P:** es solamente con la gliben…

**(barullo)**

**P:** y son así unas…

**P:** no yo no…

**P:** no, a mí no me hace nada

**I:** ¿y los medicamentos??

**P:** aaaay la metformina es gigante, la metformina es muy grande, pero hay que tragársela no más

**P:** yo no la pude consumir, no…

**P:** viene una que es como larguita, esa me cuesta tragar

**P:** es que depende del laboratorio de repente porque hay unos que las hace muy grande.

**P:** Sí, esas las dan aquí

**I:** ¿y ustedes han avisado que es muy grande?

**P:** si yo les he dicho…

**P:** (*muestra bolsa con medicamentos*) sí, y muchas cosas más

**P:** yo la único que tomo es esa

**P:** no, pero esta es normal, esta uno se la puede tomar porque donde es más larguita, pero hay otro de esta misma metformina que es redonda y es más grande

**P:** esta viene incluida con los remedios…para verlo más grande (*risas*)

**I:** ¿Cómo uno se organiza?

**P:** una montonera (*se escuchan sonidos de molestia*)

**P:** yo los tomo todo al tiro…porque o si no se olvida después

**P:** yo hacía lo mismo, mi niño yo hacía eso…

**P:** después se olvidan po, no hay tiempo

**P:** es que lo que pasa de que la Sra. Rosa Luz me dijo que no

**P:** sí, si no es así

**P:** no debería ser, pero es que si no uno… porque hay que tomarse una en la mañana, la metformina hay que tomarla antes del almuerzo, pero antes del almuerzo, y bueno eehh…no sé qué otras más…

**P:** tres veces al día hay que tomar remedios

**P:** yo a mí me tocan dos

**P:** yo dos veces al día

**P:** yo tomo cuatro en la mañana

**P:** yo me las tomo todas juntas también

**(*barullo*)**

**P:** Porque usted se pone a trabajar y no puede ir a tomarse un remedio porque está en la mochila o está en cualquier parte y no…

**P**: yo me tomo a las 2 de la tarde la metformina y la pastilla de la presión

**I:** Qué pasa ahora ……….

**P:** lo único que dicen uno en la mañana y el otro en la tarde

**P:** claro, y uno se tomo todos esos remedios juntos

**P:** por ser yo la Glibenclamida me la tomo con el Enalapril en la mañana

**I:** ya…….

**P:** y si tengo dolores paracetamol

**P:** insulina

**P:** yo estoy con insulina también

**P:** yo estoy con insulina y tengo las pastillas para la tiroides, tengo las pastillas para…para todo

**I:** y cuando empezaron a inyectarse insulina……..

**P:** nos enseñan ahí

**P:** a mí me enseñaron en el hospital

**I:** ¿ Como es eso?

**P:** No /es peor / risas /

**P:** pero de repente… ¿sabe? Mire lo que tengo yo po (*se levanta polera*) ay no sé si me ve

**P:** quedan moretones en la guata de repente

**P:** depende como se la pinchen

**(Barullo)**

**P:** en el brazo en una vena si se ponen moretones, pero uno tiene que buscar

**(Barullo)**

**P:** es que tiene que haberse pinchado un vasito

**(Barullo)**

**P:** ah no, en las piernas a mí me duele

**P:** en las piernas no… una vez me las intenté poner y me dolió mucho, nunca más

**P:** no, yo no me pongo en las piernas

**P:** Si

**I:** ¿y lo hacen?

**P:** no, yo a mí en el estómago, siempre en derecho nada más

**P:** no nada, nada

**I:** ¿y por qué?

**P:** porque no me duele, para nada… hay una parte como con grasita

**P:** por ejemplo, la señora Guacolda…

**P**: …Sara

**P:** ella y yo nosotros deberíamos solamente inyectarnos en los brazos y en los muslos

**P:** aay no, en los muslos duele

**P:** porque acá el abdomen tiene mucha grasa, entonces la insulina demora más en hacer efecto

**I:** ¿eso lo dijo el médico?

**P:** el médico

**P:** ¿pero ve lo que pasa? Que hay falta de información, o no hay como un libro que diga tal cosa. A mí me decían ‘la insulina en la mañana y en la tarde’, pero uno no toma desayuno todos los días a las 8 y media, ni toma la once a las 8 media porque se pone cada 12 horas la insulina.

**P:** yo tomo a las 6 y media desayuno…

**I:** otro cambio de vida tiene que ver con la actividad física (*risas)*

**P:** si /si también

**I:** ¿Qué pasa con eso?

**P:** yo en mi casa yo todavía no me siento en el sillón o en la mesa a almorzar. De todo el día, he andado pa allá pa acá, pa acá pa allá... Así casi todos los días

**P:** pero a uno le dicen que el caminar en la casa no sirve, uno tiene que salir a caminar 20 minutos y devolverse, lo que uno hace en la casa no sirve

**P:** Cuando el médico me vio, cuando me pinché con la insulina, me dijo ‘usted mijita tiene que caminar una manzana’

**P:** pero dicen que si tenemos que salir a caminar

**P:** sí, pero no nos sirve, el caminar es de que dediques 20 minutos al caminar, sin concentrarse en ninguna otra cosa más que en ese ejercicio que vas hacer. Hay que dar una vuelta a la manzana, y listo.

**P:** ¿y quien tiene tiempo para hacer eso?

**P:** yo no tengo tiempo, pero mi trabajo…

**P:** y aunque esté usted desocupada, en el caso mio cesante… [¿?]pa donde iba a caminar, pero po

**(*barullo*)**

**P:** otra cosa más po, la depresión

**P:** es que hay que buscarse el tiempecito po, de uno no va estar, no yo en la mañana mi desayuno y después mi colación, el almuerzo y ya de ahí a las 3, las 2, salgo a caminar, me doy vuelta la manzana, me doy mi hora

**P:** si po se tiran cajón pa arriba, pa abajo, el saco, que una cosa, otra, la bolsa, echarla arriba de…

**P:** lo otro son los zapatos

**I:** aah ¿qué pasa con eso?

**P:** ese es un tema. Los zapatos, los calcetines o las medias, o lo que usted…o la ropa interior, que ojalá todo diabético usáramos ropa, o sea deberíamos usar ropa que tenga cobre, y eso es carísimo.

**P:** las fibras

**P:** los nylon

**P:** lo que pasa que, en los pies, por ejemplo, el caballero que está tantas horas de pie y trabajando, el diabético se moja, le sudan

**P:** le transpiran

**P:** le suda le transpira mucho el pie

**P:** no y uno suda completo

**P:** entonces esta parte de acá, entremedio de la entrepierna, o en…

**P:** en los pliegues…eso se moja, se empapa

**P:** nosotros los diabéticos no debimo usar calcetines con costura porque nos molestan…no puede, tiene que ser el calcetín sin costura

**P:** se pone muy delicado

**P:** y zapatos como de cuero y todas esas cosas que a uno le digan, que tienen que ser con badana, de esas…

**P:** yo en mi casa…si ustedes vieran mi casa, tengo caleta de botas, zapatos… ‘me dice tení tantos cachureos ahí’, es que uno va cambiándose, cambiándose, porque y hasta que el zapato no le duela, ‘bueno son mis cosas’ les digo a mi marido

**P:** son mis zapatos, yo tengo chalas, que tengo zapatos, que tengo que…al final tengo que ir cambiándome, cambiándome…

**I:** ¿y cuidarse los pies?

**P:** si po

**P:** y eso es otra cosa

**P:** acá hay podólogo

**P:** sí, pero hay que tener audiencia para que te atienda el podólogo

**P:** si po pa que la atiendan a uno, yo que estoy pidiendo ya…

**P:** por ser hoy día, ¿pa cuando me dieron? (*pregunta a P6*)

**P:** pal 31 de mayo

**P:** ahí vienen 30 días

**P:** pero eso nos dieron ahora y nosotros veníamos pidiendo hora del mes pasado

**P:** y después no hay podólogo de nuevo

**P:** ese día me dijo que no me cortara las uñas, de los pies, anoche tuve que decirle que me las cortara ¿por qué? Porque ya duelen tenerlas largas

**P:** Yo soy corta de vista, y pa cortarme las uñas siempre me saco sangre (*sonido de impresión*) e igual trato ya ahora no de cortármelas, porque antes me sacaba la punta de los dedos de aquí

**P:** yo soy podólogo

**Risas**

**P:** no necesito me las saco yo sola (*se ríe*)

**I:** ¿qué pasa con la familia?

**P:** lo único que a uno le dicen ‘ya entraste al club de nosotros’, nada más

**P:** soy yo sola y mi marido…

**P:** ‘¿no teni otra cosita?’ (*en tono irónico*)

**P:** o cuando a uno le dicen ‘aaay es que tu soy la porfiada que no te cuidai, que ya estamos cansados que no te cuidi’ que…esas son las palabras de ellos

**P:** de los hijos

**P:** Así son los hijos

**P:** ‘cuídate es tu problema’. Mi hija le dice a mi otra hija ‘oye sabi que mi mama está enferma’ y ella ‘bueno si mi mama no se cuida po, ¿qué querí que le hagemos? Si el problema es de ella’

**P:** a nosotros por lo menos no. Al contrario, los hijos andan preocupados, ‘¿te pusiste la insulina? ¿te tomaste las pastillas?’ O si me siento mal: ‘Cuídate quédate un ratito sentada, ya no hagai ninguna cosa, mejor descansa’, pero siempre la preocupación de la…

**P:** a ti como que la tiraran pa arriba, a ti como la tiran para abajo

**(Barullo)**

**P:** y así uno empieza también a cuidar. Yo tengo cuatro hijos, tengo dos hombres y unas gemelas, y hay dos de ellos que tienen resistencia a la insulina, o sea que han estado con el pre diabético por un sistema de peso de mala alimentación.

**(*suena teléfono*)**

**P:** dígale que ‘ando pololiando’, dígale.

**(*alguien habla por teléfono*)**

**P:** pero que es lo que pasa que yo, hemos tratado, yo en lo principal he tratado de con mis nietos, porque me ha tocado cuidar 3 de 4 que tengo, de irlos cuidando en el sentido de que no hagan lo que hicimos nosotros. Porque uno, estas enfermedades parece que no se conocían antes, todas las enfermedades parece que no se conocían porque no eran… Entonces qué es lo que pasa, que hay que cortar el círculo en alguna parte, y nosotros lo hicimos con los niños. Yo por lo menos, que es la que los empezó a cuidar, porque claro…uno cuando va al consultorio: ‘no le de…’ eeh los niños no necesitan azúcar, y resulta que uno ha sabido siempre que dándoles azúcar funcionan en la mañana, para el colegio lo necesitan. Alguna vez que me dijeron que les diera la leche con durazno

**I:** ¿Qué?

**P:** Juanito que es…

**I:** ya ¿y para qué? ¿Qué es…?

**P:** para darle sabor a la leche, en vez de azúcar, en vez de saborizante, que se empezó a usar mucho todas estas cuestiones que les dan a los niños de saborizante

**P:** Claro, entones todo esos traen un tipo de cosas que…como todo orden de cosas hay algo que hace bien, pero a la vez hace mal en otra, en otro aspecto

**P:** entonces con los niños uno trata de qué, de no las frituras, de no… yo soy de hacer pan amasado con chicharrones

**P:** sí

**P:** pero los hago yo pa lo grandes, pero a lo niños trato de darles…

**I:** ¿y ustedes? Porque ustedes son los dos diabéticos

**P:** si, pero nosotros comemos igual, no nos preocupamos

**P:** hacerse el leso no más

**P:** nada más

**P:** no yo le agregaría, que ustedes que están interesados en esto, que sacaran una especie de libro, de folleto informativo

**P:** cómo empieza (*la enfermedad*), cuáles son los síntomas…por ejemplo cómo empieza, cómo se puede dar cuenta una persona que tiene diabetes: en la sudoración ¿ha? En el dolor de los pies en la noche, en el calor de los pies, en la picazón de las manos, en el dolor que empiezan a doler las uñas

**I:** ya

**P:** la transpiración continua también

**P:** claro, es en lo que es la sudoración. Y en la noche que usted despierta y despierta desesperado y esta mojada entera ¿ha? y sin haber ningún motivo

**I:** ¿y cuando ustedes vienen a control?

**P:** te dicen ‘¿qué estay tomando?’ y uno les dice que está tomando, ‘ya entonces te damos lo mismo. Ya chao. Que pase tal persona’

**P:** o a veces le suben la cantidad de azúcar, o sea de…

**P:** de los medicamentos, claro, le suben generalmente la glicemia… o sea, la cantidad de insulina.

**P:** (alguien se despide) ¿se van a ir?

**P:** si

**I:** algo, ¿alguna otra cosa?

**P:** yo creo que, siendo un poco metódica, porque uno sabe que tiene en las mañanas que tomarse sus medicamentos, comerse la dieta, por ejemplo, yo me tomo una taza de leche con medio pan, yo se que con esa cantidad (*ruido*) yo se que con eso llego a mi trabajo, porque cuido a una tía que tiene Alzheimer, allá me como otra mitad de pan tomo otra tacita de té y después almuerzo entre 12 y media y 1.

**P:** a mí la Sra. Rosa Luz, porque a mí me viene más baja el azúcar que alta, a mí casi me dio un coma por baja de azúcar, es muy desagradable, es terrible. Y la Sra. Rosa Luz me dijo a mí que no me comiera medio pan, que me comiera un pan entero porque era muy poco al desayuno, pero yo sé que, si me como el pan entero de una vez me sube el azúcar como a trescientos, entonces me lo como fraccionado, y ahí se me mantiene más o menos el azúcar y no me sube a…

**P:** no si dieta dieta en si el nutricionista no da

**P:** es la… porcionar los…es la cantidad de las comidas

**P:** no comer en exceso

**P:** las porciones de comida ¿ah? Y yo le pongo un caso, al caballero. Eeh… trabaja todo el día, no comió en todo el día, llegó en la tarde y como es la señora le va a servir un cucharón de comida…va a quedar con la misma hambre y al rato se va a desmayar

**P:** pero cuando le da por comer todo el día, anda todo el día comiendo, que una fruta, que un poco de pan, que va y se levanta a comer, a tomar leche…

**P:** y en la noche me levanto a las tres, dos de la mañana

**P:** y eso también está mal

**P:** está malo, pero el cuerpo me lo pide

**P:** igual que la sal uno trata de cocinar con poca sal, pero, los que ya estamos con esta edad de viejos, uno esta acostumbrado a comer toda la vida a comer de todo con sal y con todo, entonces es difícil ir haciendo lo que le dicen a uno, porque no solo la diabetes, la hipertensión nosotros no necesitamos sal

**P:** yo por ejemplo soy diabética e hipertensa

**P:** y ahí también…yo también tengo lo mismo. Cuesta porque hay que hacer…

**P:** cuesta mucho

**P:** para mí no me ha sido tan difícil porque mi marido, lo que yo como lo come él, porque a él le dio un infarto, entonces…

**P:** y cuando no le había dado el infarto, comía lo mismo que yo: sin sal, con poca sal, y le sirvió mucho cuando le dio el infarto, porque el ya no sufrió el cambio de dejar la sal, ya se había acostumbrado a comer con poca sal, come todo light, con poca azúcar, no consume azúcar, come Stevia, entonces ahora está intolerante a la insulina entonces no le ha sido difícil a él ese cambio. Y en mi caso, mi mamá fue siempre diabética y ella como que nos inculcó a nosotros a comer menos azúcar menos sal, yo desde ahí…

**P:** Claro, para mí no fue trauma

**P:** claro, pero es que si uno hace la dieta como corresponde y se toma los medicamentos y come moderado como dice, el azúcar no se le altera. Es cuando el azúcar se altera, ahí es donde vienen las complicaciones, que le duelen las piernas, que les cuesta caminar, que ese cansancio…el sueño, da un sueño que uno no lo aguanta, se queda dormida en cualquier lado. Esa es la verdad de las cosas.

**P:** si uno se sienta en un sillón y se va a reposar, se queda listo

**P**: es un sueño muy terrible cuando está la azúcar una poco alta

**P:** no, yo soy mala para dormir

**P:** a mí me dio la diabetes, tenía 55 cuando me la encontraron aquí

**I: ¿**qué es lo que ha hecho todo este tiempo?

**P:** cuesta porque a veces la tengo alta, ahora estoy con insulina aquí en la guatita

**P:** y también cuando la hemoglobina está alta, a uno le da mucho sueño

**P:** y después llegar hacer las cosas a la casa, lavar, planchar…

**P:** me levanto a las 6 de la mañana, llego a las 5 de la tarde, después llego hacer las cosas a la casa, hacer aseo, y al otro día lo mismo

**P:** tener que lidiar con el transantiago, ese es otro suplicio, esa es una enfermedad más

**I:** a mí me quedo súper claro

**P:** yo creo que es terrible esta enfermedad, lo que pasa es que uno empieza a como aceptarla, como que empieza a…

**P:** como que convive ya

**P:** …a convivir con la enfermedad y aceptar las molestias que dan…porque no hay de otra po, está y ya no

**P:** y no, cuando da fuerte, da fuerte

**P:** y lo otro que uno se cambia el apellido después

**I:** ¿Cómo así?

**P:** yo me llamo Julio Dolores

(*risas*)

**P:** aaay que me duelen las piernas, que me duelen las…

**P:** porque todos los días, si no son los ojos, son los muslos

**I:** Pero ¿es muy difícil hacer los cambios?

**P:** es que lo que pasa…

**P:** a veces sí, porque el trabajo incluso el mismo trabajo po

**P:** el mismo trabajo lo hace

(*preguntan si ya se van*)

**I:** se los agradezco, se los agradecemos muchísimo de verdad yo creo que esto ha tenido un valor enorme.

**P:** A veces yo creo es un poco mas de parte de los médicos, mas información, porque de repente ven los exámenes y ya, ‘este está malo, este está malo’, ‘teni que cuidarte chiquilla’

**P:** nos mandan hacer exámenes de sangre y de orina no saben más

**P:** pero no hay una especificación, así como la Sra. Rosa Luz, es muy buena para explicarle a uno, ella te dice por qué le pasa esto, qué tienes que comer…

**P:** yo por ejemplo estoy preguntando hace mas de…1 año y tanto saber qué grupo de sangre tengo, porque yo no me explico que no hay, yo no encuentro ni la causa, ni el motivo, ni la razón, de que tenga diabetes ¿ya?, y en segundo de que se me dispare así, sin haber motivo ninguno, no paso rabias…

**P:** eso iba a decir, de repente es tensional también

**P:** si po si esa es la que yo tengo

**P:** y lo otro usted pasa una rabia y le sube, pero al cien por ciento

**P:** una rabia que pase uno

**P:** o si tiene pena

**P:** no y si tiene una alegría fuerte también es lo mismo

**I:** muchísimas gracias……

**HEALTH CENTER 1- PATIENTS**

**Investigador (I):** Quiero que me cuenten desde cuando les dijeron que eran diabéticos, ¿qué pasó con sus vidas?

**Participante (P):** lloré harto, lloré mucho

**P:** sí

**I:** ¿Por qué le dio tanta pena?

**P:** porque tengo familiares que les han cortado las piernas, compadre le han cortado las piernas…

**I:** ya

**P:** …y heridas que no se sanan, y todo eso. Entonces, pa mí me ha dado mucha angustia

**I:** desde el principio

**P:** sí

**I:** ¿Qué ha pasado desde ese momento?

**P:** eeh... bueno, empezando tengo a mis hijos que son reacios a los remedios, me retan mucho porque yo tomo remedios

**I:** ya

**P:** y un día a uno de ellos lo llamó el psicólogo y le puso las cartas sobre las íes,

**I:** exacto

**P:** porque no podía ser eso que me estuvieran gritoneando por los remedios

**I:** exacto. Ok, ¿qué ha pasado con el resto desde cuando partieron con esto?

**P:** eeh... con mi último hijo que tiene 38 años quede diabética. Yo no tenía, o sea mi mamá era diabética.

**I:** ok

**P:** pero yo no vivía casi con ella, siempre la iba a ver y todo y sufrió mucho

**P:** y cuando yo tuve al niño me dijeron que era estacional

**P:** pero yo no tenía idea. Entonces, yo vivía, cuidaba una parcela acá en Lo Cañas

**I:** ya

**P:** yo vivía trabajando, viendo a mis niños porque el primero que lo tuve yo, eemm… nació el niño con…problemas, o sea tengo un niño limitado

**I:** ya, ¿Cuántos hijos tiene?

**P:** tengo tres

**I:** ¿y que paso con usted cuando le dijeron que era diabética?

**P:** nada po, seguí comiendo igual, trabajaba, hacía el aseo, llegaba a traspirar limpiando la casa, le ayudaba a mi marido por…

**I:** ¿y remedios le dieron?

**P:** No

**P:** ¡no!, no me explicaron

**I:** nada

**P:** porque si me hubiesen explicado usted se tiene que… mi hija tuvo su hija y también tenía lo mismo…. y se le pasó

**P:** también me pasó lo mismo, pero se me quitó, pero ahora me dio me dio, después de lola me dio

**P:** entonces, yo no me supe cuidar

**P:** ¡!no tenía idea!!

**P:** en el caso mío cuando me lo detectaron a mí hace como cinco años atrás, yo dije “ya, tengo diabetes, no estoy ni ahí”

**P: (mama del 3):** no se cuidó, no tomaba remedios nada, nada

**P: (joven):** nada, llegue a estar flaco, flaco

**P:** sí, adelgazó

**P:** y después de ahí me metí en otra cuestión que… en la… la droga

**P:** me metí en la co-co-cocaína **[dificultad para decir palabra]**

**P:** por lo mismo, me dio eso, después enflaquecí, llegué a pe-pesar 70 kilos

**P:** un año y medio internado en…

**P:** por la adicción, un año y medio

**P:** en la “Si/o”

**P:** tenía que ir a dejarlo e ir a buscarlo yo todos los días, todos los días

**P:** sí

**P:** cuando me dijeron que…fui yo a todos los controles que tiene de diabetes, yo vengo con él, yo no lo dejo solo

**P:** y entro con él, él me dice “no yo entro solo”, “no, yo tengo que entrar contigo”, y ahí la... cuando me dijeron la primera vez que tenía que inyectarse, me explicaron, le explicaron a él cómo había que hacerlo, y de ahí que yo estoy dándole remedios, inyectándole en la mañana, en la tarde, y de ahí yo no lo he soltado más. El problema que lleva casi un año, casi un año seis meses con licencia, no ha podido ir a trabajar, ahora la doctora le dio quince días más se le termina el 30 la licencia, pero ahora empezó con heridas en los pies de nuevo.

**P:** por la herida de los pies

**P:** por la herida de los pies y por estar internado

**P:** sí

**P:** los pies

**I:** ¿sí? y usted ¿sabe cómo cuidarse?

**P:** ¡!sí, sí!! me dijeron

**P:** es que se dejó

**P:** empezó después, como se vio mal cuando supo que tenía diabetes ya empezó con el otro problema

**P:** lo dejó a un lado

**P:** de ahí me fui… me… me…. me llegué a ahorcar po **[dificultades para hablar]**

**P:** sí, tres intentos de suicidio

**P:** por lo mismo

**P:** y otra que se enamoró también, de una persona que no era para él, y esa persona estaba metida en droga y esa lo metió a él con drogas

**I:** ya……

**P:** sí, para olvidarme

**P:** porque me me-me-metieron mucho en cabeza a mí que la diabetes es muerte en vida

**P:** yo escuché por ahí

**[Risas]**

**I:** Y usted, ¿cómo vive la diabetes?

**P:** hace na mucho de esa vez que estuve en el hospital no mas recién, y ahí me la cortaron **(la enfermedad)** al tiro

**I:** ¿con qué?

**P:** sí, me dieron unas pastillas tremendas de grandes

**P:** si, molestan pa tragárselas, pero…

**P:** Es difícil tragárselas porque es así **[señala tamaño grande]**

**[Risas]**

**P:** no es tan grande

**P:** ¿la metformina?

**P:** sí, no es tan grande

**P:** es grande

**P:** sí

**P:** la Metamorfina debe ser

**P:** yo, bueno, hará hace como unos años no más

**I:** ¿usted se dio cuenta?

**P:** no, se dieron cuenta en el médico

**P:** el doctor me atendía a mí y él me dio los medicamentos para… tengo que tomarme 2, 4, 5 pastillas diarias

**I:** ya ¿en diferentes horarios o no?

**P:** Sí

**P:** es jodido, porque uno anda haciendo diligencias por ahí y como tiene que tomársela a la hora y a veces se atrasa. Entonces no puede llegar a tomarse las 2 pastillas, va echando una pa atrás. Entonces no es como cuando uno está permanentemente en la casa y no tiene nada que hacer entonces uno ahí puede ir a la hora… entonces **[no se entiende, hablan con voz baja dos personas]**

**I:** ¿y cuando uno trabaja?

**P:** ese es el problema mío porque…

**P:** y lo otro que…

**P:** porque cuando el entra a trabajar se va a la cinco de la mañana, y yo a las cinco de la mañana no lo puedo escuchar

**P:** eso es lo otro, porque el doctor dijo que tenía que tomar por vida esos remedios

**I:** claro……

**P:** estar jodido de la pierna, porque con esto me pase a llevar el fierro **[señala su pierna]**

**I:** ya

**P:** harán más o menos como unos 5 o 6 meses atrás

**P:** y me saque el pedazo po, me saque el pedazo me quedo una canaleta, así como la punta del dedo

**I:** ya

**P:** y ahí que me han estado curando aquí y no…

**P:** de a poco dice la señorita que va sanando, pero de aquí a que sane, porque me sacó con carne y todo

**P:** por la cuestión de la diabetes po

**I:** ¿cómo andan sus controles de diabetes?

**P:** no, la azúcar al menos a él le bajo porque no bajaba de 350, 400

**P:** ahora tengo 160, 130

**P:** 125

**P:** llegue a te-te-tener 61

**P:** yo llegué a tener 36

**P:** claro, porque si me… o sea emm… como poco, o sea como verduras y pedacito de pollo

**[Risas]**

**P:** yo me inyecto a las siete de la mañana, mi marido me pone la inyección. Eso es lo que quería saber yo, porque me la pone aquí en la nalga, porque me ponía tanto aquí que ya no daba más

**I:** ¿qué es lo que le han explicado?

**P:** sí, en esta parte

**P:** no, no se coloca ahí

**P:** se coloca aquí en los brazos, en la guatita y aquí

**P:** no, aquí me duele, pero llego a tiritar porque a veces me la pongo yo la de la tarde

**I:** ya

**P:** porque él me inyecta a las siete, a las siete y media me lleva el desayuno

**I:** ¿pero les enseñaron?

**P:** sí, acá en la…

**P:** sí

**P:** ¿sabe lo que a mí me dijeron…?

**P:** no, no siempre, sino que en lo gordito aquí

**P:** sí, en esta parte

**P:** lo que me dijeron a mí que en los glúteos no porque hay muchas venitas

**P:** no, si le dije a la doctora Valdivia porque ella me dijo y me dijo que estaba bien

**P:** qué raro…

**P:** pero el brazo no

**P:** porque los brazos, sabe que tengo…tengo

**P:** pero es que lo que pasa es que al ponérsela en los brazos a ustedes tienen que apretarle así

**P:** que la aprieten así

**[INTERRUPCIÓN: VIENEN A BUSCAR A UN ENTREVISTADO]**

**P:** ¿a mí me vienen a buscar o no?

**P:** no al caballero vienen a buscar

**P:** tengo reunión en el adulto mayor

**I:** ah nos quedan poquitas preguntas. ¿Qué pasa con la alimentación?

**P:** ahí estoy en la alimentación, yo hago comidas de las que hago siempre y él come de la que hago yo

**I:** ya…

**P:** poca cantidad, así como hay días que come harto, hay días que no quiere comer

**I:** ¿es la misma comida que comen todos?

**P:** o sea… el té sin azúcar, pero el que se puede tomar siempre échele un poquito de endulzante, endulzante o en la tapa esa de los lápices

**P:** claro, de azúcar

**P:** del lápiz Bic

**P:** la sal o la azúcar

**P:** la comida la doctora me dijo que podía darle de lo mismo mío, pero todo el fondo del plato

**P:** y me da la medida de los alimentos… si estoy con los alimentos que les dan aquí a los pobres (alimentos del CESFAM)

**I:** ¿pero comen lo mismo que el resto de la familia s?

**[Algunos participantes dice: “no, lo mismo”]**

**P:** media taza

**P:** y el caldo lo que usted quiera

**P:** una sopa cualquiera, pero lo que pasa que el caldo puede ser harto, pero lo que es arroz, fideos, es media taza

**P:** media taza, nada más

**P:** y ensalada

**P:** y harta ensalada

**P:** harta ensalada verde

**P:** hay que echarle lechuga a la agüita no más como lo hago yo

**I:** ¿cómo es eso?

**P:** en una cuestión de plumavit, una fuente, le echo agüita y en una tapa de plumavit le pongo algo, lechuguita así, y le pongo con una esponja la raíz y… pero el agua hay que estarla corriendo si para que crezca la lechuga

**P:** sí

**I:** ¿cultiva la lechuga?

**P:** y es sanita, es limpia

**P:** eso se llama hidropónico, hidropónico

**P:** Sí, pero en este tiempo se helaron, mi marido tenia plantadas y se helaron

**P:** si usted las echa a campo libre sí, pero yo las tengo bajo techo

**P:** es que yo tengo un predio muy chico

**P:** yo las tengo incluso arriba de la mesa y esta con techo, así que no les pasa nada, no se pueden ahogar, no entra nada de frio…la única gata soy yo que me la comí

**[Risas]**

**I:** ¿Qué es lo más difícil de tener diabetes?

**P:** que uno no puede hacer una vida de ir a fiestas, de tomar, de…eso, todo queda, para mi quedo todo…bueno yo eeh...… cigarros, ni tragos, ni tanto a fiestas no porque vamos a partes de la familia nada mas

**I:** ¿y la familia?

**P:** no, conversamos no más

**P:** el cigarro hace mal para la diabetes

**P:** sí

**P:** y fumar, el olor del cigarro ya no lo soporto

**I:** ya

**P:** claro, como antes ya no

**P:** porque uno siempre anda como achacada digo yo

**P: [sonidos de quejas]** “ando achacada que querí que le haga”

**P:** además, que andan otros recuerdos por ahí, más me achaco

**P:** de pena

**P:** ¿con una diabetes duerme mucho la gente?

**P:** porque él duerme todo el día, se levanta puro para ir al baño, se levanta solo para almorzar, y dormir, dormir

**I:** y ¿cómo ha estado el azúcar?

**P:** no, si antes yo le tenía que hacer la glicemia en la mañana, al almuerzo y en la noche

**P:** ahora no le hago casi…ahora se la tomaron cuando tuvo control y tenía 120, 120 algo por ahí, la doctora dijo que estaba bien, le había bajado bastante pero todavía falta si

**P:** todavía falta un poco

**P:** lo que pasa que el azúcar baja y sube, baja y sube

**P:** Es un ascensor

**I:** ¿ascensor?

**P:** eso es lo que me dijo la doctora a mí, que era un cáncer… ¿cómo era?... un cáncer silencioso que uno tenía

**P:** Sí, cáncer silencioso podís estar uno un día muy bien y al otro día muy mal

**I:** ¿han escuchado es frase?

**[Los participantes dice: “Sí”]**

**I:** ¿y para usted?

**P:** no

**P:** cuando, a ver cuando estuve en el hospital, me la quitaron en el hospital y después me lo pararon

**I:** ¿y comida especial…?

**P:** nada

**P:** claro si, yo me operé… llevo cuatro meses operado del corazón, me abrieron… totalmente

**I:** aaah

**P:** tenía tres

**P:** sí

**P:** ahora tiene que cuidarse por la diabetes sí

**P:** sí, tengo control

**I:** ¿les han explicado de que se trata bien la enfermedad?

**[Los participantes concuerdan: “no”]**

**P:** en mi casa no creen que yo soy diabética porque… yo hago todo, lo que es atender a seis personas, más encima mi hijo que es enfermo que él no se le nota, pero… **[Sigue hablando de fondo]**

**P:** lo único que me dicen los médicos no más, hay tres niveles de diabetes

**I:** ya

**P:** hay tres niveles, la doctora me dijo que lo mío era diabetes de tercera, la que estaba recién empezando

**I:** aahh,

**P:** hay tres clases

**P:** la del principio, claro

**P:** eso es lo que le iba a consultar, ¿Cuál es la diabetes más mala? ¿la I o la II?

**I**: habla del origen de la diabetes

**P:** de dónde viene

**P:** yo trabajo con chocolates

**P:** en M&M

**P:** ahí comía harto antes

**P:** 22 años

**P:** entre a trabajar a los 17 años ahí

**P:** y mi marido trabaja en 2en1

**[Risas]**

**P:** yaaaa

**P:** puros dulces en la casa

**P:** sí, yo sí

**P:** llegó a pesar 150 kilos

**P:** cuando manejaba, o sea manejo ahora eeh...h la guata se me quedaba en el volante

**P:** sí po, hacían eso para que no los robáramos

**I:** ah, mejor te dan para que no les roben

**P:** sí po

**P:** y lo que pasa es que él viajaba harto, se iba semanas enteras con un camión

**P:** a Puerto Montt

**P:** a entregar cosas

**I:** aah…….

**P:** se bajaba en el camino a comer cosas

**P:** …y abríamos y sacábamos dulces

**I:** ¡ooooh

**P:** sí, sí hay. Hay como 6 con diabetes

**I:** ¿cómo se organizan para venir aquí al CESFAM?

**P:** yo me, yo me he venido porque…. estoy con licencia

**P:** estoy con licencia por eso ha podido venir

**P:** no, yo no vengo

**P:** dice que no le dan permiso, y no viene

**I:** aah

**P:** a mí por la diabetes nunca me han citado aquí

**P:** no queda otra

**P:** gracias

**I:** gracias a usted, muy amable

**P:** todavía me duele las piernas aquí de lo de la extracción

**P:** yo creo que en los trabajos tienen que darles permiso para médico

**P:** tendrían que darle

**P:** según el criterio de los jefes…

**P:** de cada patrón

**P:** mi marido dice que no, que en todos los trabajos le tienen que dar permiso cuando tienen que ir

**I:** ¿y qué pasa cuando uno trabaja y los remedios?

**P:** ahí por el lado mío no hacen mayor problema

**P:** esa es la parte jodida, no solamente cuando es trabajo sino cuando tiene que hacer diligencias, que lo pilla por allá uno dice “no si en una hora voy y vuelvo”

**P:** yo me los tomo en la mañana, me tomo dos, una a las siete de la mañana y la otra a las ocho. Después, me tomo la metamorfina, esa me la tomo media hora antes de almuerzo

**I:** ya

**P:** y la otra en la noche…antes de acostarme

**I:** ¿y qué pasa cuando uno además tiene otra enfermedad?

**P:** ahí hay que hacer el atado de remedios

**I:** ¿un atado?

**P:** claro, porque en la noche yo me tomo la pastilla pa la presión…

**P:** eso también me lo han dado…

**P:** claro, la pastilla pa la presión, tengo el colesterol alto…son como cinco pastillas en la noche

**I:** ¿y en el día?

**P:** cuatro en la mañana

**P:** y dos al almuerzo

**I:** aah…

**P:** yo me llegué a tomar 15 pastillas

**P:** él se toma tres en la mañana, cinco al almuerzo, y cinco en la noche, aparte de la insulina…

**I:** ¿todas juntas?

**P:** las cinco juntas, si porque está tomando…Yo tomo 1 en la mañana porque soy hipertensa pero ando bien, en la mañana y la insulina

**P:** Esa es para… la que le dan pal corazón a uno esa se la puede tomar a cualquiera hora

**P:** porque él toma aparte la olanzapina y otras **[pastillas]** más

**P:** las que me dio el sicólogo

**P:** claro, por eso no salgo mucho. Cuando voy pa donde mi hijo no más, en la noche o quedarme allá, porque sé que me van a hacer atado por los remedios mi hijo más chico, pero cuando voy donde mi hermana tengo una bolsita y echo todos los remedios que tengo que tomarme en la noche

**P:** Y si uno sabe lleva su bolsita po

**P:** sí po, en realidad uno tiene que andar con todo **[risas]**. La farmacia ambulante

**P:** yo cuando he ido a la playa y me he llevado mi bolsita, allá la ponemos en el refrigerador

**P:** y mis remedios…

**I:** ¿algunos exámenes que se tengan que venir hacer acá?

**P:** eeh... sí, yo cada tres meses exámenes

**P:** no hay que venir en la mañana, de siete y media a ocho y media reciben, nada más

**P:** en ayunas

**P:** en ayuna

**[INTERRUPCIÓN: GOLPEAN LA PUERTA]**

**P:** si yo me estoy tomando una pastilla que son una…

**P:** eeh... son unas, cuantos se llaman… ¿hormonas pueden ser?

**P:** son hormonas, que me están dando porque yo tengo tumores, entonces me estoy tomando unas… la mitad de la pastilla despertar y pa adentro

**I:** aah una pregunta más: ¿qué pasa con la parte de actividad física?

**P:** caminar, hacer ejercicio

**P:** hay que hacer ejercicio, bastante. Porque en los clubs, la mayoría de los clubs están haciendo terapia, dos veces en la semana. Van niñas de acá.

**I:** ¿sí?

**P:** de acá mismo a los clubes hacer terapia

**I:** ¿cómo lo hacen?

**P:** los martes y los jueves van las niñas

**P:** él no hace

**P:** nada

**I:** ya

**P:** Pero yo camino, salgo a caminar en las tardes, antes porque ahora no, porque llegaron puras tomas, puros marihuaneros…tenía un parque precioso, más de 80 plantas, ¡me las arrancaron con máquinas! y pusieron… se puso una industria de camiones, ¿sabe lo que es estar en la mañana tiritando toda la casa? Yo lo sé… yo lo sé, ¿y sabe qué? El alcalde dice conservar los arboles, yo planteé al frente que es zona de inclusión… **[habla con voz de lástima]**

**I:** ya

**P:** …era zona de inclusión, árboles y arbustos porque en Lo Caña yo hacía, porque después de coser ya después me fui para allá y ya no me siguieron las de acá de La Higuera, y me vine para acá ¿y sabe qué? traía plantas, y antes por ser eran mejor 18 palmeras, eran eeh... árboles, árboles ¿y cuánto se llama?, estos me los sacaron porque estaban más al rincón, estos árboles grandes ¿Cómo se llaman? **[dice un entrevistado: ¿álamos?],** estos árboles firmes…ay

**I:** ¿tiene lugares para salir a caminar?

**P:** no, ahora no

**P:** sí, yo encuentro que acá sí

**P:** yo vivo por Walker Martínez con Av. La Florida **[Habla al mismo tiempo que otro P:]**

**P:** se tomaron los sitios, y…

**P:** yo camino harto

**P:** la gente pasa borracha, ahora venden marihuana más abajo…eso no me gusta

**P:** yo de Walker Martínez con Av. La Florida voy al catorce de a pie, voy al hospital a pie Florida **[Habla al mismo tiempo que entrevistado 2]**

**P:** y más encima están dando permisos para que la gente crie 6 plantas por sitio

**P:** más lento si, camino más lento, pero camino Florida **[Hablan al mismo tiempo]**

**I:** y usted mi caballero ……..

**P:** no, yo por el pie es poco lo que camino, pero siempre me movilizo, sí

**I:** ya

**P:** a pie una o dos cuadras, no camino mucho

**P:** caminar

**P:** por los pies

**P:** siento abajo como que anduviera en maicillo

**P:** sí, igual, aunque sea bueno el zapato igual, no siento… la mitad del pie no lo siento

**P:** lleva un año y medio afuera, entonces no ha ido al trabajo, puro tirando licencias

**P:** más control deberían hacer

**P:** más controles sí, porque son cada tres meses

**P:** claro

**P:** no, yo tengo máquina

**P:** yo acá yo igual tengo máquina

**P:** yo, por ejemplo, tengo máquina…

**P:** …no yo tengo máquina y me tomo casi toda…

**P:** no, yo al menos no

**P:** por ejemplo, mi hijo tuvo control ahora en mayo, el 19 de mayo, y ahora tiene control en agosto

**I:** … y si no tienes la máquina que pasa en esos tres meses

**P:** más seguido

**P:** porque yo vine en diciembre del año pasado a control y me dieron las órdenes para los exámenes pa este mes, recién me dieron hora para el 23 de junio para que me vea la doctora, entonces eso es mucho…. Florida **[Habla al mismo tiempo que entrevistado 4]** cinco meses…

**P:** imagínese de podólogo el vino en abril…en abril vino a podólogo, y ahora me dijeron que tenía que venir a pedir hora recién en noviembre

**P:** yo tengo que pedir este mes

**P:** yo pedí hoy día, iba a ver si tenía podólogo en noviembre

**P:** ¿en noviembre?

**[Risas de indignación]**

**P:** a mí me atendió hace dos meses atrás, tres meses atrás, y ya hoy día me dieron hora para ahora para el primero

**P:** yo pasé a pedir hora para él y me dijeron “no porque aquí tiene que venir en noviembre”. No y sabe lo que es cara son las cuanto se llaman, las tiritas…pa cargársela

**I:** aah, tienen la máquina, pero son caras las tiras

**[Los participantes concuerda: “sí, son caras las tiras”]**

**P:** si, vienen veinticinco tiritas y valen dieciséis mil y tanto. Antes las daban en los consultorios, antes daban acá todo, pero después ya no, no dan, ahora no dan eso, no tenemos…

**P:** hay harto remedio que falta aquí

**P:** no, y uno tiene que estar comprándose, yo me tengo que comprar los remedios para el colesterol, pero no es el colesterol que tengo malo, si no que son los triglicéridos

**I:** ¿ya?

**P:** entonces tengo que comprar y me dio la doctora por un año. Yo encuentro que es mucho ya me llega a doler el estómago, me los tengo que tomar en la noche ya cuando me voy acostar y ahí me empieza…

**I:** ¿y otros remedios?

**P:** no, pero nosotros no hemos comprado

**P:** algunas, sí

**P:** yo me tengo que comprar

**P:** lo único las plumillas esas de la maquina que hay que comprar

**I:** claro

**P:** las plumitas

**P:** y que 25, al menos yo que tenía que ponerle tres veces al día de veinticinco no duraban nada

**P:**  a mí me hicieron un seguimiento todos los días, a cualquier hora

**P:** sí, eso le hicieron a él

**P:** y tenía que tomármelas, así que imagínese

**I:** bueno, muchísimas gracias, es súper importante saber esto

**P:** los remedios son muy caros, es muy caro.

**P:** Lo que pasa es que a veces el doctor, yo a veces vengo a los controles con él **[señala a pariente]** y entro a los controles con él. Pero a veces viene a control y le pregunta cómo estai, por si acaso lo examina, no le hace nada.

**P:** A mí estuvieron un año sin verme, ni un médico no me veía, ni la nutricionista, nadie, ni un control

**I:** ¿y por qué?

**P:** tuve que pelear, me daban los remedios todos los meses…

**P:** pero no tenía control, entonces eso no puede ser

**P:** yo por ejemplo lo atendía otra doctora y yo después pedí para la doctora Astudillo, la doctora Astudillo lo examina entero, es súper buena, a mí me…a mí me vio…

**P:** aquí cuando… el problema es cuando nos mandan el doctor a uno a pedir hora lo atienden y le dicen “vaya a pedir hora ahí, hay que ir”, uno va allá “no la agenda no… tiene que esperar que llegue la agenda para pedir hora”, y cuando uno viene a pedir hora le dicen “no va a tener que esperar no mas porque la agenda está completa” y así nos van…

**P:** En el Sotero me paso lo mismo, me operaron en febrero del tumor del ojo acá en la clínica, me tuve que operar por el AUGE a todo esto por que en el Sotero me patearon para todas partes, pero todavía no tengo control

**I:** ah…

**P:** …pero le dije de los lunares a la doctora Valdivia y me dijo que no eran… porque me pica mucho la espalda y tengo 2 lunares, estos hacen como 15 años que me iban a sacar esto, me mandaron a buscar, a llamar y me iban hacer una cirugía… **[sonido de queja]** y en mi cara “no me dijo yo hago cirugía señora, le hago cualquier cirugía”

**I:** ya, ahora sí. Muchísimas gracias, que les vaya muy bien.

**HEALTH CENTER 3- PATIENTS**

**Investigador (I):** ¿Qué pasó el día en que le dijeron que tenían diabetes ¿Qué pasó con ustedes? ¿Qué pensaron como vieron esto para adelante? ¿Quién parte?

**Participante (P):** Bueno a mí… eh… una prima mía tenía un… tiene una amiga que es doctora y se especializa en… es diabetóloga, un día me dijo, Isabel se llama, “Anda a la consulta” de ahí me hizo algunos exámenes, me tomó la glicemia, y me dijo, mira, “Así como tú vas...vas, vas, con derechito a la diabetes” y yo como, bueno esto hartos años atrás, “ah dije yo, seguí no más po” hasta que me dio la diabetes y me comunicaron que tenía diabetes, y bueno yo dije “estoy consciente que la diabetes es un cáncer silencioso, que ataca a los órganos, la vista a algunas personas les ataca más a la vista, a otros les ataca otros órganos… ya…” pero uno cuidándose, tomándose los remedios que indican los profesionales, uno se puede mantener, digamos, no comiendo frituras, porque también es llica la comiiiida **[Gente comienza a reírse, ya que le encuentra la razón]** La sopaipillas!! Dicen otros, Y comerse un pernil, unas prietas, unas longanizas, al ladito ahí, aaah que rico**… [Algunos de fondo hablan de comidas parecidas].**

**I:** ¿Eso es lo más complejo? La parte de la comida cuando…

**P:** Pueeee claaaaro, yo creo que a todos nos pasa, porque uno dice de repente, claro, no como diez kilos de pollo, pero sin sal **[se ríe]** entonces eso es lo más problemático es la comida, para mí.

**P:** Para mí no… **[Balbucean unos Entrevistados e interrumpen otros],** que me amputen alguna pierna alguna cosa…

**I: “**Eso es lo que más miedo le da”? y ¿qué ha sido lo complicado de ser diabético o qué cosas han sido, a lo mejor, no tan difícil?

**[Silencio]**

**P:** Porque…Bueno, nunca pensé que iba a tener esta enfermedad y… en parte es porque… era familiar, la mamá, era una herencia que recibió, en vez de recibir plata, recibí enfermedades **[algunos se ríen]** hay que saber no más, hay que cuidarse, sobre todo, porque está en uno, está en uno que se cuide, si uno quiere seguir viviendo, está todo en uno.

**I:** Cuénteme

**P:** Bueno yo, por ejemplo, tengo un hermano que murió, se estaba dializando porque era diabético, esto es hereditario, a mí me descubrió la diabetes hace como quince años atrás o veinte años atrás, ya no me acuerdo**… [Silencio]…** Pero empecé mal yo, porque yo no me cuidaba, paná po, paná po, wen asado, wena comida, wen copete, wenas mujeres **[todos se ríen y ser burlan por lo de las mujeres]** Entonces, entonces las trasnochadas, yo nunca he sido bueno para tomar, ni fumo, ni fumo yo tampoco, pero empezó un día que**…[silencio]…** una persona que me dijo “quiero hacerte las uñas” **...[silencio]…**Gratis. Me las hice po, pero resultó que esta persona, tenía un callito ahí y lo dejó muy delgadito, delgadiiito al dedo chico, ahí quedó bien, “aaah perfecto gracias, quedó weno”, pero a los pocos días, mi mujer me ve y tengo con sangre **[investigador: aaah no cicatrizó, con voz de tristeza**] ahí, tengo con sangre, con mi mujer fuimos al SAPU de aquí de Plaza Garín y el médico me ve, “hay que cortar”, no dejó irme po, ahí me dejó, ambulancia, posta tres, hospital, fuera el dedo **[exclama un participante: “ooohh…”, silencio largo],** perfecto, ya, tuve como dos días en el hospital y de alta. Pero al tiempo después, no tanto tiempo tampoco, empezó a ponerse negro el otro **[Silencio].**

**I:** ¿usted sabía que tenía diabetes?

**P:** Claro

**P:** No, entonces, el otro dedo, a la posta, vine para acá porque acá a mí me… no es por ná… pero a mí me atienden muy bien aquí

**[Participantes interrumpen: “Sí, excelente” “sí” “es muy, muy weno”]**

**P:** La sita Cony, la sita Scarlett, el doctor, todos, pero un siete

**P:** Mejor que una clínica

**P:** Mejor, mucho mejor

**P:** muy buena la tención aquí

**P:** Muy wena

**[Participantes interrumpen: “Excelente” “muy wena” “sí” “mejor que una clínica”]**

**P:** Este consultorio es excelente **[continuando con la historia]** bueno, y hospital otra vez, **[hace sonido como si le cortaran el dedo]** el otro dedo, ya me quedaban tres, después, a los meses después ¡Pan! **[Sonido como si le cortaran otro dedo],** otro más… **[silencio…]**

**P:** Sí, vamos… y el doctor me dice, sabí cuántas palabras textuales de él, “Don Julio Rojas, sabí weón”, me dijo, “te voy a cortarte todos los dedos y se para esta cuestión”**… [silencio]…** Doctor, le dije, yo toy con mano, le preguntaron a mi señora, mi señora, así, se para, ya… me cortó los cinco, ahí quedé choco **[Silencio]** Estuve un tiempo bien **[Silencio]** y un día mi nieto, estaba jugando por Magallanes y me dice “¿tata, vamos a la cancha?” “vamos po hijo” “fuimos a la cancha a Maipú…Cerillos, a mí me gusta gritarlo al lado, alentarlo ahí, y empezar a correr, andar por la orilla de la cancha **[Silencio],** le gritaba…

**P:** Quiero entender un poco el manejo, el manejo del tratamiento, ¿qué pasa con los tratamientos?

**P:** Si uno se los deja de tomar, ahí es cuando empiezan los problemas **[Interrumpen algunos: “claro”]**

**P:** Es que eso ya es problema de uno, aquí no corre, la enfermera, ni el médico, aquí corre la mujer de uno o la familia de uno.

**[Comienzan a hablar al mismo tiempo] La alimentación creo también… correcto**

**I:** ¿y el que no tiene familia y tiene que hacerse cargo…?

**[Comienzan a hablar al mismo tiempo]**

**P:** Pero correcto. Claro que es más complejo.

**P: [Esposa del entrevistado]:** Para una persona sola, yo creo que sí.

**P:** Déjeme terminar mi historia porque es un poco trágica la cosa… [Un participante habla de fono: “pero como conclusión si po la cosa”], bueno la conclusión **…[silencio]…** llego a la casa y me acuesto un rato y no me saco las zapatillas **…[Silencio]… [interrumpe un entrevistado: “Sabe cuál es su problema, usted tiene que quererse un poco”]** Correcto, correcto, allá vamos. **[Interrumpe un entrevistado: autoestima se llama eso] [Silencio]** Y me mujer me mira y me dice “¡Sácate los zapatos!” y yo “¡noooo… así!”, macho po, claro ¡quee!, empecé con esta mano, una ampolla, una ampolla, vine para acá al consultorio, estaba la señorita Scarlet en ese tiempo, estaba de enfermera acá, ¡Me curó po!, me curó, pero qué es lo que pasó que la infección se fue para al lado, mire po.

**P: [Esposa del entrevistado]:** La conclusión, se lo digo yo al final quedo sin ningún dedo, no tiene dedos en ninguno de los dos pies

**P:** al final **[hace un gesto de que le cortaron todos los dedos]**

**P: [Esposa del entrevistado]:** No tiene ningún dedo.

**P:** ¿Y desde ahí ha podido manejar mejor? ¿Hace cuánto tiempo fue eso?

**P: [Esposa del entrevistado]:** llevamos seis años en total.

**P:** Seis años Ya… ya…

**P. [Esposa del entrevistado 3]**: llevamos seis años.

**P:** y con la diabetes me enfermé de… al final…de próstata.

**I:** o sea, Yo sé que en general no tienen una sola enfermedad, por lo tanto, van a tener tratamientos para varias cosas, en general, eso complica la vida diaria, ¿complica con el trabajo?, ¿cómo se puede lidiar cuando uno tiene que tomar pastillas? ¿Cómo se hace?

**P:** Emm… bueno… No es problema, sino es que uno, como dijo aquí el caballero acá, quererse, quererse un poco más, ya, es dejar las cosas que uno hace mal, ya, porque estamos conscientes… yo también siendo diabético, me pasó algo parecido a lo suyo, ya, que fue en invierno, como tres años atrás, no como… sí, como tres años atrás, me mojé los zapatos, yo vivo con una tía, y me dijo, “oye, cámbiate el zapato” “¡No, si…!” y me veo también el dedo del pies, ya, y me veo que estaba como eeeh…. Supurando, ya, y le digo a mi tía, “sabí, vamos a ir al consultorio”, “ya”, me vio mi tía y me dijo “ya, vamos altiro”, llegamos acá y me ve Don Esteban, ya, y él me vio y me dijo, “ooohhh Don Manuel”. Ya, me curó, “lo voy a mandar al hospital”, me dijo, “porque lo van a cortar ese dedo”, ya bueno, yo dije “un dedo, me quedan cuatro”. Sucede que cuando estaba en pabellón, ese mismo día me operaron, cuando estoy en pabellón, se me acerca la doctora González y la doctora Meza, y me dicen, “oye, Manuel, sabes, tenemos una mala noticia”, “¿si, doctora?”, “yo sé que, por dentro, están todos los dedos infectados, menos, el dedo, guatoncito, me dijo”, “aaaah dije yo, me deja el guatoncito pa afirmarme, pa tener apoyo”.

**I:** Yo entiendo, que yo sé que lo más traumático de la diabetes es el tema de las amputaciones, pero” me gustaría entender, ¿qué es fácil y difícil es esta realidad y llevar el día a día?, ¿qué le hace fácil y qué le hace difícil?,

**P**: Es difícil, es difícil,

**I:** ¿qué es lo más difícil?

**P**: Es que uno se tienta, y a veces, a uno lo invitan a un cumpleaños o a algo, y hay cosas ricas pa comer y uno se tienta, eso para mí… es lo más complejo

**I:** La comida es lo más complejo,

**P:** Es lo más complejo para mi

**I:** y el resto, ¿qué dice? ¿Qué es lo más complejo de la diabetes?, para poder cuidarse, ¿qué es lo más difícil? En la vida diaria, en la casa, de todas las cosas que le dicen que haga qué es lo más difícil.

**P:** Tantas cosas

**I:** ¿cómo qué? deme ejemplos

**P**: Hace lo que más le gusta

**I:** ¿qué es lo que más le gusta?

**P**: yo perdí la visión ya, perdí la visión… pero me regalaron un perro y tiene motivación igual, motivarse po, la diabetes, la diabetes uno la lleva en la sangre, nos tocó a nosotros, tenemos que asumir no más po.

**P**: yo creo [**un participante tose mucho, produce interrupción en la conversación]** porque hay cosas peores

**P:** ¡¡Imagínese!! Yo era relojero y perdí la visión **[exclama un participante ¡uuuhhh!]** ahora estoy incapacitado de trabajar, a penas la veo los ojos azules

**P**: Cuando uno es notificado de la diabetes, la mayoría de las veces, los trabajadores, no le seguimos los consejos adecuados, por cumplir la meta que siempre fijan antes, eeeh… como para cumplir nuestra misión de trabajadores. Y a mí en un principio me descubrieron cuando tenía 45 años, ahora a los 74 años, en el año 2010 tuvieron que amputarme una pierna y en el año 2013 he estado tres años y no he podido currar bien esta herida **[un participante interrumpe: su cicatrización no es buena]** no, es muy lenta.

**I:**¿no podía cuidarse mientras cumplía en el trabajo?

**P:** Es que yo cumplía con la misión de la pastilla, pero no es solamente la pastilla, parece que tiene que venir otro adecuado…

**I:** ¿Pero las pastillas se las tomaba todas? ¿aún así?

**P:** Aun así, se mantenía alta el azúcar.

**I:** ¿Y qué pasa con la actividad física?

**P:** No ser sedentario, no ser sedentario

**P:** En el caso mío yo he sido deportista toda la vida y me mantenía jugando, juego básquetbol y futbol, pero después, como decía el caballero, viene el asado después del partido, pero fíjese que los médicos autorizaban que con cuidado se saliera de la dieta el sábado, el domingo si había un cumpleaños.

**I:** Ya, o sea, ¿eso se lo autorizaban?

**P:** Sí, pero con control, pero las pastillas no cumplen la misión de… de… ¿cómo se llama?... de mantener el nivel de azúcar en la sangre.

**P:** Mejora en algo, pero no cumplen todo.

**I:** O sea, en general lo que más complica es la comida, ¿Qué me cuenta usted, la única dama del grupo?

**P:** Es que los exámenes que he tenido este año de rutina han estado bien, pero a mí me preocupa eso que los escucho a ustedes sus testimonios, de que ya se les presentó algo, pero es porque la persona, me gustaría saber ¿si es porque la persona ya estaba con el diagnóstico de diabetes o es porque se le presentó eso? Y recién vienen a saber …

**[Interrumpen dos entrevistados y hablan intercalados]**

**P:** Eso es hereditario es hereditario

**P:** En mi familia por lo menos no hay diabéticos, de repente es la buena mesa, según dicen. También que como que...

**P**: La cirrosis también va por la buena mesa.

**P:** Por ejemplo, Mi padre era en esos años, no era famosa la diabetes.

**P:** Claro, como que uno la miraba como una simple enfermedad. Ahora con los años…

**[Todos emiten comentarios y hablan al mismo tiempo]**

**P:** No había tanto**,** Ahora hasta los niños, nacen con diabetes, niños chicos, los mismos hijos de jugadores de futbol, Arturo Vidal.

**P:** Arturo Vidal, gracias a que tiene dinero puede comprar la maquinita…

**[Todos emiten comentarios y hablan al mismo tiempo]**

**P:** ¡¿Pero eso son otros tipos de diabetes la de los niños?!

**I:** Algunos han tenido que colocarse insulina, ¿qué pasa con la insulina? ¿es complejo se puede organizar la vida con la insulina?

**P**: Si

**P:** Me coloco poquito, en la mañana me coloco doce y la noche diez, me he mantenido bien, el otro día, cuando me tomaron la azúcar tenía 130, 120 máximo.

**[Algunos emiten comentarios al mismo tiempo]**

**P:** Yo hace más o menos como veinticinco años que tengo diabetes, gracias a Dios tengo todos mis dedos, nunca he tenido una herida, ahora tuve una herida porque me gusta mucho andar, a pie pelado, entonces me dijeron que no, pero ya la herida está cerrando… ya creo que hoy día me van a dar de alta. Lo que pasa es que yo en mi vida que fui tomador, un día conocí al Señor, a lo mejor no viene al caso **[Algunos interrumpen: “está bien, está bien”],** yo vendo de una familia cristiana, mis abuelos, Y Dios me salió al camino un día, yo nunca pensé humanamente estar en una iglesia alabando a Dios, pero ya llevo 25 años alabando a Dios. Yo como de todo, porque Dios me sanó hasta de un cáncer a mí, entonces, mi fe creció, Dios me habló, porque Dios todavía habla por intermedio sus profetas, porque todavía existen profetas, aunque para el ser humano tiene una gran cualidad que es muy incrédulo el ser humano, eso es lo malo del ser humano. Y Dios me dijo que me iba a sanar de la diabetes, no por eso no me cuido, igual me cuido yo, comprende, pero no así rígido!!!, siempre unjo mis alimentos, unjo todas mis cositas señor, ya llegar el día que usted me va a sanar, para yo contar mi testimonio, que Dios me salvó de la diabetes, mi vida sigo, al principio, cuando me diagnosticaron, no crean, me cayó como un balde de agua fría!, pero después Dios habló a mi corazón, y me dijo, “no te preocupís, yo estoy contigo”, y como de ahí pun se fue, y he vivido mi vida normal, claro, yo no tomo, no fumo, nada,

**P:** Igual me cuido, me pongo mi insulina, pero igual me cuido, de repente me como mi longaniza **[se ríe].** Las cosas buenas como siempre como todo ser humano

**[Todos emiten comentarios y hablan al mismo tiempo]**

**P**: Llevo 25 años gracias a Dios estoy bien y **s**é que voy a terminar bien.

**I:** Perfecto. Ud. ¿Nos iba a contar algo? También ¿Está con insulina?

**P:** Me estoy inyectando a las 9 de la noche, 10 unidades, llevo cerca de veinte años.

**I:** ¿Y qué ha sido lo más complejo? ¿Lo más difícil?

**P:** O sea, lo más complejo es cuando supe y llegué a la posta con 540 de glicemia.

**[Todos emiten comentarios “uuuhh” “coma diabético”]**

**P:** Me he cuidado, y me he desvandado, pero no tanto

**[Alguien interrumpe, “diga la verdad, diga la verdad”]**

**P:** Pero, hasta el momento **[habla pausadamente]** he estado bien, pero con control médico en el hospital todos los meses.

**[Todos emiten comentarios, algunos hacen diálogos aparte: “Lo que yo digo la comida…” “Mucho aliño en las comidas”]**

**I:** Bien, cuénteme

**P:** Yo encontré como controlar mi diabetes

**P:** Yo me estoy… tenía que ponerme insulina tres veces al día, pero recibí de una persona… una hierba que se llama hierba peruana, Alcampuri, ya ahora me estoy controlando con eso, pero sí, yo me hago una glicemia todos los días, con la máquina y se gasta, pero que a veces yo tenía 400, 500, y ahora, 85, 90, 100, 110 no más.

**[Algunos participantes exclaman: “tsss… está bien” “Tá weno” “¡Excelente!” “Hierba Alcampuri”]**

**I:** Y ¿la alimentación?

**P:** ¡Como de todo!, no tanto como antes, me comía dos sándwiches de arrollado o me comía un pernil entero con papas cocidas, ahora, ahora comprendo que la fritura es lo más peligroso para la diabetes, porque yo me como dos o tres empanadas y al rato me controlo **[un participante exclama: “ah no ahí sí que no”]** se me sube de 80 a 300… entonces, ¿qué hago? Me tomo mi hierba en la noche que es amarga, no puede ser más amarga y en la mañana la glicemia… 95, 100…

**I:** De ahí le piden el dato al caballero

**P**: Pero siempre trate de tener su maquinita y también aprenda hacérsela usted solito.

**P:** Usted, usted es solito, yo tengo quien me mande, ella es la que manda, ella es…

**[Todos emiten comentario, hablan al mismo tiempo y se forman grupos de conversación diferentes]**

**I:** ¿algún tipo?, de los que pueden….

**[Los participantes empiezan a preguntar por la hierba peruana, dónde se compra, cuánto vale, tamaño de la bolsa, etc. Los participantes le dan mucha importancia por saber de la hierba y pasan varios minutos hablando de eso. “La compran en la vega, cuesta en 500 pesos la bolsa. Se toma un vasito, se toma una vez al día. Hace orinar arto…como loco”]**

**I:** Oigan, Orina harto dice el caballero ¿qué más…?

**[Siguen hablando de la hierba. “La venden en la feria …”]**

**P:** Yo ya llevo 7 años, porque yo ya perdí la visión.

**P:** Cincuenta y siempre…

**P:** ¿Es más peligroso…? El…. ¿La… la falta de azúcar que… a veces, uno puede tener 300, 400, y no es taaan peligroso… como le baja a 60? ¿Se puede morir en ese mismo momento?

**P:** Lo que nos conversaron el otro día igual

**P:** Los dos polos pueden ser peligrosos 80, 90 100…

**I:** Pero de las indicaciones…

**[hablan al mismo tiempo sobre qué es peor]**

**I:** Oigan, ¿pero los han educado?

**[Hablan al mismo tiempo: “no”, “yo sí”, “sí”]**

**I:** usted, usted quiere decir algo.

**P.** Bueno, yo soy diabético, hace varios años

**P**: Hace siete años.

**P:** Resulta que yo me he operado, me he hecho operaciones bastantes graves, me operaron del riñón, y me preguntaron si yo tenía diabetes, y yo sí declara, y me dijo no me dijo, no está declarada, me dijo…. **[Interrumpe un participante y se descontinua la conversación]…**

**[SE VA UN PARTICIPANTE Y SE DESPIDE: “señorita me tengo que ir a la pega, un gusto haberla conocido…”]**

**P:** Bueno me dijo…. Pasa lo siguiente… me operó y se llevó una sorpresa me dijo por lo menos unos meses ... ya estaba cicatrizada y me volvió nuevamente y estaba todo sano….

**P:** Los organismos son diferentes, yo tengo sangre azul.

**P:** Señorita, hace poco que me operaron de la espalda por el calor, esta cosa que sale en la, como se llama… lunar… entonces se me agrandó así de grande. También tenía todo preparado y el doctor me dijo, “tú tienes diabetes” Un poco fregado… **[habla muy despacio el participante no se distingue lo que cuenta]. N**o te vas a morir parece…

**I: ¿**Cómo ha podido controlar?

**P:** Tal como me lo han dicho

**I:** ¿Qué ha sido lo más complejo?

**P:** Bien. Bien, ahora estoy tomando una hierba que se llama… pata de… [**silencio para acordarse]**… viene tres partes, en cápsulas, en gotas y en… cuánto se llama…

**[Intervienen participantes dando ideas de qué puede ser el nombre. “La de los mapuches dicen...”]**

**P:** No sé … pero el hecho…

**I:** Hay confianza en estos métodos alternativos ¿ah?

**[Hablan al mismo tiempo]**

**P**: ¿Cómo se llama?… **[Le preguntan al investigador]…**

**I:** Gladiola, así se llama.

**P**: Dicen que es buena hasta pa cáncer

**[Comienzan a hablar al mismo tiempo de las hierbas]**

**P**: Señorita, Nosotros los diabéticos… ¿Podemos tomar mate?

**P:** Que yo sepa no mucho.

**[Comienzan a hablar al mismo tiempo sobre la hierba mate]**

**P**: ¿nosotros como diabéticos podemos tomar un vasito de vino en el almuerzo?

**I:** Usted se lo debe preguntar al médico que lo controla.

**[Un participante dice: “Sí, el médico dice que sí”]**

**P**: Yo creo que el señor, muy buena pregunta porque a mí me han dicho que el médico, las enfermeras, que puedo tomar un poquito en el almuerzo.

**[¡No la botella!, exclama uno]**

**P**: Noooo, si uno no es vicioso… un vasito.

**P**: A mí también me dijeron lo mismo, pero yo me avivé …miren la copa que tengo.

**[Comienzan a bromear respecto a cuánto toman los integrantes que es una copa, pero una copa grande, por ejemplo]**

**P**: Dra. Yo creo que aquí todo lo que hemos sacado de conclusión es que no tenemos una información desde un principio de nuestra enfermedad y eso radica que no la cuidemos, nos descuidemos por factores de trabajo, otras veces, por llegar atrasado y nos vamos descolocando por los horarios de nuestro cuerpo como para colocarnos, ya sea inyección, ya sea medicamento.

**P**: O uno no le toma el valor a la enfermedad.

**[Comienzan a hablar al mismo tiempo]**

**P:** Yo creo que hay información, pero resulta que uno no la toma en cuenta, como debe ser.

**I:** ¿Por qué no la toman en ceanta?

**P:** Me van a contar las patas.

**P:** Doctora ahí en la parte sexual… **[Hablan al mismo tiempo].**

**P:** Hay impotencia sexual…

**I:** El caballero quería decir algo…

**P:** Es que nosotros tenemos el ego demasiado alto y piensa para el interno, qué me hará si me salto esto… me lo pongo más tarde y después no se repone más tarde ni el medicamento, ni el horario de la esta, y ahí viene el descuido mayor.

**I:** ¿invencible?

**P:** No tanto invencible, pero que, que me va a pasar a mí si un poquito no más, un ratito o no anda con los medicamentos para desplazarse, llevarlos a la mano.

**I:** ¿y cuando uno piensa en la familia?

**P:** Fíjese, que no, acá yo como de todo… pernil, arrollado, longaniza, antes no, porque antes yo el lunes partía, me compraba una cabeza de chancho en Matucana y mi mujer la cocía, la colocaba en la “once” en la mesa y yo pa… **[Hace sonidos refiriéndose que se lo come rápidamente]…** con un pebre y su ajicito.

**P:** ¿Y su peso es normal?

**P:** No, he subido.

**P:** Yo pesaba 130 kilos.

**P:** Yo igual pesaba 130, pero ahora he bajado, yo la última vez que me pesó un médico eran ciento

**P: ¿**Para su estatura cuanto debería pesar?

**P:** yo debería pesar 90, por la estatura.

**P:** Y hoy día cómo estamos con la cabeza de chancho.

**P:** Bueno ¡Rico po! Entonces, ahora, ahora yo ahora en mi casa yo no le como torta.

**I:** ¿y el resto?

**P:** No es porque quiera alabar, pero… es mejor que una enfermera po oiga, es pesada, exigente, me tiene cortito po oiga.

**I:** ¿y el resto? ¿la familia?

**[Se ponen a responder al mismo tiempo algunos participantes]**

**P:** Yo vine a control… **[no se le entiende lo que dice**]… entonces, viene y me dice, “tienes que ser fuerte con lo que voy a decir, tienes un cáncer avanzado”, fui a un traumatológico, sabe me dijo, que “tuvo con principio hace muchos años atrás, porque un doctor no puede darle un diagnóstico sin hacerle los exámenes, ese es el grave error”, “sabe usted doctor, que usted se equivocó”, llevaba tres semanas haciéndome exámenes allá y acá, “cómo va hacer posible”, y me dijo “¿qué tanto si te vai a morir igual?”… **[Entrevistado habla muy despacio y lento]**

**P:** Shuuuu la ética que tiene el Dr. ¿Ha? **[Entrevistados emiten comentarios negativos respecto a la respuesta del médico]**

**P:** Oiga ¿Cómo me voy a morir igual? Dijo…

**P: “hay médicos y médicos”**

**P: Por eso hay que tener una segunda opinión.**

**P:** El hecho ¿qué pasa si no voy a hacerme los exámenes y reviso todo? Iba donde el cardiólogo, estai bien, iba donde el urólogo, también, me dijo el médico, ándate, me dijo al Dr. El hijo del “Carcuro”; me dijo yo te voy a mandar aquí…. No hay nada…

**I:** Al final……….

**P:** No había nada, que no tenía nada, me dio de alta, no tenía nada, bueno la diabetes declarada…. entonces, qué pasa, si el doctor se equivocó, se equivocó no más, pero si uno se equivoca…

**P:** Es que ellos siempre las ganan.

**P**: A veces tienen eso de decir no más las cosas.

**P:** Es que ellos siempre las ganan, por ejemplo, a mí se me infectó la herida estando en el hospital, ya, el médico ordenó hacer un aseo quirúrgico… perfecto po, perfecto, ya que estamos en el hospital aprovechamos… estoy aun paso del pabellón ahí… a un paso, llega el médico como a la una de la mañana, “¿qué le pasa?”, “no, doctor un aseo quirúrgico” **[Silencio]…** no habla napo… y le hace al otro médico…. **[sonido de corte]** y me mujer y mis hijas me habían advertido, le van a pinchar la raquídea y le van a cortar el pie, ya perfecto, doctor le digo yo, ¿qué me va a hacer? Se da media vuelta y se va, y el otro médico, dice, “viejo, te van a cotar el pie de la canilla pa arriba… pa abajo” y las enfermeras, las que estaban ahí, me dicen Don Urse, “llame a su familia y váyase le van a cortar los pies estos sanguinarios” **[participantes: ¡es un carnicero!]** ¡Un carnicero po oiga! Guillermo Castro se llama el famoso médico, está en Serena en estos momentos.

**P**: ¡lo único que saben es amputar!

**P:** Sale más barato amputar antes que curar

**P:** Antes de seguir un tratamiento

**P:** Yo les dije ahí a las chiquillas “por favor llamen a mi casa”, Una fulanita que se llama enfermera… Rocío se llamaba… igual que mi hija, correcto, llama pa mi casa, y no estaban en pie… y les grito yo, con todo respeto a ustedes, ¡¡¡¡Vieja, ven a buscarme que este culiao me va a cortar la pata!!!! Yo estaba desnudo ahí esperando. Mire, por lo menos, yo vivo cerca del hospital, se demoraron dos minutos en llegar y no querían dejar entrar… **[Grita un participante: ¡Lo salvaron**!]… no querían dejarla entrar, llegan mis hijas al hospital, y en el auto siempre andan trayendo un chal, “y qué te creí te acercai pa acá, te pego un combo en el hocico yo al médico” Don Guillermo Castro se llama el famoso médico, que ahora está en Serena. Me vine po, y acá estoy con mi pie.

**P:** ¿Cómo pueden existir esas cosas así?

**[Comentan al mismo tiempo horrorizados por los médicos que actúan así]**

**[“No, no confío” hablan al mismo tiempo]**

**P:** ¿Qué hace que uno no confíe?

**P:** Yo, yo, yo estuve un año esperando una hora en el San Juan de Dios, y llegó un médico joven y me dice, “Usted va a quedar ciego”, hace años 10 que estoy con la… aún me queda uno…

**I:** ¿qué hace que uno confíe o no confíe? ¿Qué hace que uno desconfíe en los médicos hoy en día?

**P:** No**,** Cuando un médico le da la confianza a uno

**I:** ¿cómo se la da? ¿Cómo se logra?

**P**: con su amabilidad…

**P**: Con su disposición

**P:** Con su disposición para conversar, yo por ejemplo….

**I:** Espere, vamos a escuchar a su compañero, también

**P:** yo veo que ahora… los doctores de antes, los más antiguos eran muy buenos, ahora usted ve que ellos están estudiando para ser médicos, a ellos los mandan a un hospital, y ¿qué hacen ellos?, cumplen la hora no más y chao

**I:** Ya, pero ¿qué hace que usted confíe? ¿qué es lo que hace…?

**P:** Señorita míreme, pasa lo siguiente, yo siempre cuando salgo del rancho, me encomiendo a nuestro Señor y tengo un Santo que es muy bueno que se llama San José, hágase tu voluntad, digo yo, y vamos, ahí, no es por nada, pero me han atendido de mil maravillas, entonces, si él es médico y me da el saludo, me da la mano y conversa conmigo, y me dice, ¿qué es lo que trae? Porque el médico no ve la bolita. Hay que decirle lo que le duele, ¿cierto? Y he llegado a una conclusión, que sin un médico no somos nada, porque ahí yo le pongo el caso de doctor Preciado, un excelente señor

**P**: ¡Sí! Muy bueno

**P:** ahora mismo vine a control de... cuánto se llama… de la… alimentación…. **[exclama un participante: diabetógoco]**…. Sí… Me atendió un joven, oiga, excelente, entonces yo pienso, y una vez yo lo consulté “¿Qué vale trabajar aquí un médico que trabajé en un consultorio? ¿Cuál es el valor que tiene usted o el señor de allá?” [**silencio]** “Mira amigo, me dijo, aquí, por ejemplo, tú pagas hasta la respiración, allá tú pagas lo que puedes”, y el médico me dijo, “igual que yo, solamente mandan las máquinas”.

**P:** ¿Me permite? Mire yo creo que también en esto los chilenos no han avanzado en la cultura, de repente hasta le podemos… no con bases científicas, sino que nuestro instinto, discutir a un médico. Antes eso no se permitía…

**I:** estaba diciendo que ahora no se confía igual, ¿por qué?

**[Hay un silencio y después hablan al mismo tiempo]**

**P:** yo, yo perdí la perdí ya hace mucho tiempo, ahora, más todavía, tengo a mi señora en el hospital de San Borja, la operé, ya lleva hace quince días, para que la operaran del colon y le sacaron un tumor que tenía ella ahí, pero resulta que todavía no me la puedo llevar, porque el doctor que la operó, cortó algo más, que se le llenaron los pulmones de líquido, le tuvieron que colocar sondas por abajo, por la espalda para sacarle el líquido y ahí está ya hace dos semanas, mi hijo, fue ayer, mire no le pegó al doctor una patá por la escalera pa abajo porque pensó en su madre no más y porque ya… yo soy re-paciente, aparte que ya estoy viejo ya

**I:** Ya, y en general…

**P:** No hay humanidad, no hay humanidad, son insensibles los todos, totalmente insensibles

**I:** vamos a tener que cerrar ya, …

**P:** Antiguamente se daban que las familias eran médicos, que el hijo tenía que ser médico y que ese hijo…. Ese cabro no quería estudiar medicina, quería ser abogado, entonces, hay muchos profesionales frustrados en diferentes carreras, porque el papá es abogado el hijo tenía que ser abogado, entonces, no hacen su función como corresponde

**I:** Ya…….

**P:** Yo tengo un sistema ahora, pa que no me suba el azúcar, no rabeo con nada, soy hipertenso y no rabeo con nada, “se cayó el termo, se quebró, se quebró chao”, en cambio, lo que pasa que a usted se le cae una cuchara “¡¡¡¡ooohhh, se me cayó la cuchara!!!” ¿qué pasa?, que se empieza a subir la azúcar y todo… No rabeo con nada, no peleo con nada, me pueden insultar, perfecto hasta luego chao, porque si uno se para aquí, entonces, empieza a subir el azúcar y todo

**[Investigador da el cierre al grupo focal y da las gracias]**

**I:** He aprendido mucho hoy, les doy las gracias profundamente, he aprendido un montón y hay muchísimas cosas que podemos rescatar de los testimonios de ustedes, para tratar de mejorar la atención…muchísimas gracias.

**HEALTH CENTER 1- HEALTH PROVIDERS**

**Investigator (I):** […] Les agradezco mucho su participación y firma del CI. Les solicito hablar alto, es bastante sensible la grabadora, pero, de todas maneras. Vamos a comenzar por colocar una situación hipotética en que ustedes tienen que darle el diagnóstico a su paciente. ¿Cómo se da esa situación con el paciente? El que quiera comenzar…

**Proveedor de Salud (PS):** ¿Estamos hablando de cualquier patología crónica o estamos hablando de… diabetes?

**(PS):** Es que hay bastante diferencia, porque cuando a un paciente uno le dice hipertensión, como que se lo toman bien light, pero cuando uno les habla de diabetes se ponen bien peludos. No todos, pero, en términos generales, la diabetes la reciben como algo catastrófico, más catastrófico que la hipertensión.

**(I):** ¿Y lo plantean así?

**(PS):** Dicen varios peros, “pero, pero, pero”, en cambio cuando es hipertensión ni un “pero,” ¿están de acuerdo con lo que digo?

**Varios (PS):** Sí, de todas maneras.

**(PS):** O sea, hay muchos pacientes que uno les pregunta ¿qué enfermedades crónicas tienen? y dicen, como “no nada” y uno revisa y tienen hipertensión, y uno les dice “pero si tiene hipertensión” y dicen “aah sí, sí”, pero eso no pasa con la diabetes, la hipertensión es como nada, la diabetes sí.

**(I):** ¿Por qué creen ustedes que pasa eso?

**(PS):** Yo creo que es porque la gente ve a su alrededor a otros pacientes diabéticos y ve las consecuencias que ha tenido la diabetes en otras personas conocidas y se asusta, pero en cambio no ve las consecuencias de la hipertensión en sus pares, o no las sabe reconocer como consecuencias de hipertensión, pero sí la diabetes sí: que están ciegos, que la diálisis, que les cortan un pie. Entonces se asustan en cuanto a la diabetes, pero no a la hipertensión.

**(PS):** A mí me da la impresión de que sí, que las asocian a complicaciones como decía el doctor y la doctora, mas catastróficas, y, por otro lado, la hipertensión yo siento que ellos igual asocian la enfermedad como un conjunto de síntomas, como soy de presión alta o la presión alta me hace marearme y me duele la cabeza…

**[INTERRUPCIÓN: SE INTEGRAN NUEVOS PARTICIPANTES]**

**(PS):** **[continuación]** Entonces claro, como dice la Maite, no es una relación causal en torno a las complicaciones que enfrentan ellos.

**(I):** Le damos la bienvenida a las personas que se integran.

**(PS):** Bueno, cuando yo hago el diagnóstico de una enfermedad crónica, de una hipertensión o la diabetes… Eehh… comienzo a educar al paciente, decirle que es una enfermedad que no tiene cura, lo único que tiene es control. Yo le hago así, una diferencia “si a usted le dijeran que enfermedad quieres tener el cáncer, la hipertensión o la diabetes ¿usted que elegiría?” “la hipertensión” “¿y por qué no el cáncer?” “porque con el cáncer me voy a morir” “bueno la diabetes y la hipertensión no tiene cura”, “ya, pero si usted no lleva un buen control igual se va a morir, porque se puede descompensar y vienen todas las complicaciones”, bueno y ahí asume y que va a cambiar el estilo de vida, va a comenzar a hacer la dieta y bueno ahí lo mando al nutricionista para que le hable más acerca de la patología.

**(PS):** Negación, lo primero que dice niega que está enfermo.

**(I): ¿**A pesar de los exámenes?

**(PS):** A pesar de eso.

**(PS):** Dudan de todo lo que se les dijo anteriormente, otro médico puedo haberles explicado muy bien mejor que lo que uno hace, les explico el médico y nos dicen “pero doctor me dijeron que yo tenía diabetes”.

**(PS):** Claro.

**(I):** … ¿qué pasa después?

**(PS):** A mí lo que generalmente me ha pasado es que al principio cuando yo empecé a trabajar acá, eeh no diagnosticaba mucha diabetes porque me tocaban como los controles de pacientes ya diabéticos, y me he dado cuenta que eso los paciente que ya son diabéticos cuesta muchos más manejarlos que los que son ingresos, por así decirlo, donde yo les he podido dar como la información sobre su enfermedad y como las pautas de tratamiento, y me ha pasado que con estos pacientes que son ingresos, son pacientes de diagnóstico, al final, es mucho más fácil, porque como se sienten bien en realidad todavía como que tampoco asumen todo el estilo de cambio de vida, no saben cómo lo que se les viene, sobre todo lo que tienen que hacer, todo el esfuerzo… pero, generalmente, siempre ha sido explicado y cuando nos damos un buen tiempo, nos damos como media hora en conversar esas cosas, han andado súper bien y han podido, por lo menos… recién vi a dos o tres que ya estaban haciendo ejercicios, ya estaban como cambiando la dieta, y se vio reflejado en la hemoglobina que yo vi o sea en ese sentido puedo decir que los ingresos son los que menos me han costado por así decirlo compensarlos, más los adultos mayores en general…

**(I):** ¿Por qué podría ser esa diferencia?

**(PS):** Por un tema hábito yo creo, también es como…

**(I):** ¿Sí?

**(PS):** Puede ser, también, que al ingresar hay como un cambio en los síntomas que ellos tienen, entonces, después del tratamiento como que sienten o presentan ciertas mejorías, entonces ahí puede que lo asocien con algo positivo el hecho de estarse recuperando.

**(I): ¿**Y de las indicaciones? Por ejemplo, ¿Qué pasa con los medicamentos?

**(PS):** Yo creo que, en la toma del medicamento, bueno, se quejan harto de la Metformina que produce intolerancias gástricas… Eemm… en esa parte, como en la parte de los hipoglicemiantes orales tienen dificultades con eso, y en la insulina, obviamente, cuando ya estoy pensando en introducir la insulina, también presentan… Como que no quieren, una cosa que da lata tener que estarse administrando eso todos los días, inyectable, y no sé, los medicamentos yo creo que en general no hay problemas que se lo tomen, si es que ellos no sienten nada, no se sienten mal con los medicamentos.

**(PS):** No hay muchas opciones, tampoco, porque hay poco, tenemos dos hipoglicemiantes orales en APS y más la insulina, y sería.

**(PS):** Yo creo que sí hay hartas… como que se oculta harto la falta de adherencia al tratamiento, y de hecho uno lo ve cuando uno quiere pasar al paso de la insulina y ve, no sé… hemoglobinas que te indican el inicio de la insulina… pero como uno sabe, o sea, como lo ve ahí, le explica al paciente que vamos a tener que iniciar insulina y de un momento a otro ha mejorado un mucho… la…. Después de tres meses le ha mejorado mucho la hemoglobina glicosilada. En ese sentido, yo creo que tanta mejoría no se debía tan solo por dieta y ejercicio. De hecho lo que… no alteraría tanto la hemoglobina glucosada, sería como sobre estimar eso, yo creo que hay harta falta de adherencia al tratamiento y eso es mucho, depende mucho de la educación que se le da al ingreso del paciente, porque al final en muchos pacientes que no saben los medicamentos que están tomando, no saben ni siquiera para qué sirve cada uno o ni siquiera saben… como que van variando como colores, marca… eh… ya tienen como un popurrí de medicamentos y lo hacen todo al lote y uno les pregunta derechamente, y dicen “no, me los estoy tomando todos súper bien, todo estupendo”.

**(PS):** La respuesta que me dan a mí…. Porque yo siento igual eso, que falta mucha adherencia al tratamiento farmacológico, y la respuesta que me dan, porque son muchos medicamentos, porque…. de repente no saben cuál tomar, se olvidan, no tienen, no sé po, yo les digo “ya, pero coloquen la alarma, entonces, esas respuestas me han dado cuando les pregunto, en general, ¿por qué no lo están tomando?

**[INTERRUPCIÓN DE CELULAR]**

**(I):** ¿interfiere en la vida de los pacientes el número de medicamentos?

**(PS):** **(Todos):** Yo creo que sí, yo creo que sí

**[INTERVENCIONES SUCESIVAS]**

**(PS):** Se sienten invadidos de tantos medicamentos.

**(PS):** Yo, desde farmacia, les puedo decir lo mismo que dice Vásquez, se aburren de tomar tantos medicamentos, se confunden, a veces no tienen claras las dosis….

**[INTERVENCIONES SUCESIVAS]**

**(PS):** Exacto.

**(PS):** Los confunden también lo mismo los colores, de repente llegan de un laboratorio llegan con la pastilla de un cierto color, y después, al otro mes cambia o cambia el tamaño de la pastilla, entonces, ahora hay que pensar que hay pacientes que es diabético, y hay pacientes que ya están con poca visión, entonces, les cuesta leer lo que dice el blíster o el sobre que entregamos los medicamentos nosotros, o sea, yo creo que es súper importante a esos paciente hacerles un seguimiento a largo plazo.

**(PS):** Sí, y lo otro es que también, es que emm… ese que emm, no sepan pa’ qué es, no sé po, algunos no se tomaban la Hidroclorotiazida porque no sabían pa’ qué era.

**(PS):** Entonces, claro, o pacientes que están claramente deprimidos, también, y no les importa tampoco tomarse los medicamentos.

**(PS):** Ocasionalmente, dejan de tomarlos por los motivos que sean y hay como una negación del sistema de apoyo.

**(I):** Ya…

**(PS):** De repente me preguntan, la nutricionista no me explicó esto, “es que el nutricionista puro habla no más po” y el doctor no es una receta… y me consta que hay médicos aquí que explican, no digamos [se arregla la garganta] que discursos floridos, pero explican que el remedio es para esto, hace esto y esto otro, y el paciente no se los toma. De hecho, si uno los apura un poquito, se empieza a encontrar con un montón de pacientes “yo no hago la dieta, doctor”, “pero usted está viviendo una vez a la semana pa’ que le pongan suero”, “¡ah! pero es que uno se acostumbra”. No son muchos, pero esa respuesta me la han dado, pero de los que no siguen la dieta hay un montón, y hay harto más de los que uno quiere, “¡ay! es que yo no hago ninguna dieta pos doctor”.

**(I):** ¿Por qué creen ustedes que pasa eso?

**[INTERRUPCIONES ENTRE DOS ENTREVISTADOS]**

**(PS):** Lo que yo pienso, uno, sin pacientes que son derivados por médicos, generalmente. No vienen voluntariamente al nutricionista, no vienen porque quieren realmente bajar de peso, porque han tomado consciencia de su enfermedad, no vienen por eso, sino que vienen obligados…

**(PS):** No quieren ir.

**(PS):** ¿Pero para qué lo mandan al nutricionista? No quieren ir, entonces ya vienen, ya vienen con ese, con ese muro con respecto a la alimentación.

**(PS):** Claro, obviamente nosotros tenemos que empezar ahí a trabajar con ellos, o sea pss, o sea, son, [Tartamudea] yo… es que… te digo son poco los pacientes que uno en la primera consulta puede eehh trabajar bien con ellos, entonces… y lo otro que nos complica a nosotros que vemos pacientes que… no hacemos una buena… un buen seguimiento, no podemos ver, a lo mejor, mensualmente, sino que lo vemos dos o tres veces en el año, a lo mejor lo ve mi colega y después lo veo yo, entonces, tampoco es el mismo nutricionista que los ve o en extensión o los ve otra colega, entonces, eso, eso influye mucho, obviamente, no podemos tener un solo paciente y ese paciente que nos vea todo el año.

**(PS):** Otro es la falta de apoyo familiar, uno ve en una consulta y les pide que la próxima vez vengan con una persona del grupo familiar para poder explicarles las mismas cosas, y uno consulta, tras consulta, “y bueno, ¿no vino su esposa?” “no, es que no pudo venir, no quiso venir”, eso también… falta mucha ayuda, porque en la casa compran bebida y compran todo y tiene que tomar todo.

**(PS):** Y lo otro es que al final uno no solamente, porque uno si sólo se enfocara en la parte nutricionista, perfecto, pero al final, tenemos que ver los fármacos, la persona con depresión que nos habla de toda su cosa familiar, entonces, al final, la parte nutricional nos queda así, lo citamos lo más pronto posible y la señora después no viene, entonces, eso pasa mucho también.

**(PS):** Claro, como que entre que la convencí de que se la tomara los medicamentos en esos horarios, ya, pasaron 15 minutos y me queda 5 minutos para reforzar alimentación.

**(PS):** Claro, exacto.

**(PS):** Entonces, llego a la alimentación y me doy cuenta de que tiene el despelote en los horarios, también, que la insulina se la inyecta a cualquier hora, desayuna dos horas después, o sea, al final estoy cuarenta minutos o cincuenta minutos con esa persona y emm… después llega el otro, al control del otro mes, y vuelve a hacer lo mismo como que… y no.

**[INTERRUPCIÓN DE DOS ENTREVISTADOS: “Nadie dijo nada”]**

**(PS):** **[continuación]** Nadie le dijo nada…

**(PS):** Como que si salieran de la puerta y se olvidaran de todo lo que uno le dijo, o sea, podrían haber salido del doctor y el doctor le explica los medicamentos y todo, “no es que yo no tenía ni idea, nadie me dijo nada”.

**(PS):** También, me he encontrado con la sorpresa que me dicen… después le explico un poquito las consecuencias y una de las cosas que me dicen o la parte de alimentación me dicen “¡Qué raro doctor! Quiere decir que la nutricionista siempre me ha dicho eso y nunca me había dado cuenta” [RISA GENERALIZADA] Como que…

**[Todos emiten comentarios y ríen sobre la última aseveración del PS]**

**(I):** ¿Es complicado seguir las indicaciones?

**[Algunos al mismo tiempo afirman: “Complicadísimo”, “complicado”]**

**(PS):** Influye horarios a los que ellos no están acostumbrados, además, horarios de toda la familia, también, como dice Don Juan, que influye mucho el apoyo familiar. Además, si es que trabajan, sino muchos acá tienen trabajos por turno, entonces trabajan en turnos de noche, en el Transantiago, que esos turnos son horribles, entonces, influye todo, en realidad se levantan tarde, también, se levantan a las doce del día, almuerzan a las cuatro de la tarde, entonces, convencerlos en hacer ese cambio de hábito, ellos piensan que se tienen que levantar, por lo menos, a las diez de la mañana para almorzar a las una, es un cambio que si no lo han hecho toda la vida, entonces, es complicado.

**(PS):** Y mientras no se sientan mal… no lo hacen… porque ¿para qué me voy a levantar a las diez si no me siento mal? ¿Qué influye si almuerzo a las una o a las cuatro? Si me inyecto la insulina a las diez de la mañana y almuerzo a las una… ¿Para qué voy a tomar desayuno si no me siento mal?”

**(PS):** Yo les digo que tienen que cambiar el chip, la mentalidad, que si ya eres diabético o eres hipertenso, si es hipertenso ya tienes que dejar de ponerle la sal, si quieres comerte una ensalada, comérsela de forma natural, no le pongas sal, si es diabético, ya le hablo a cerca de los hidratos de carbono, que todas las cosas se transforman en azúcar y para usted la bebida es veneno, usted ya no va a tomar bebida, pero lastimosamente aquí en casi todos los lugares una mesa el pan no hace falta, la bebida, Coca-Cola es lo primero que está ahí, yo me he dado cuenta porque antes salía a visitar a los pacientes a terreno. Me he dado cuenta, si me ofrecen algo, un vaso de bebida, Coca-Cola, ya eso es cultura.

**(I):** ¿Debería cambiar su actitud cuando aparecen síntomas?

**(PS):** No necesariamente.

**[RISAS GENERALIZADAS]**

**(PS):** En algunos.

**(PS):** Sí, algunos.

**(PS):** Cuando aparecen muchos los síntomas se cuidan ese período en que vuelven a sentirse bien, uno nota que dejó el azúcar, dejó la bebida, pero al tiempo después “¿y el azúcar?” “no, si la azúcar la dejé” y después uno le vuelve a preguntar y vuelve a estar en un estado de consumir azúcar, el período que están asustados ahí sigue todas las indicaciones.

**(I):** ¿Qué pasa con las indicaciones de actividad física?

**(PS):** Es complejo la actividad física acá en La Pintana por las balaceras, o sea, es una zona que muy vulnerable, no pueden salir, o sea, [Interrumpen varios] o sea, claro, no se puede, lo único que podemos indicarles que hagan dentro de la casa, los niños, los adultos, porque no pueden salir.

**(PS):** A mí, generalmente, la indicación de la actividad física me ha ido bien cuando van en pareja y les sugiero, no sé, actividad de pareja, hacer zumba en videos en YouTube, esas cosas, me ha funcionado ESO, pero ESO no más [con voz denotando lo difícil que es este ámbito y que son excepciones que se cumplen]

**(PS):** Claro, exacto, nosotros igual, en la casa bailar, ¿cachai?

**[COMENTARIOS GENERALIZADOS SOBRE ACTIVIDADES DENTRO DE LA CASA]**

**(PS):** Se trata, al menos para ellos, caminar, eso es lo único que tienen de actividad física.

**(I):** ¿Y el trabajo?

**[INTERRUPCIONES MÚLTIPLES]**

**(PS):** Y el trabajo, “Toda mi jornada laboral camino”, entonces, hay que empezar de nuevo que eso no es actividad física, que tienen que hacer algo adicional, tres veces a la semana, entonces, en esa parte hay que cambiar la información que ellos tienen.

**(PS):** “¡Eso es actividad física, doctor!, yo doctor estoy todo el día moviéndome en la casa, todo el día”.

**(PS):** Y esa respuesta es de todos…

**[TODOS LOS ENTREVISTADOS ASIENTEN ANTE EL COMENTARIO ANTERIOR]**

**(PS):** “Yo me tomo todo, todo, todo, los medicamentos”, “Yo sigo todo, todos, las dietas”

**(PS):** Ahora también lo justifican con las mismas complicaciones, por ejemplo, ya son obesos y tienen artrosis de rodilla, “yo no puedo hacer ejercicio, porque tengo artrosis”, entonces, ahí ya, o “oh no ya me amputaron una pierna, ya no puedo hacer nada”. Así no se le ocurre otra opción de actividad física. Igual tampoco existen los recursos para algo más elaborado, como con natación estamos al otro lado.

**(I):** ¿Hay posibilidades de negociar con ellos?

**(PS):** Uno lo tiene que adaptar, al final cabo uno lo tiene que hacer si uno se restringe como al horario, por ejemplo, uno tiene que modificar su horario de trabajo a los horarios que ellos tienen, o sea, sí, si uno es dueña de casa y uno se levanta a las doce no le voy a decir que almuerce a las cuatro, por ejemplo, pero si es una persona que trabaja con turnos de noche y llega a las ocho de la mañana y duerme todo el día, no le voy a decir “levántese a las dos a almorzar” Si al final no durmió nada en la noche, al menos que sea con alguien con insulina que ahí requiere ya otra, o sea que tiene que ser con más horario, pero…

**(I):** ¿Y en el trabajo?

**[COMENTARIOS GENERALIZADOS: “No en todos” O “En pocos”]**

**(PS):** De hecho, venir a los controles ya es un sacrificio para ellos.

**[TODOS ASIENTEN]**

**(PS):** Los piden distanciados en el trabajo.

**(PS):** Es bastante complejo, porque tienen horarios determinados para los almuerzos, uno tiene que decirles que por favor hable con su encargado para ir modificando los almuerzos o tienes que dar unas indicaciones, unas variables para poder suplir esa parte.

**(I):** ¿tienen esa facilidad?

**(PS):** No todos… [Silencio largo] No todos…

**(I):** ¿Qué es lo más complejo en términos de cambios en estilos de vida?

**(PS):** Yo creo, o sea, que la actividad física, yo creo eso, es no sé…

**[COMENTARIOS GENERALIZADOS: “La alimentación”]**

**(PS):** Yo creo que el chileno en general es flojo para la actividad física, pero… o sea, lo primordial yo creo, sobre todo en la diabetes es la parte nutricional.

**(PS):** Sí, pero a lo que voy yo es que por lo menos eso es igual hay pacientes que ese cambio de estilo de vida y hay pacientes que sí se compensan con la dieta, pero generalmente, la, la, los ejercicios es lo que no lo hacen y las justificaciones es el trabajo, pero por lo menos, yo sí he visto pacientes que modifican la dieta.

**[INTERRUPCIONES MÚLTIPLES: “Sí, la actividad física”]**

**(PS):** Desde mi experiencia, lo que me pasa a mí, no podemos generalizar, pero lo que uno ve en el tiempo, mayormente, es hipertensos como diabéticos, el tema es los régimen, por qué, porque pueden cambiar de trabajo, pueden caminar, la locomoción la deja a diez cuadras, pueden caminar las diez cuadras, pero caminan en otros lugares, y ya, logrando que caminen es bastante, pero el tema del régimen alimenticio es lo que más cuesta. Al menos, mi experiencia que uno ha aprendido, que tiene que comer esto, y esto, y la señora me dijo “yo sólo tengo lentejas esta semana”, ahí se te cae todo, todo el ámbito alimenticio, todo, uno tiene que ver la realidad en que estamos, una realidad socioeconómica y también una realidad cultural, donde el pan, la masa, la harina, es muy importante. Hay un buen número de personas que hacen su propio pan, entonces, hay cosas que nosotros por más que tratemos de influir de diferentes formas o maneras, nos cuesta.

**(I):** ¿Qué pasa en esos casos?

**(PS):** Hay caso y caso. Nosotros, yo creo, que acá tratamos caso a caso, cada uno de nosotros tiene su propia experiencia y por eso son caso a caso. Cada paciente es un caso, que a veces nos falta tiempo, porque hay cosas administrativas que hacer, llenar papeles, rellenar este… más que lograr una empatía o lograr algo... Y el número de horas, no tenemos las horas suficientes para los pacientes crónicos, porque la morbilidad es mucho más alta. Entonces, hay cosas que nosotros mismos hemos ido restringiendo, pero que es un trabajo que nos cuesta pensar que deberíamos primero empezar a tratar a los pacientes intolerantes, para que no llegue a ser diabético, que lo más importante, a este deberíamos de darle más horas, y ya, los diabéticos que realmente estamos compitiendo contra algo que puede llegar al final de su vida con insulina y al final de su vida con problemas de este… con complicaciones propias de la diabetes, porque no tenemos los medicamentos que nos puedan ayudar para eso. Hay muchos factores en contra, tenemos que hacerlo con lo que nosotros tenemos, con la educación que tratemos que dar, como les digo, es personal, llega a ser un tema personal con el paciente, porque hay pacientes que pueden venir y uno le dice “Con este otro amigo compartimos la misma experiencia con pacientes que es hipertenso y otro diabético”, yo le dije “usted es diabético, tiene que hacerse este examen y no sé qué… “Plump”, me tiró la puerta y se fue. Al doctor XXXX también le pasó lo mismo. Y él no quiere aceptar que es diabético. Entonces, ahí, ¿qué podemos hacer? Me entiende, o sea, también nosotros, desde la otra perspectiva, nosotros qué hacemos, si el paciente dice “nunca me dijeron”, no firmó el consentimiento de la notificación GES. Hay cosas que uno no puede hacer, intentamos hacer muchas cosas.

**[INTERRUPCIÓN: SOLICITAN SACAR UN VEHÍCULO, ¿ALGUIEN SE RETIRA?]**

**(I):** ¿qué pasa con los antecedentes culturales?

**(PS):** Es igual, se hace lo mismo, otro caso, otra paciente diabética, no se inyectaba la insulina, no se tomaba los medicamentos, fue a un Machi, el machi le quitó todos los medicamentos, hizo una insuficiencia renal, a los tres o cuatro meses falleció. El Machi le quitó todos los medicamentos, ahí tampoco podemos, culturalmente tampoco, ¿me entiende? Hay cosas que nosotros no podemos, hay barreras que no podemos traspasar ni entender, no haya una interculturalización con eso y de ellos con nosotros, pero cuesta, hay… como decía Philip en los pacientes nuevos es más fácil, ya que mucho de ellos están más informados.

**(PS):** Tiene más acceso a internet.

**(PS):** El acceso a internet, tienen miradas distintas, uno comienza a explicar y hay una aceptación a eso, pero si uno va con una señora de 47 años que siempre está en su casa y que siempre come las cosas que ella hace, hace su pan, y uno le dice “sabe qué, tiene que cambiar el chip” No lo hacen y se lo dicen a uno.

**(I):** ¿Son cambios generacionales?

**(PS):** Son cambios generacionales y todo eso, o sea, pero como le digo, o sea, cada paciente crónico para nosotros es único y es una experiencia diferente. Yo creo que el cambio cultural… ¿Qué hacer con eso? ¿Qué herramientas nosotros podríamos tener para poder hacer eso? Porque uno llega y hay diferentes cambios en las personas o en los grupos etarios que vamos viendo. Nos tenemos que movilizar en eso, nosotros somos, más que tratemos de estar al tanto de todo para que no haya tanta complicación.

**[SILENCIO PROLOGRADO]**

**(I):** ¿Algo más que aportar?

**[SILENCIO BREVE]**

**(I):** ¿qué pasa con la familia de los pacientes?

**(PS):** Depende de la familia, o sea, la gran mayoría son… este… de mujeres a cargo, son dueñas de casa, les encargan… o hay abuelas, los hijos trabajan, los hijos las mantienen, entonces, ellos se encargan de los hijos y… con los que le dan las hijas o los hijos se mantienen, pero a veces no hay apoyo, “doctor no pude llegar a la hora porque tuve que dejar a mi nieto al colegio o al jardín” y pierden las horas o… este... uno lo deriva a la enfermera o lo deriva al nutricionista, y “no, no pude venir porque esto, por esto, por esto” pero, o sea, al médico no intentan faltar, pero a veces los apoyos son bastantes necesarios para nosotros no están. Hay hijas sí que vienen, que se preocupan y están ahí, comienzan los cambios de hábitos y se ve que hay mejoría, ¿pero qué será como el 5% de toda la población?

**(PS):** Son pocos los que tienen el apoyo de la familia.

**(PS):** Quedan con los niños, también, que tienen obesidad o sobre peso, son pocas las familias que hacen los cambios toda la familia, “tú no puedes tomar bebida, pero nosotros sí tomamos”, entonces, el niño crece con eso “no puedo tomar porque estoy sobrepeso, estoy gordito”, pero pasa lo mismo con los pacientes diabéticos, compran torta “¡Ay! Es que yo no compro la bebida, la compra mi hijo y no le gusta light, porque dice que es mala, entonces, como él la compra yo tengo que tomar la de bebida normal no más”.

**(PS):** Hay también harto machismo, encuentro que, por ejemplo, cuando el hombre se enferma hay mucho más apoyo hacia a él, o sea, por ejemplo, se modifica la alimentación de toda la casa, en cambio si, por ejemplo, la mujer es la que tiene la enfermedad, o hacemos nada o el hombre, como que se niega, vamos a comer lo mismo o igual se compra la bebida, entonces… en el control también se ve y vienen los hombres acompañados de la mujer y la mujer se sabe los medicamentos y el hombre no se los sabe, y la mujer al final siempre viene sola o con la hija.

**(PS):** En ese caso, generalmente, el acompañante acusa al paciente.

**[TODOS RÍEN Y CONFIRMAN EL COMENTARIO ANTERIOR]**

**(PS):** Mejor que acuse, a veces.

**(PS):** Si fuese percepción no más, yo creo que, si viene solamente con la mujer, si hace mucho más fácil, o sea, mi percepción. Y cuando viene solo cuesta un poco más, lo mismo que decía aquí el doctor de repente se niegan que tienen diabetes o simplemente no se quieren tomar los medicamentos, aunque se le explique de otra forma o será más didáctico para explicar, no. Pero como la mujer los causa, también…

**[AFIRMACIÓN GENERALIZADA: “Sí, claro”]**

**(I):** ¿Algo más?

**(PS):** A mí también me pasa que como hay baja educación, como que son más reacios a entender algunas cosas. Cuando uno, yo soy siempre de cómo explicarle lo que es la hemoglobina glicosilada, de que ellos traten de ver sus exámenes y que se interesen en que vaya bajando eso es en lo que nos estamos fijando y generalmente me cuesta que lo entiendan, o sea, que se interese por eso, pero por ejemplo, cuando se nota, no sé po, cuando tenemos una situación un poquito más acomodada, le gusta que uno le haga eso, se interesan en eso, de hecho vuelven por lo mismo que al final uno le está mostrando le está enseñando más allá o el lado médico de eso, que todos deberían saber.

**(PS):** o leen y llegan preguntando sobre lo que leyeron o hablan en el mismo idioma, por ejemplo, me dicen “oh el HDL me mejoró” [RISAS] Como que aprendió lo que era el HDL, entonces, en cambio, en otros casos, como que uno le intenta explicar, pero como que ellos “no voy a aprender nunca” como que tienen una barrera de “no soy capaz de aprenderlo” entonces como que no le interesa lo que pasará ahí. Claro, como que se frustran, pero yo les digo, pero mire, escuche, y como que se enojan a veces, incluso “no si yo entiendo, yo entiendo [imita voz de enojado], si no soy tonto”.

**(PS):** Yo creo que un modelo sustancial, en general, que originalmente fue como instaurado esta cosa de “la consulta-respuesta”, tanto del médico como del profesional, nutricionista, enfermero, yo creo que no sé si es lo que el modelo óptimo para poder llevar un buen control cardiovascular de este tipo de patologías, sobre todo porque cuando supuestamente un paciente compensado se le ve cada tres meses, que lo ve un médico, un nutricionista, enfermero, y después de esos nueve meses lo ve el médico, pasa mucho tiempo, entonces, yo creo que el modelo asistencial que originalmente era diseñado para otra cosa al aplicarlo a este tipo de patología, este tipo de enfermedades, eeehh… no sé si es lo mejor, eeh.. planteando, quizás a lo mejor, un modelo en el cual cada una de las intervenciones pueda ser por un equipo, por ejemplo, multidisciplinarios, yo creo que sería distinto, podría abordarse de manera más integral una patología que es integral y que el manejo tiene que ser integral.

**(I):** ¿Ustedes ven siempre los mismos pacientes?

**(PS):** No siempre.

**(I):** ¿eso dificulta?

**(PS):** Sí, dificulta, dificulta mucho.

**(PS):** Que era lo que yo decía un poco igual al principio, o sea, nosotros somos tres, cuatro con la colega de extensión y entre los cuatro podemos ver al paciente, o sea, no hay un seguimiento de un solo profesional y lo mismo con los médicos.

**[INTERRUPCIONES VARIAS: “El paciente se aburre”]**

**(PS): [HABLA EXTREMEDAMENTE RÁPIDO Y CON FUERTES VARIACIONES DE TONO]:** Tienen empatía con uno de los médicos y después le toca con otro médico. Además, como dijo el XXXX, el tiempo. Yo creo que el tiempo, cuando uno va a la privada, puede ir todos los meses, te voy a recibir y todo, acá no, le toca en tres meses y, generalmente, quieren verse con el médico y no quieren, porque ahora te toca con un nutricionista y en general toca que lo vea el médico, y es ideal que lo vea el médico a lo mejor, y entonces lo deja tirado hasta que le toque de nuevo cuando ya han pasado como seis meses más. Además, es que, a veces tenemos que variar varios médicos a o x motivos y eso genera para el paciente, ya de por sí, un rechazo, de por sí ya es una obligación y lo hace mal, viene por obligación no porque quieran recuperarse, no porque su enfermedad, son pocos, quizás los nuevos, quizás esos sí, esos son más fáciles y todo tiene que ver también con el trato con los médicos: “y no me trató como el anterior”, “¿y cómo como el anterior?”, entonces, todo lo que pudiste haber hecho primero, y es así, y es así, eso pasa cuando vemos niños, cuando vemos adultos, cuando vemos niños, cuando vemos más adultos. Eso nos genera una complicación, entonces el modelo de salud familiar, cuando tú tienes un médico de cabecera que ve siempre a todos sus mismos pacientes, no ocurre siempre así aquí, como debería ser. Como dijo el doctor, la XXXXX de morbilidad es mucha, entonces es mucha morbilidad y nos tenemos que enfocar en procesos de promoción, de prevención, en el tratamiento de la glucosa, de la resistencia a la insulina. En eso nosotros estamos trabajando, entonces considero que es un mal trabajo, porque no estamos haciendo lo que deberíamos hacer. Entonces, estamos trabado de mejorar el camino, lo que hay, ya, hasta que se complique, uno trata de explicarle eeh… eso. Y como cada paciente uno lo recibe de una forma. A uno hay que alzarle la voz, al otro hay que explicarle con calma para que entienda, al otro hay que explicarle más lento, al otro viene la hija, al otro… XXXXXXX… eso es un modelo, a veces, que no lo hacemos, que tratamos por cumplir algo que es de la implementación GES, sino nos llevamos una tremenda amonestación todos y perdemos la esencia por la que estamos atendiendo al paciente y eso, aunque no quiera el paciente, también se da cuenta.

**(I):** Muchas gracias, creo que cubrimos todo lo que ustedes consideran importante. Gracias por el tiempo y por compartir esas experiencias.

**HEALTH CENTER 2- HEALTH PROVIDERS**

**Investigador (I):** Ok, empezamos a grabar, partir por agradecer su presencia y que quieran compartir con nosotros su experiencia como profesionales de atención primaria, vamos a comenzar colocando el tema en la mesa, lo que nosotros queremos explorar es su percepción de lo que significa para los pacientes diabéticos vivir con la enfermedad. El que quiera partir.

**Proveedores de Salud (PS):** Parto Yo

**(I):** Perfecto

**(PS):** Encuentro que es un impacto súper grande que genera, primero, un rechazo y una crisis emocional; o sea ellos sienten un tema que es casi que es un designio de muerte, muchas veces se ponen a llorar, muchas veces genera un cambio que pasa desde la etapa de negación, por ejemplo, a una etapa de… tal vez, nunca se mueve de ahí en algunos pacientes, va evolucionando dependiendo del apoyo que también se tenga, porque hay que descartar las patologías en salud mental que también tiene

**(PS):** Entonces, también hay un tema de duelo, que a veces se queda un poco estancado. Entonces, a partir de ahí es el trabajo que de repente hay que ir manejando, pero es un tema súper complejo, sobre todo al inicio.

**(PS):** Eemm yo veo una diferencia en, yo soy la doctora **[se ríe]…**

**(PS):** Yo veo una diferencia eeh… al decirle a un paciente que es diabético entre las personas que han tenido experiencia con la diabetes antes y otras que no han tenido. Por ejemplo, si la persona, eeh… su mamá ha sido diabética, eeh su papá o la abuelitaaa, ha cuidado diabéticos, eeh sabe que es lo que va a pasar si no se cuida la diabetes, sino se toma los medicamentos; eeh esas personas, como que ya eeh vienen pensando, en que van a ser diabéticas en alguna oportunidad, y cuando tú les dices, “bueno ya está confirmada la diabetes”, ya mentalmente están como más preparadas, lo asumen de mejor manera y ya han empezado a cuidarse de antes; porque no quieren pasar, por la experiencia que paso su mamá o su abuelita, y que le cortaron el pie, que quedo ciega, que no sé qué. En cambio, los pacientes que no tienen **[se ríe]**, como antecedentes o contacto con la diabetes, esos si lo toman súper

**(PS):** Lo que pasa, es que yo pienso, que hay un tabú, con lo de la enfermedad,

**(PS):** Primero que no deja de ser una enfermedad compleja.

**(PS):** Y que muchos de estos pacientes nunca realizan de pronto un manejo adecuado de su enfermedad como tal, porque tú como profesional haces tu manejo con lo que puedes, pero hay mucho que se juega en parte de ellos y que muchas veces no cumplen o piensa que no es tan severo, porque muchos pacientes…… Como hay pacientes que tienen conocimientos que la enfermedad es algo que deben cuidar, que hay que manejarla, que hay que controlarla; que hay que llevar, cambiar hábitos en la vida. Hay muchos que no le dan tanta importancia porque no tienen mucho conocimiento, de los que se trata la enfermedad como tal, entonces tú le puedes inducir la modificación de factores o de hábitos, y estos pacientes no hacen esos cambios, porque creen, que no les va a pasar nada.

**(PS):** En general, también, tiene que ver con el tema del autocuidado, y eso no pasa solamente en la diabetes, sino en general en todas las patologías; en que la conciencia de auto cuidado no existe, empoderadamente. Principalmente, se encuentra más el tema del paternalismo, es decir, la responsabilidad de mi glicemia, de mi peso o de mis exámenes que estén normales, es del médico, de la nutricionista, o de cualquier otra persona, menos de ellos mismos. Entonces, “como, es que yo no bajo de peso, porque la nutricionista es mala”, por ejemplo.

**(PS):** Pero no tiene que ver con que no baja de peso o no modifica la glicemia, o no acomoda sus exámenes por un tema de que no está empoderado en su auto cuidado y las decisiones no la tomas, está en una etapa principalmente de negación o pre contemplativa o como quieran llamarse, entonces, el proceso del auto cuidado no se logra.

**(PS):** Yo también creo respecto a lo que preguntabas de donde viene el miedo, eeh… la diabetes para los pacientes, lo que yo visualizo, es que es una enfermedad presente y ausente, esta como que tiene esa dualidad. Está presente porque lo ve o porque lo sabe o porque lo escuchan en las noticias, lo ven en la televisión, pero creen que nunca les va a pasar, hay pacientes que creen que nunca les va a tocar a ellos, y desde el momento en que les toca, sienten ese terror por todo, porque “les llega la información de una”, todo lo que han escuchado en la tele, lo que han visto, etc. Eeh… y después, ocurre lo que decía Benilde, ahí viene el miedo y después la negación, eeh… y como decía Benilde, también, eso se queda estancado muchas veces porque el paciente llega, dice que se siente bien, que no tiene nada, porque no presenta síntomas, y uno al explicarle notan en, la comunicación no verbal, el rechazo, entonces, creo que el miedo también, principalmente, ocurre por los pacientes que no han tenido familiares cercano, porque de una les viene ese como corto circuito, de todo lo que han venido escuchando en otros medios.

**(PS):** Igual que también han visto cómo avanza la diabetes, “ooh que a mi vecino le amputaron…”, o que “mi otro amigo está dializándose”, o porque el otro quedó ciego producto de la diabetes, entonces ellos ven el proceso de la diabetes, como el caos y no, la consecuencia de una reacción que tiene que ver con las medidas que ellos mismos toman.

**(PS):** También veo eso, que los pacientes ven como una enfermedad con mucho miedo porque creen que tienen que ¡cambiar todo!, con respecto a su vida

**(I):** Ya

**(PS):** Piensan que ya nunca más van a poder comer un dulce, nunca más van a poder tomar bebida, ven algo como que su vida tiene que cambiar completamente y en el fondo, a la vez, están pensando en que no quieren hacer eso, no quieren cambiar drásticamente. En cambio, cuando yo converso con el paciente, y le puedo dar un poco más de tiempo, me da esa posibilidad, de que no se da en cada control, porque generalmente en el control es muy poco el tiempo pero uno ve que no llegó el paciente y le da un tiempo más para conversar y para explicarle: que no es un cambio radical, que lo que tiene que el saber en cosas que pueden cambiar, que igual pueden comer un día una torta, lo que no tiene que hacerlo es todos los días, entonces, ahí el paciente empieza como a entender un poco más, a perderle un poco el miedo a la enfermedad, y lo otro que ven siempre al diabético que está mal, a ese diabético que le cortaron el pie, que está ciego, ¿ya? Pero cuando uno les dice “pero ¡mire el paciente que salió antes que usted, es diabético de tantos años y está compensada su diabetes y usted lo ve que es una persona normal” entonces, ellos pueden…empiezan como a aceptar un poco más que la enfermedad, no es una cosa tan grave, tan catastrófica, como al principio lo decían, yo creo, que ese es el miedo que tienen y reflejan.

**(PS):** Bueno, yo creo que el gran problema a acá, es que nosotros vemos la patología obviamente del punto de vista profesional, y muchas veces nos podemos equivocar con el usuario en este caso, y el gran problema, es el aspectos de los hábitos, y los hábitos no se cambian porque yo tengo diabetes, los hábitos no se cambian porque mi mamá tuvo diabetes y me puede pasar algo, los hábitos no se cambian de un día para otro, y nosotros lo que hacemos es poner reglas y explicar si bien tu labor como profesional es “explicar lo que tu tienes, lo que tienes que hacer y que no, es decisión tuya como persona tomar o dejar”. Somos personas adultas en lo cual podemos decidir, pero a la vez también hay un problema eeh… que yo encuentro que es un tema social

**(PS):** ¿Por qué? Porque lo que decía el colega de que, por ejemplo, la señora quiere comer torta, no hay diabético que no quiera comer torta, no conozco ninguno, pero quiero que lo vean hoy día… el otro día yo hablaba con una paciente y me decía “¡es que la nutricionista me hace comer verduras y que me hace comer pavo!”, hijito me dice, “yo tengo una pensión de cincuenta mil pesos, ¿De dónde podemos hacer eso?” entonces, yo estoy haciendo visitas integrales, en la cual la idea es bajarla a la realidad….

**(I):**¿Qué es visita integral?

**(PS):** De paciente descompensado. Entonces, la realidad es otra. Porque yo no lo puedo decir a un paciente que tiene una pensión de cincuenta mil pesos, que coma verduras todos los días, si cuando hoy día una lechuga vale 1.200 pesos, 1.500 pesos, que ellos con 1.500 pesos comen pan tres días, cuatro días, no sé. ¿Me entiende?, entonces, pasa por un tema económico, pasa por un tema social, en la cual, ya póngase usted, no sé, la…la...pauta para diabéticos cuestan 3 o 4 veces más**.** Para un adulto normal, no hay producto para diabéticos, recién están saliendo… o sea la población aumenta y todas las cosas, en general, recién están saliendo, entonces, limitamos mucho a la gente. Si bien, es lo que debemos hacer porque no tenemos de otra, pero también es un tema de población yo creo que culturalmente estamos, también, retrasados….

**(I)** ¿ustedes querían opinar?

**(PS):** las dos somos nutricionistas

**[Hablan al mismo tiempo] [Risas]**

**(PS):** ¡Acá discrepamos!, ¡y yo soy súper franca!!, discrepo absolutamente la opinión dada en ciertos puntos. Ya, ¿Por qué?: Porque el tema no tiene que ver con que la nutricionista le dijo que coma verduras, es que ella necesita ¡que distribuya los gastos económicos! de una forma más inteligente: ¿De qué forma?, Priorizando la alimentación, ahora eso se logra en el minuto que la paciente, el usuario logra ver la importancia de que sus decisiones sean las apropiadas, si yo le digo, “¡cómete el trozo de torta!” no estoy cumpliendo mi labor…

**(PS):** Totalmente de acuerdo…

**(PS):** Lo que hay… eeh… Sí… el hábito se logra cuando logra mirar su decisión como impacta en su salud, no existe ninguna otra alternativa, no se trata de que alguien le diga que haga o no …

**(I)** ¿Cómo se logra?

**(PS):** ¿Eso? Concientizando y empoderando al individuo…

**(PS):** Con educación

**(PS):** Eso, es el tema, es tirándole la pelota a ellos, el punto es que lamentablemente, muchas veces, el equipo de salud no habla el mismo lenguaje**,** “Juanito le dice una cosa, Pedrito le dice otra, el médico le dice otra”, por lo tanto, es un tema súper complejo, que haya un mensaje tan irregular, dependiendo con el profesional con que le toque y dependiendo con el criterio que se toque….

**(PS):** Entonces, lamentablemente si no hay una unificación de criterios y no hay una unificación de las consecuencias de los que uno comenta, empodera al paciente en una decisión….

**(I):** Ya

**(PS):** Obviamente, todos no vamos a hablar el mismo lenguaje, y eso, esa contradicción, va a traer al paciente a un caos aún peor; ¿Por qué? Porque…

**(PS):** Yo concuerdo totalmente, de hecho, iba a nombrar lo mismo que Benilde, y también, hacer hincapié… eeh… de esto de bajar a la realidad, muchas veces el paciente te dice “me tomo un litro de bebida, fumo cigarro” entonces, respecto a la distribución inteligente es mejor esa plata, gástela en frutas y verduras

**(PS):** Muchas veces yo he calculado cuanta plata gastan en bebidas, y estamos hablando de más de 500 mil pesos al mes en bebidas!!!, o a veces en cigarros gastan treinta lucas, entonces no tiene que ver con que, nosotros como nutricionistas, en este caso voy a defender como la posición

**(PS):** Es que no es un tema en particular

**(PS):** No, no, no, es el punto es el punto, de cómo nosotros logramos hacer que eso de la alimentación saludable baje a la realidad

**(PS):** Es que no es solamente a lo mejor alimentación saludable**,** yo creo que el tema pasa acá por el tema que tu habías nombrado al principio, estamos en una sociedad que actualmente nos guste o no somos paternalistas, o somos maternalitas

**(PS):** Claro

**(PS):** estamos acostumbrado a echarle la culpa a los demás no nos hacemos responsables hoy en día de nada. Y hoy día lo vemos reflejado en lo que está sucediendo en la actualidad, nadie se hace responsable todos queremos que nos regalen, pero nadie tiene obligaciones, y eso es así, y esto va a pasar, con los adultos, tenemos que darnos cuenta de que a las finales si…

**(PS)**: O los niños

**(PS):** Cuando vas hacer entender en un adulto mayor estos cambios de hábitos, “perdóname llevo 65 años haciendo esto, como vas a venir tu a decirme ahora que tengo que hacer esto otro”, “oye yo cuando era chico…” el otro día me decía un paciente “yo cuando era chico no tenía que comer, nunca me pude tomar una bebida, y yo hoy día que puedo tu vienes y me dices que yo no puedo tomar bebida”, “caballero pero es que cuando usted estaba chico no estaba enfermo”, “Bueno, las cosas pasan por algo, pero yo hoy día que puedo lo voy hacer”, “caballero es su decisión mi deber es informarle que es lo que le va a pasar”

**(PS):** Es entregarle herramientas

**(PS):** Dar herramientas para que él tome su decisión

**(PS):** por supuesto, pero es que ahí está el tema

**(PS):** pero es que ahí está el tema, porque si tenemos una sociedad paternalista pasara netamente por el punto de vista de nosotros profesionales, el darle información o es un problema social

**(PS):** O sea es un problema social pero el rol que nosotros tenemos como funcionarios de la salud se dejó de plantear como una situación paternalista hace mucho rato, desde que empezamos a ser CESFAM, el punto es que el proceso cognitivo de los pacientes no paso, y muchas veces pasa esto, de que te tiran la pelota a ti, porque tu no logra compensar o no… no sé, el típico hipertenso “es que yo siempre he tenido la presión alta”, y como kilos de sal, o sea ¿de quién es la responsabilidad del médico o suya? El tema de las decisiones, por qué, porque siempre están en etapa que no va a asumir su decisión

**(PS):** pero si mira, si… si… resumimos todo esto o sea aquí…

**[Risas]**

**(I):** hay algunos aspectos concretos que quiero que los profundicemos.

**(PS)**: Ya, por lo menos yo voy a hablar de mi caso, yo por lo general en los controles siempre les pregunto al paciente ¿Qué entiende usted por diabetes? Porque de repente los pacientes ni siquiera… o sea saben que tiene que tomar el médicamente, saben que no tienen que comer esto, pero no entienden así como a grandes rasgos que es lo que sucede en su cuerpo, entonces, yo les digo “dígame lo que usted piensa, y lo que usted crea, yo estoy aquí para que hablemos” y ahí me empiezan a explicar y de repente tienen ideas súper erróneas de lo que es la patología, entonces, que es lo que pasa, yo les enfoco porque cuando no se po, estamos con riego de ulceración del pie diabético, le digo, “estas son consecuencias que tiene la diabetes por esto y esto, y tengo unos folletos que les muestro con cosas gráficas, entonces, que es lo que hacen en el paciente que entienden porque deben cuidarse, entonces de repente son preguntas súper sencillas pero que sirven para que ellos se hagan gestores de su auto cuidado, y a mí me ha funcionado…

**(PS):** bueno, yo soy médico, lo que en realidad en el tiempo que tengo destinado y que trato de ver es más la parte médica, la adherencia a fármacos, el daño al órgano blanco, revisar los exámenes, ver si esta con trastorno de hidroelectrolítico, dentro del tiempo que me queda, como para hacerle educación al paciente, ¡un poco!

**(PS):** Cero comas cinco segundos…

**(PS):** La impresión que a mí me da, es que de la parte nutricional los pacientes en realidad tienen como un trastorno anímico importante, como por las dificultades del día a día, o sea…

**(I):**¿y el día a día?

**(PS):** Mira más que nada siento que son pacientes que tienen que trabajar muy lejos, que se tienen que levantar muy temprano, que tienen la dificultad para transportarse que eso ya como que es un paciente que no es feliz, que tiene muchos problemas, que además viene de un estrato económico que no es el mejor, o sea que la plata es un tema para estos pacientes, y además, sobrecargarlo con que… o sea lo poco que le produce felicidad que es comer, o sea comerse algo rico, hacer una pausa, conversar con los colegas, etcétera, quitarles eso es como ¡quitarle el mundo!, es como pucha, uno trata de decirle que tienen que tratar de entender que es por su bien, que van a vivir más, pero yo creo que ese es el punto, quizás no quieren vivir más, como que no les importa vivir más, porque ya están tan estresados en una sociedad que los tiene muy estresados, que quizás tendríamos que partir primero cambiando lo otro y la parte médica, pucha, lograr cambiar lo que alcancemos, lo que se pueda pero esa motivación que viene del paciente no está, yo no la veo

**(I):**¿Cómo ven ustedes la factibilidad de que ellos cumplan en su vida diaria, con las indicaciones?

**(PS):** Lo que pasa es que todo depende de cuánto el paciente conozca su enfermedad, es decir, si yo conozco algo primero no va a ser un mito y segundo voy a entender que las decisiones que voy a tomar van a tener un impacto positivo o negativo, no es quitar, no es cambiar el hábito de forma definitiva porque es imposible, es hacerlo entender que en realidad el proceso ha estado equivocado todo el tiempo, es tratar de que, de acuerdo, a las condiciones económicas que tiene poder hacer optimizar el recurso, como lo había mencionado, pero poder entregar a él las herramientas para hacerlo mejor y más feliz, por ejemplo, perfecto, “no tengo plata para comprarme una torta para diabéticos pero tengo harina y huevos lo puedo hacer yo en la casa con endulzante”, pero eso es la decisión del paciente.

**(I)** ¿cómo imaginas eso?

**(PS):** lo que pasa que para eso para ellos es una responsabilidad, por lo tanto, salen del box y no tan solo nutrición sino también médicos cierran la puerta y se olvidan de todo

**(PS):** Es que eso depende mucho del paciente, hay pacientes y pacientes, o sea no todos son igual, hay pacientes que uno ve cuando están en la etapa ya contemplativa, se notan preocupados y uno lo percibe en ciertos detalles, por ejemplo, “ellos solos te empiezan a decir puchas me he estado esforzando, ¿Qué tengo que hacer?”, uno nota ciertos mensajes que el paciente te va dando y uno dice “ya, con este sí”

**(PS):** o uno trabaja un punto específico, y cuando va saliendo hay algunos que dicen “ya voy a poner todo de mi parte” y se van, en cambio hay otros que llegan se sientan con la comunicación no verbal así… echados para atrás, con una actitud totalmente desinteresada, entonces, la adherencia depende de cada paciente, del momento por el que esté pasando, del estado anímico por el que esté pasando, eeh… de la mochila que traiga de su casa…

**(PS):** Depende, claro… depende uno también siempre les pregunta con quien vive, quien cocina, ¿tiene apoyo de la familia? Eem… depende mucho, porque hay personas que te dicen “no en mi casa yo lucho contra la corriente porque yo llego y esto solo y el resto, mi marido no le gusta la bebida light, llega con la Coca Cola normal y yo me veo tentada…” y hay otros que uno los visualiza bien, y uno dice “sí, es posible, llego a su casa ya cerró la puerta y se va a poner las pilas”

**(PS):** Es que depende muchas veces esos pacientes que son un en un millón, en un millón, es el que genera un cambio a nivel de la familia, y muchas veces, también tiene que ver con que nosotros como somos un CESFAM intervenimos a la abuela, la abuela es diabética, e hija obesa, nietos obesos, o sea, depende de cómo logramos hacer que mire diferente y que ella es la que puede permitir que su nieto, no tenga una patología crónica, le cambia el switch y le permite como tener una visión un poco más salvadora, así como de una luz de esperanza que signifique para ella que su decisión va impactar a algo que realmente quiere, porque muchas veces ellos se olvidan de sí mismo, les importa poco, como no sienten nada no tienen como… no saben la gravedad de las decisiones que están tomando.

**(I):** Ok, ¿tú querías aportar algo?

**(PS):** Sí… eeh… lo que pasa es que yo no veo pacientes crónicos acá en el consultorio, sino, que los veo en las casas, porque estoy en el programa de postrados, entonces, en el programa postrado están todos los pacientes aquellos que ya han sufrido las consecuencias secundarias de la diabetes **[se ríe]** mal manejadas durante años, tenemos personas con secuela de accidentes vasculares, personas con ceguera, etcétera. Pero hay una cosas que nosotros notamos pero siempre en el programa postrados una vez que estos pacientes diabéticos que han pasado año con descompensados y todo eso eeh… se postran y empiezan a mejorar, y la hemoglobinas glicosiladas fantásticas, de seis, de cinco, ocho, y es que han dejado todo su eeh… en mano de su familia la alimentación, ya no son ellos los que se preparan la comida, no son ellos los que comen, sino que es otra persona la que maneja su alimentación, estamos hablando del familiar que está cuidándola, la hija, etcétera

**(I)** ¿Cuidador?

**(PS):** Claro, cuidador, entonces, además en el programa postrado nosotros contamos con una nutricionista que va a la casa y se está toooda … un hora o más a veces, en la cocina con la persona que cocina, en este caso que no es el paciente con la personas que… y están viendo que materiales cuentan, como preparar cosas, que podemos usar, que no podemos usar, ya entonces, se compensa la alimentación, se compensa la diabetes, se compensa toda la enfermedad, en ese trabajo que se hace en casa con la persona que prepara los alimentos, pero ya el daño está hecho, o sea, la persona como dicen los chiquillos no se ha cuidado, ella misma no se cuidado, y estamos viendo las consecuencias de todo esto, los pies, las amputaciones, y todo eso y se llegan a… terminan postrado y terminan compensados, entonces…

**(PS):** en vez de la compensación darse antes para que no ocurran estas cosas las estamos viendo después porque la familia, ya cambia, cambian los hábitos…

**(PS):** estoy completamente de acuerdo con la doctora, yo creo que una de las cosas que más nos faltan para que los usuarios de nosotros puedan compensar su diabetes, es la educación, falta muchos tiempo de educación, tenemos muy poco tiempo en lo que es un control cardiovascular ya sea nutricionista, de enfermera, de médico, para dar educación, porque tenemos obligatoriamente que remeternos a llenar formularios, anotar exámenes, todo y el tiempo de educación es muy poco. Yo estaba en otro CESFAM y tengo una experiencia, nosotros empezamos a trabajar con los pacientes descompensados, sacábamos a todos los pacientes que tenían hemoglobina sobre diez y los íbamos a ver a la casa, y hacíamos ese trabajo que está diciendo la doctora que hacen cuando ya están jodidos, están postrados. Eran pacientes que los íbamos a ver antes de que llegaran a eso, y teníamos unos resultados que eran ¡increíbles!, pacientes que estaban con hemoglobina de doce, de diez, de ocho después de una visita de ir a la casa con la nutricionista, el enfermero, a explicar, conversar con toda la familia, darle tiempo de explicarle lo que era la enfermedad, que entendiera que si hacían tal cambio y no negándole que no podía comer nada ninguna cosa, si uno de ustedes de repente se puede dar un gusto, pero usted lo va a ver cuando usted tenga compensada su diabetes, si usted está bien y un día hay un cumpleaños, aniversario, y la torta… no es de la torta sin azúcar, se puede comer un pedacito porque eso no la va a descompensar, y logramos que entendiera que se tomaran la medicación, a veces era que no se tomaban la medicación en horario, entender que toda la familia participara en el cuidado, porque era un cuidado para el diabético y para todos lo demás, porque si no después ya no va a volver, como pasa, como paso con todos nuestros pacientes que tenemos toda la familia, la abuela diabética, la mamá diabética, y el hijo esta obeso y va a ser diabético en un par de años más, entonces, cuando íbamos a trabajar con la familia y teníamos el tiempo para explicar cómo es la enfermedad, por qué es diabético, qué significa que nosotros… a mí me ha pasado muchas veces en box que les pregunto “señora ¿y que entiende usted por diabetes?”, “no la diabetes es que no tengo que comer nada dulce, porque la nutricionista me dijo que no tenía que comer nada de esto..” es como negación de todas las cosas, “¿pero, por qué usted es diabética? Si yo no soy diabético y yo puedo comer diles, ¿Cuál es la diferencia?”, no saben que es la diabetes, partir, entonces si no saben que es la diabetes, nunca van a entender que tengo que restringir comer carbohidratos, tengo que hacer ejercicios, tengo que hacer cosas porque “no sé, ni siquiera *qué significa que ser diabético”*, y a veces, no es un problema de dinero, ¿ya? Porque nosotros también tenemos usuarios que tienen buena situación económica, yo tengo amigos, tengo familiares que tienen muy buena situación económica y pasa lo mismo, ¿ya? O sea, no es un problema de que no pueden comprar las verduras, no, simplemente que les falta el conocimiento, les falta internalizar más lo que es la diabetes para poder… una vez que yo sé hacer que les vaya así, entonces, no tengo que tomar bebida todos los días, sí de repente si me gusta tanto tengo que probarla porque,… pero esas… eso en el tiempo es una de las cosas fundamentales que tenemos en contra en el control, en el tratamiento de la diabetes, y por eso, la cantidad de diabéticos descompensados que tenemos me quedo aquí.

**(I)** ¿Usted quería aportar?

**(PS):** Si, es que lo que yo pienso en cuanto a lo de la patología es que, primero, ante cualquier cosa debe haber una aceptación del paciente a la patología, para tu poder hacer una educación tiene que haber aceptación del paciente, porque si no hay una aceptación tú puedes agotar todos los recursos que quieras en educación y no lo vas a lograr. Segundo, hay mucho de estos pacientes que por mucho que tú los eduques son reacios a seguir de pronto de cierto modo no rígidamente un tratamiento o una pauta, de hecho, hay pacientes que son muy malos para los hipoglucemiantes o, que por lo menos, los hipoglucemiantes orales no les caen bien…

**(I):** me quiero detener en eso ¿Qué pasa con los medicamentos?

**(PS):** hay pacientes que tienen muchas intolerancias a los medicamentos para la diabetes, eeh… más dirigidamente hacia la Metformina que es con la que más tienen molestias gástricas, molestias de muchos tipos, pero hay pacientes que, si realizan tratamientos medianamente normales, pero no hacen pauta, ¿ya? Pauta dietaria, pautas de esos tipos, ¿Qué sucede? Que hay pacientes de estos que por más que los quieras controlar no los vas a poder controlar y ya los tienen con una carga máxima de hipoglucemiantes orales y no tienes respuestas, entonces, ahí es donde tu entras a jugártela con la proposición al paciente tú vas pasando tratamiento de insulina y es, pero aun cuando tú haces esta propuesta, porque si no aceptan la patología de punto base con medicamentos orales…

**(I):**¿orales?

**(PS):** en teoría, sí, muchos…

**(PS):** En farmacias no

[**Hablan** **varios** **al** **mismo** **tiempo]**

**(PS):** En la casa cuando uno va a domicilio se da cuenta que tienen montones de Metformina

**(PS):** Y las visitas dicen que tienen una cantidad de fármacos que les sobran

**[Siguen hablando al mismo tiempo]**

**(PS):** porque algunos dicen que les hace mal para el estómago, así de simple, y otros que tienen unas cargas muy grandes de medicamento, hay pacientes, si tú tienes un paciente diabético es raro que no tengas, además de hipertensión, o que tenga

**(PS):** Dislipidemia, enfermedad renal…

**(PS):**: un problema de hipertiroidismo, dislipidemia, o que estén con un anticoagulante o que, además, más encima sean EPOC, entonces, están con inhaladores, entonces, tu sales con unas listas así de medicamentos, entonces, de repente igual los medicamentos que nosotros tenemos aquí para la diabetes aquí no son muchos, tenemos Metformina y Glibenclamida , son dos, generalmente, la insulina bueno, si son insulinos, pero son pocos los medicamentos para la diabetes, no es el problema… el problemas son los demás, porque hay pacientes que usan dos antihipertensivos, eeeh, y uno o dos diuréticos, eehh…

**(PS):** es que…

**(PS):** Es que hay millones de alternativas

**(PS):** yo creo que, por la polifarmacia, o sea, creen que tomar tantos medicamentos o sea… hay un nivel de mala información que por tantos medicamentos pueden tener fallas renales finalmente, pero no, no saben cómo que medicamento ni para qué medicamento es qué cosa

**(PS):** Se los toman todos juntos

**(PS):** Es que es una locura porque…

**(PS):** Y si trabajan no se toman ninguno

**(PS):** es que lo que pasa, es que cuando tú tienes una polifarmacia, no tienes que eeh.. cargar al paciente o seas estar pendiente todo el día de los medicamentos, que el horario, que esto que lo otro, sino que tiene que tratar de optimizar los horarios, ya algunos medicamentos en la mañana, otros al mediodía, otros en la noche, pero si pones “separe este medicamento del otro por media hora, una hora”, no, ahí es demasiada carga

**(I)** ¿Y por qué es tanto trabajo?

**(PS):** sí, pasar pendiente de un medicamento o sea yo misma, yo misma como paciente soy remala porque…. No, de verdad si me dicen un medicamento cada ocho horas no chao, chao…

**(PS):** Tiene que empoderarse el tema del auto cuidado, es mi responsabilidad tomarme los medicamentos

**(PS)**: lo que pasa que ella lo ve como…

**[Hablan al mismo tiempo]**

**(PS):** desde el punto laboral, es bastante complejo porque si nosotros necesitamos a veces tomar algún medicamento por un dolor y lo vas a tomar cada ocho horas, tú te tomas el primero, pero cuando quieres ver ya te ocupaste tanto trabajo tanto el tiempo que cuando volteaste a ver ya llevas diez horas abajo, o sea… entonces ya perdiste la secuencia o sea si somos nosotros iguales en ese… ellos igual

**(I)** ¿habrá facilidad para sus remedios? Por ejemplo, en el trabajo

**(PS):** yo por lo menos trato de preguntarles, les hago casi siempre que puedo un calendario, el típico calendario que uno le pone ya las lunas el sol bien grande, el número de pastillas, no en número en pastillas, para que entiendan

**(PS):** sí, porque hay muchos pacientes que son analfabetos

**(PS):** si uno les pone un medio con fracciones, no saben quizás se va a confundir, igual cuando llegan yo les reviso el carnet tienen mi calendario así hecho subrayado con destacado y “¿se lo toma así?”, “no, ¿por qué?, no sé”, así como que, no sé, no captan esa información que uno les da, ¡y la tienen!, entonces, ahí esa falla no sé por qué pasa

**(PS):** o a veces relacionan, por ejemplo, una sintomatología inespecífica con el medicamento y lo suspenden, y ¿por qué no pregunto? “Es que yo caché que me hacía mal entonces lo suspendí…”

**(PS):** lo suspendí y pasó la molestia, capaz fue el medicamento….

**(PS):** claro, “¿y sabe que consecuencia tuvo?” “No” “¿y por qué tiene la presión alta ahora?” “haa es que como no siento nada” **[Silencio]…**

**(I)** ¿Qué pasa con la actividad física?

**[Exclaman los participantes “Oooohhh” y se ríen de la pregunta]**

**(PS)**: Ellos dicen: “¡Yo trabajo, no me queda energía porque trabajo mucho!” “y me muevo…”

**(PS):** “camino media hora para ir a tomar la micro…”

**(PS):** “cuido de los niños…”

**(PS):** Eso “cuido de los niños, los voy a dejar…” voy para acá y para allá…” “camino de la pieza...”

**(PS):** “No hay tiempo…”

**(PS):** Asocian el trabajo de la vida diaria con actividad física

**(PS):** “No hay tiempo”

**(PS):** y cuando uno les habla de otra clase de actividad física “eeh… no tengo tiempo, es que yo no tengo tiempo y no lo voy a hacer”

**(PS):** O hay gente que te dice, “no, no me interesa, no quiero hacerlo”, “no es que tengo la artrosis en la rodilla”, “tengo una hernia en no sé dónde”, “tengo…”

**(PS):** “Me canso, también me canso…“

**(PS):** “me canso”, la mayoría de los que se cansan y tienen todas esas patologías obviamente hacen menos actividad física

**(I)** ¿Hay muchos pacientes con insulina?

**[Todo exclaman de acuerdo “Siiií”]**

**(PS):** Yo una vez trate de hacer como ese estudio y vi que en realidad que el riesgo relativo de los pacientes con insulina terapia era el doble de presentar depresión, entonces nuestros pacientes en realidad caracterizándolos; los que están con insulina, también están deprimidos, o sea y ahí no alcancé hacer como el estudio de cómo saber que era, primero, si la depresión estaba asociada a la insulina o ya en realidad como factor descompénsante la depresión, que descompensara su diabetes, pero efectivamente son pacientes que les cuesta. O sea, que ya tener el estrés de inyectarse, genera a un trastorno anímico, asociado.

**(I)**¿Qué pasa con las familias?

**(PS):** Yo por lo menos veo que se preocupan, nosotros acá, cada sector hace taller de insulina, entonces, cuando hay un ingreso de insulino requirente, el doctor da la orden y nosotros hacemos un taller de educación, y por lo general, cuando son adultos mayores se les pide que vengan acompañados de otros familiares

**(PS):** Es que en esa charla ahí obviamente…hay gente que rechaza también el…

**(PS):** Tiene poca adherencia también, antes nosotros teníamos también varios talleres educativos, enmarcados en distintas patologías, pero el cumplimiento, o sea es decir la asistencia era nula, nada… o sea ninguna, o uno o dos porque era mucho tiempo, porque preferían que los viera el médico, o porque no querían y dejaron de venir, entonces, lamentablemente dejamos de hacer esos talleres

**(PS):** Uno citaba a diez, por ejemplo, diez, doce o veinte, y llegaban tres o ninguno, uno, dos a veces ninguno, la… ahí uno nota que, también, no les importa mucho más le importa el control con el médico, voy al médico y si es eso voy

**(PS):** pero es un tema social

**(I)** ¿Cómo así?

**(PS):** Haber… Acá, El paciente, es ¡Medico!... Dios

**(PS):** Lo que diga el médico es lo que ellos hacen

**(PS):** Porque es una doble versión, también, es un doble discurso, es más fácil responsabilizar a este médico Dios de lo que le pasa, porque no es culpa de él

**(I)** ¿Cómo lo ve el resto?

**(PS):** a mí me pareció eem… esta actividad que hacían ustedes en terreno como súper buena porque es quitarnos un poco el peso… ya, el paciente tiene que venir al consultorio con toda la carga emotiva que tiene tener una enfermedad crónica, que va a ser para toda la vida, que le va a dar problemas, eeh… es llevar eemm… al consultorio a la casa al paciente el trabajo que nosotros hacemos, por ejemplo, con familias, trabajo en familia que vamos a terreno, ese se debería hacer con todos los pacientes descompensados, porque a lo mejor obtendríamos… sería la parte educativa como mas eeh… llegaría más a ellos eeh… podríamos hacer con equipos multidisciplinarios donde se tocaran lo temas de la alimentación que el enfermero, la parte de los pies, el… incluso el médico podría bajar en terreno y yo creo que sería más efectivo que traer al paciente acá y disgregarlo a que hoy día tiene que ver al médico, en dos meses más a la enfermera, dos meses más a la nutri, no….

**(PS):** El no tener que llenar un montón de documentos de fichas, de formularios y poder conversar con el paciente y tener una interacción con él; saber por qué él no se está tomando la medicación, “dígame por qué no se la toma”, y que él te diga la verdad “no me la tomo porque pasa esto, pasa esto otro”, “¿Por qué cocina tal cosa que no le corresponde? la nutricionista, ¿cuál es su plato?, ¿en qué plato come?, el clásico “yo me como un platito de comida” y… es una fuente, [**comenta** **otro PS……..]**

**(PS):** Esas cosas se pueden ver, porque ahí no tenemos, el tiempo para llenar formularios, aprender y que está esperando el otro paciente los treinta minutos afuera, y si llego diez minutos atrasado, veinte minutos,

**(PS):** Yo encuentro muy bueno quizás esto de ir a la casa, de hecho, es bueno, tienen mejores resultados, pacientes quizás mejoran la adherencia, uno puede observar factores, pero a la vez, internamente siento ¡Rabia!**,** ¿Por qué? Porque esa es la actitud paternalista, esa es la actitud de… uno llega a la casa ¿por qué el paciente no se preocupa de venir para acá?”, cuando le vamos a dar quizás las mismas indicaciones que le vamos a ir a dar a la casa.

**[Todos: “No es lo mismo” murmullo]**

**(PS):** El concepto es lo mismo

**(PS):** claro, es similar. La información es la misma

**(PS):** El concepto es “cuando tú te acercas a él, es como la gratitud de partida permite que se abran un poco más las puertas”

**(PS):** En el sentido de que agradece que uno los vaya a ver, que se sientan acompañados, entonces, obviamente es como: me siento privilegiado que ustedes vengan acá y me permitan… y te permite…

**(I)** ¿y la relación que se establece con el paciente?

**(PS):** es cercana se sueltan más, hay más confianza te muestra su hogar porque el abrirle las puertas a uno, del hogar de ellos a uno, ya es un signo de confianza entonces además entran en confianza, es verdad la visión es diferente pero la información como dice aquí mi compañera es la misma, uno observa otras cosas por supuesto…

**(PS):** Porque ves que el paciente cambia su forma de ver las cosas

**(PS):** porque a lo mejor, también, estamos muy médicalizados, por ejemplo, yo estoy detrás de un escritorio, ya, estoy detrás de un escritorio y hay una distancia

**(PS):** otro y aparte que uno está mirando al computador, al paciente, al computador al paciente, entonces ya obviar eso y mirarlo a los ojos hace falta

**(PS):** Yo creo que la gran diferencia es cuando estás en la casa, porque en general todos preguntamos, no sé, ¿qué es lo que come?, ya, ¿cuánto?... entonces yo creo que la gran diferencia es que cuando estás en la casa, como decía el colega, cual plato es en el que usted come, entonces el caballero va a decir… porque es la percepción de él y nosotros escribimos lo que te dice el paciente y no tenemos por qué dudarlo, pero cuando estas allá las cosas cambian porque son visiones distintas, y yo creo que es la gran diferencia de que el paciente venga acá cuando debiera tener más confianza, sí, que también hay un tema paternalista, ¡**sí!,** pero lamentablemente es buscar alguna solución al tema y yo creo que si eso es necesario, si mejora, me agrada mucho la idea de que, por ejemplo, nutrición pueda ir a la casa y que vea la cocina

**(PS):** Pero ojo…

**(PS):** Y que diga ponte tú no sé, la misma cuestión del arroz esa cuestión… buena, ¿cachai?, pero en general acá tú le deci eso a un paciente va a llegar a la casa y se va olvidar de todo, porque no va a saber cómo asociar eso allá en la casa

**(PS):** Ahora ojo, lo que nos pasó con una visita a terreno, un paciente hipertenso, perfecto, llego vamos a revisar la cocina, vamos a ver que herramientas tiene como para poder dentro de los pocos recursos que tiene como optimizarlo, “es que no tengo nada”, perfecto, no tiene nada porque no tiene plata y no ha comprado nada pero hay millones de otras cosas que podríamos haber visto, ¿cachai?, tiene caldos maggi, que tipo de… millones de cuestiones, pero también, existe el rechazo, por ejemplo, porque al abrir tu cocina significa que tú también te vas a responsabilizar de lo que hay dentro de esa cocina, que no es culpa del mundo que ella este gorda, que no es culpa del mundo que tenga los triglicéridos elevados que es de ella, y eso es súper difícil, entonces a mí me parece una instancia súper buena y tal vez la podríamos hacer ¡al ingreso de un paciente diabético!

**(PS)**: Eso sí estaría bueno…

**(PS):** Y de ahí dejarlo en los controles porque enganchas con tu equipo y generas un lazo que es mucho más cercano, pero hacerlo permanentemente y cuando ya estén muy descompensados no tiene mucho sentido porque tenemos que prevenir eso, porque nosotros somos muy de resolver el problema cuando ya está grave, medicalizarlo, no prevenir, y nuestra pega en APS es la prevención, y ahí es el proceso que nosotros tampoco estamos cumpliendo, ¡¡de prevenir!!, de hacer que esto suceda cuando el niñito está gordito, y cuando la abuelita le está dando tres litros de bebida a ese niñito ¿Por qué? Porque a los ocho años va a ser diabético ¿cachai?

**(I)** ¿dime?

**(PS):** ahora quiero volver a la parte del ejercicio, porque resulta que si tú le indicas ejercicios a una persona lo más probable es que no los haga, ya, eemm… pero lo que hemos visto, por ejemplo, con los pacientes que tienen artrosis, ya, tenemos hartos pacientes con artrosis y casi todos son hipertensos o diabéticos, o tienen las dos cosas, entonces en la sala de rehabilitación los pacientes venían y venían y querían terapia, kinesioterapia, y querían kinesioterapia, y entonces nos saturamos de horas de médicos para mandar al kinesiólogo, por eso se creó un taller, un taller para el adulto mayor que lo imparte un TENS, un auxiliar paramédico, ya, y ese es para resolver todos los pacientes policonsultantes en la sala de rehabilitación, pero que querían seguir haciendo ejercicios, si esa era la idea, ellos querían venir a hacer ejercicios y estaba esa instancia entonces el… el… TENS, entonces, tiene un taller que es súper efectivo, la gente viene, hay un grupo de veinte o treinta pacientes que vienen regularmente y van a crear como un… van a sacar la personería, ya, y a la par están ingresando nuevos pacientes que se están incorporando y que van hacer otro grupo, ya, entonces, ¿qué es lo que ellos quieren?, quieren tener un lugar donde hacer ejercicios, una… una… espacio regular de ejercicios, dos o tres veces a la semana, quieren juntarse con otras personas a juntarse hacer el ejercicios

**(PS):** Pero Paz, ¿qué edad tienen esos pacientes?

**(PS):** Son todos adultos mayores, son casi todos adultos mayores

**(I)** ¿entonces tú dices por la edad, porque no están trabajando ya? como están en su casa

**(PS):** sí… no, no, pero te digo, depende del tiempo, yo no estoy poniéndome con los diabéticos jóvenes

**(I)** ¿vienen igual?

**(PS):** sí, vienen, sí, vienen, entonces, está la instancia esa como de ejercicio más compartir, ya, no hacerlo solo, compartir, entonces si tú… creo no hace muchos años hubo también unos talleres

**(PS):** No, era el taller de cardio que dejaron de hacerse

**(PS):** Taller de cardio que dejo de hacerse y todavía la gente extraña el taller de cardio…

**(PS):** Pero si uno la cita no viene po

**(PS):** A ver, por decirte el mismo programa vida sana ¿cierto?, el programa vida sana involucra a las personas que son diabéticas, que son hipertensas, y con sobrepeso, con obesidad, o pueden ser, también, intolerantes a la glucosa…

**(PS):** Síndromes metabólicos

**(PS):** Síndromes metabólicos, incluye actividad física y en la totalidad de los pacientes que están inscritos en vida sana van desertando, la minoría van a hacer actividad física y ahí uno se percata, porque tienen acá a Álvaro que hace el taller, personalizado, todo y no… tienen ese recurso y no vienen

**(PS):** Yo diría que es un treinta por ciento de los pacientes que ingresan, de hecho es mucho, veinte por ciento de los pacientes que ingresan en ese programa que terminan haciendo actividad física, ahora los que terminan haciendo actividad física bajan 10 kilos en el año o sea, ¡sí, tienen un alto impacto! pero no todos, los adultos mayores vienen porque claro están solos en sus casas, muchas veces viven solo, y en el último minuto que tienen para compartir es acá o sea vienen y de repente nosotros no podemos escucharles porque te cuentan toda su vida en esa media hora o veinte minutos y es súper difícil hacer la intervención porque no les interesa escucharnos lo que nosotros tenemos que decirle

**(I)** ¿Qué es lo quieren?

**(PS):** ¡quieren ser escuchados, nada más!

**(PS):** Pucha si hoy en día, ni siquiera solamente los abuelos, si el gran problema hoy en día es que nadie habla

**(PS):** Nadie se escucha

**(PS):** Nadie se sienta a almorzar y decir “¿oye que te pasó hoy día?” y los abuelos, menos todavía si ya soy viejo

**(PS):** No los pescan

**(PS):** Habla siempre lo mismo y eso es terrible, es terrible si ustedes se ponen a analizar hoy en día cuantos clubes de ancianos existen, muchos, pero son totalmente insuficientes, si ustedes se dan cuanta, si ustedes analizan y le preguntan a un abuelo: ¿qué? ¿Cómo lo paso ayer en el club?, ¡¡espectacular!!; y las viejas bailan y salen a caminar y esperan el día, ¡lo esperan! **[Todos exclaman de felicidad**] entonces, yo les iba a preguntar ustedes pensaban que si los adultos mayores no hacen ejercicio, o si lo hacen, lo hacen en grupos, es netamente porque se sienten solos, y eso lo podemos asociar no solamente a la actividad física, sino también, a la ingesta, porque a lo mejor lo que quieren hacer es que como a toda la familia se… está con cuidado porque yo soy diabético, que yo no me estoy tomando los medicamentos voy a lograr que mi hija me pesque, ¿cachai?, a lo mejor es eso pero tampoco lo hemos visto, lo vemos solamente del punto de vista patológico, pero del punto de vista emocional como tal pero recordemos que también la parte emocional entra, y también, la dejamos de lado, hoy día claro todo es tecnología espectacular a todos nos sirve, oye pero recordemos que hablamos más por teléfono, chateando, porque no me atrevo a decírtelo a la cara, ¿y cómo le explicas eso a tu mamá?, ¿cómo le explicas eso a tu abuela?, ¿cómo tú le explicas entonces que te tienes que tomar esa pastillita? ¿por qué? porque te va hacer bien para esta enfermedad, porque con esto voy a evitar que te amputen una pierna, si no tenemos la capacidad esa…

**(PS):** pero hay otra cosa po, es que no es solamente en las casas, aquí también, porque cuando viene hablar contigo “sí, señora sí, pero dígame cuanto está comiendo”, “sí, sí, pero señora dígame, respóndame todo” “sí, pero señora por favor”, ¿en qué momento te escucho?, entonces esperaste cuanto tiempo para una hora

**(PS):** Y para que yo venga y te de los treinta minutos y ojalá que sea menos porque así me puedo poner al día con la ficha anterior

**(PS):** Y hay que empezar a registrar todo

**(PS):** ¿Cachai? porque o sino después me van a auditar “oye es que no le hiciste esta cuestión”, entonces cómo, el tema es, ¿cómo tu cumples? Yo no estoy en contra de que se tenga que llenar tantos formularios, me parece espectacular, porque una de las formas que también nos dicen que a lo mejor debemos tener una mejor atención, pero estamos perdiendo lo básico que es recordar que somos seres humanos y que para recordar que somos seres humanos hay que comunicarse, ni siquiera eso tenemos y hablamos de calidad

**(I):** ¿como se comunican entre ustedes?

**(PS):** eeh….

**(PS):** cuando se puede

**(PS):** tenemos reunión

**(PS):** Sí, tenemos hartas instancias de comunicación

**(PS):** es que, es... es el tema, yo creo que siempre están las instancias es decir “¿compañera pucha nos podemos juntar hoy día?”, “ya”, te llega un paciente atrasado, “oye déjame terminar con esto voy al tiro”, oye pasan semanas que nos quedamos de juntar... Y ya saliste a colación.

**(PS):** Optas por el mail o el WhatsApp

**(PS):** y si, no vas a salir a colación para seguir hablando de pega, porque si te pones a pensar en realidad que es lo que todo conversan es trabajo, entonces, en qué momento te llegas a desconectar, entonces yo he cachado que muchas veces se dice “párale”, “hablemos otra cosa”, ¿Cachay? Entonces, pasa todo esto que nadie habla, yo creo que llama la atención, necesitan que los escuchen, también, nosotros necesitamos comunicar, pero como tú tampoco me escuchas, tampoco tengo porque hacerlo, pero insisto yo creo que esto es un tema social y como vamos a cambiar eso, no lo sé

**(PS):** yo creo que el puente es el tiempo

**(PS):** Pero o sea ¿Cómo formas el puente para que te den más tiempo?

**(PS):** Por ejemplo, a mí me pasa, logro hacer el switch con el paciente, logramos mirarlo en el mismo punto, ¡tengo que hacer esperar a la señora que me está esperando afuera! o sea, ¡¡sorry!!

**(PS):** Pero te arriesgas a que te pongan un reclamo, también

**(PS):** A mí me da lo mismo, pero es ese proceso que no puedes perder, pero a que costo, de lamentablemente arriesgarte o no cumplir con el rendimiento.

**(PS):** totalmente de acuerdo, si el tema es…

**(PS):** Y no alcanzaste a cerrar las fichas

**(PS):** Y el atraso, cerrar fichas y…

**(I):** Estamos agradecidos de todos los que participaron, de todos lo que compartieron con nosotros, si alguien quiere aportar algo, una palabra de cierre, lo que quiera, está totalmente abierto.

**[Se ríen y algunos agradecen la instancia]**

**(PS):** bueno gracias a ustedes, también por hacernos participe, y en lo que lo podamos a ayudar aquí nos tienen.

**(PS):** Muchas gracias

**(I):** No a ustedes

**(PS):** yo solamente quería decirles, darles como una sugerencia, si, eeh… en alguna oportunidad participé en una investigación que se llamaba “desviación positiva” en salud con un… ¿no sé si conocen el termino de desviación positiva?

**(I):** No, no lo conozco

**(PS):** ya, La desviación positiva estudiaba, por ejemplo, comunidades no se po un barrio, que tuviera las mismas condiciones socioeconómicas, que tuviera los mismo acceso a la… a parques, eeh… un barrio con las mismas condiciones socioeconómicas, ya, y estudiaban a las personas, por ejemplo, por qué habían mamás y que los niños estaban tan bien cuidados, tenían menos problemas de salud y habían, otras mamá, que los niños se enfermaban a cada rato***,*** que se yo, entonces, buscaban los puntos o las características de la familia donde los niños no se enfermaban y las características en la mamá de los niños que se enfermaban más, entonces esas, esas… cuales era las condiciones de porque estos niños no se enfermaban y siendo que vivían en las mismas casas, los mismos medios…

**(I):** o sea, esas variables estaban controladas y las otras eran las que podían hacer la diferencia.

**(PS):** claro, buscaban,… se llamaba desviación positiva, por ejemplo, obviamente una de las características era el nivel educativo de las mamás, te fijas, entonces en esto yo creo que es lo mismo, ya, porque nosotros tenemos, por ejemplo, acá “Las Araucarias” que es un barrio que tenemos aquí al lado, ya, si nosotros vemos, por ejemplo, que familias en donde si el nivel educativo del cuidador influye mucho en… por ejemplo, que haya problemas de alcohol o drogas también influye, influye las redes, si las redes son buenas sino no son buenas influyen y yo creo que en la parte de diabetes es lo mismo, ¿por qué hay pacientes que vienen compensados y otros por qué no vienen compensados?

**(I):** nosotros estas vez específicamente en esta investigación, estamos buscando entender… estamos viendo a la carga de tratamiento y la capacidad real que tienen el paciente aquí en Chile de asumir esa carga y la percepción que tienen ustedes, así que les vamos a contar todo lo que pase. Un millón de gracias.

**(PS):** No, agradecer a ustedes pues.

**HEALTH CENTER 3- HEALTH PROVIDERS**

**Investigador** **(I):** Ya, voy a dejar la grabadora aquí al medio. Agradecerles eh que hayan querido participar de este grupo focal eehm sabemos que tenemos nutricionistas, enfermeros y médicos. Estamos felices, y queremos partir eh abriendo este grupo focal con una pregunta antes: ¿cómo perciben ustedes o qué perciben ustedes, ¿qué pasa con los pacientes cuando uno le entrega el diagnóstico o asume un diagnóstico tentativo de enfermedad crónica/diabetes? Cómo reaccionan, cómo los ven… Está bien… Sí…

**Proveedor de Salud** (**PS**): Hay pacientes que lo aceptan con mucha tranquilidad, pero la mayoría de ellos no lo llegan a aceptar eh y siempre yo he visto la necesidad de que esos pacientes que no aceptan ir a ayudarlos con algo de psicología al inicio, porque psicológicamente para que acepten su enfermedad. Incluso algunos se ponen a llorar, otros se ponen molestos eeh y la mayoría son así, no aceptan.

A pesar de su… de su desorden, porque la enfermedad la adquiere uno y sin embargo con el desorden y los malos hábitos eeh pero a pesar de ello ellos no.

**I:** ¿Cómo lo ven ustedes?

**PS:** Sí, yo concuerdo con la doctora eeh me han tocado muchos pacientes que, como lo relata ella, eh tienen un problema emocional muy grande al sentir la… sobre todo lo he visto mucho en hombres que, al ser declarados diabéticos, se les viene el mundo encima. Un un shock emocional bastante fuerte, casi como una depresión, como para para la enfermedad, para el tratamiento en sí de la enfermedad. Entonces, yo concuerdo con la doctora que debería tener un tratamiento psicológico de por medio también importante, quizás dentro de las derivaciones del cardiovascular, estar apoyado también con un tratamiento psicológico. Sobre todo, quizás en el inicio, después ya, a largo tiempo, eh podría tener mmm otro otras derivaciones, otro tipo de… pero al principio, en la primera parte, debería tener un apoyo psicológico.

**PS:** Ante todo los pacientes diabéticos, porque ellos lo relacionan con la gente con poca información o de pronto con falta de educación, eeh ellos lo relacionan con… sí, la diabetes: amputación, porque lo han visto.

**I:** ah…….

**PS:** Sí… Entonces el hipertenso de pronto no, el XXXX… (2:25) más suave. Pero más desde el diabético, porque dice: a mi vecino lo amputaron a mi mamá murió con una amputación…

**PS:** Con la insulina también…

**PS:** Claro, también le tienen terror a la insulina…

**PS:** Muchas veces a mí me… me ha tocado escuchar el término que usan los pacientes que tienen la diabetes buena y la diabetes mala. La diabetes mala es la que se inyectan insulina, el insulino- requirente, entonces como que igual falta un poco de información en ese sentido y también el término como de… ¿enfermedad crónica? Yo siento como que a algunos pacientes no les queda muy claro porque hay gente que me dice: no, yo fui hipertenso, pero ya me sané. Entonces varias veces me ha tocado escuchar eso y hay que explicarle a la persona que una vez adquirida la enfermedad es para toda la vida, pero que se puede mantener controlado o no. Entonces igual, no sé si falta información eh es por el tipo de población quizás que nosotros atendemos de escasos recursos, baja educación puede ser pero me ha llamado atención eso, como que no aceptan una enfermedad o no entienden el concepto de cronicidad de la enfermedad. Eso ha sido mi…

**PS:** Claro, por ejemplo, también cuando el paciente acude, uno se le pregunta, a los controles, ¿y usted qué es? Hipertensa, sí sólo hipertensa, diabética no y lo hacen mucho hincapié al diabético, no doctor.

**PS:** Como que le toman más gravedad a la diabetes.

**PS:** Entonces como que a la hipertensión no la toman también como un asunto… una enfermedad crónica y que hay que controlarlo.

**I:** Tú ibas a decir algo.

**PS:** Ah, no lo que pasa es que… al momento del diagnóstico por lo general empiezan, así como… Pero qué raro yo casi no consumía azúcar, entonces ellos todo lo asocian como que diabetes es azúcar, pero no que hay alimentos que se transforman en azúcar. Entonces hay también eh de repente pasan mucho tiempo los pacientes con ese concepto, entonces pasa que ellos, claro, ellos dejaron el azúcar, ya no comen pastel, pero solamente pasteles y para eso ellos es que no están consumiendo y se están cuidando… No que tienen que cuidar los carbohidratos ni otra… la parte de la alimentación. Ellos como que solamente dulce.

**I:** O sea……

**PS:** A mí me ha pasado mucho de que por ejemplo hay pacientes que son… No sé, que han tenido historia familiar de diabetes o de hipertensión, pero más que nada en diabetes y que dicen: mi mamá no sé po murió de 90 años de diabetes y que nunca ha tomado ningún medicamento nunca se XXXX (4:53) ni nada, entonces ellos piensan que es el tratamiento el que les hace más daño que la enfermedad en sí. Entonces muchos pacientes son XXXX (5:02) a tomarse los medicamentos por eso o piensan que si no comen como decía acá la Ignacia no comen azúcar ellos van a andar bien no van a necesitar nada más, entonces hay mucho de creencias y de mito en relación con la enfermedad, sobre todo con la diabetes yo veo…

**I:** ¿es independiente de la educación?

**PS:** Algunos sí… Hay muchos mitos en cuanto alimentación, sobre todo que ahora… No sé po, la tele **PS:** Yo creo que debe educarse tanto al paciente como a la familia, que por ejemplo he tenido experiencias en pacientes que tienen un nivel de educación medio y que, con el apoyo de la familia, superan… Superan mucho, pero el grado de tristeza o de aceptación… El problema es la aceptación de la enfermedad.

**I**: Exacto. ¿Hace diferencia que el paciente sea hombre o mujer?

**PS:** O sea, en la alimentación las mujeres cocinan, entonces ellas por ejemplo en la hipertensión ellas saben con menos sal entonces, pero el hombre no cocina entonces por lo general prepara la esposa, prepara… La sobrina, prepara la hija y XXXX (6:14) la hija no tiene un conocimiento mayor de la enfermedad entonces le agrega sal y él se tiene que comer la comida que hicieron.

**PS:** Generalmente también los… No que también XXXX (6:26) diferencia de… También por un tema bastante como XXXX (6:30). Los hombres muchas veces no se preocupan de una enfermedad o de un medicamento, se los tiene que dar la esposa… Lo mismo el tema de la alimentación como dijo la XXXX (6:39), entonces hay diferencia entre hombre y mujer los hombres actualmente el hombre y por cultura no se hace cargo tanto de su enfermedad como lo hace la mujer. Nosotros sabemos que hombres mueren más que mujeres, también puede ser por lo mismo, entonces también falta como… complicado cambiar ese ese como… Como… Debería considerarse, claro, también muchas más mujeres van a actividades de como autocuidado que uno, muchas también más mujeres van a actividades de adulto mayor que hombres, ese XXXX (7:09) adulto mayor hay más mujeres que hombres y muchos son diabéticos. Entonces en ese aspecto hay harta diferencia.

**PS:** Sí, bueno eh con relación a lo mismo eh siempre se ve claro que la mujer se cuida más porque de hecho el ciclo vital la mujer tiene mucho más control la verdad hasta con el embarazo todo, entonces tiene como la costumbre de cuidarse más y en relación con la alimentación es más fácil, o sea, no es que cueste… Es más fácil que la mujer empiece a hacer un poco cambio de hábito, y a disminuir porciones en relación con los hombres, los hombres siempre comen mayores cantidades y les cuesta mucho más como asumir esto de ir disminuyendo las porciones que no tienen que comer esto que no pueden comer esto otro, ¿ya? Y eso es uno cosa… Y lo otro que he visto es que eh muchos pacientes actúan como con… Con relación al miedo que les provoca la enfermedad, cuando le pasan el diagnóstico, tienen el diagnóstico vienen como por ejemplo a la consulta vienen como… Más incentivados a lo que uno le enseñe y todo, pero va pasando el tiempo y se van relajando hasta que ocupan insulina y ahí, de nuevo, empiezan como con nuevo ciclo y entonces como que… Eso me pasa a mí.

**I:** ¿Qué es lo más complejo?

**PS:** O sea, que se organicen con sus horarios es una cosa tremenda, que coman las porciones que corresponden que empiece a hacer actividad física, eso es…

**I:** ¿Por qué creen ustedes que pasa eso?

**PS:** Yo creo que lo que pasa es que… La el origen de la enfermedad es eso po, son tus hábitos de vida, vida sedentaria, comer con mucha sal, comer con mucha azúcar y es donde hay que atacar ahí mismo, no es suficiente empezar a indicar fármacos si los estilos de vida no van a cambiar entonces, a mí me pasa de repente en 30 minutos, claro, uno ve los exámenes, ve las complicaciones derivación necesario, indica los fármacos, los ajusta, pero de ahí a entrar en detalles de cómo vivir tu vida y cambiar tu forma de ser en 30 minutos no se da esa magia… Yo creo que en la orientación en general es buena el programa esto de multidisciplinaria, seguimiento crónico, pero faltan talleres encuentro yo, promoción primaria secundaria, prevención como que ahí está la el tema, talleres de 90 minutos y lo de comunidad también, si estamos basándonos en mitos y en cosas que pasan a las casos extremos, se pueden ocupan a veces si están tienen miedo, pero la gran mayoría de las veces no son la realidad po, y no apunta para allá el enfoque del tratamiento tampoco, no se trata de andar asustándole a la gente que le vamos a amputar un pie y por eso no puede comer más azúcar.

**I:** Ok

**PS:** Porque la mayoría trabaja fuera de la de la de la comuna, entonces eso significa que ellos tienen un un trayecto a su trabajo relativamente largo en donde no, van parado muchas veces, entonces no tiene la oportunidad de comer, una vez que llegan justo, llegan a trabajar, los horarios de colación también son más más extendidos, llegan cansados entonces ejercicio no tienen ganas de hacer ejercicio, depende mucho las mujeres llegan y llegan a hacer las cosas de la casa eeh o el hombre a veces tiene dos trabajos, entonces es como súper complicado para ellos como más los trayectos y el trabajo y el compatibilizarlo con la alimentación y con lo de hacer ejercicio.

**PS:** Yo creo que igual va un va por un lado de educación… Sí, porque o sea si yo lo llevo a mí que yo también trabajo tengo hijas, hago las cosas en mi casa, pero igual voy al gimnasio y me cuido entonces creo que igual va por un lado como educación eeh más que nada, como un poco de preocupación de prevenir o de mejorar quizás en la condición.

**I:** Pero tú dices educación …

**PS:** No sé si falta de educación, si no que es como falta de… De entender que es como como que yo siento que el paciente que dice: ah sí ya entiendo y se quedó ahí, pero no es como que él no es como… Claro, como visionario a el prevenir o a o a como mantenerse estable en la condición que está o cambiar quizá, cambiar…

**PS:** Es que hay que considerar el tema de… La edad a la que el paciente se le diagnostica, claro, porque es distinto un adulto joven a un adulto mayor, o sea tiene más tiempo realizando una misma conducta y cambiar esa conducta que XXXX (12:04) por mucho más tiempo es más complicado, más difícil, por lo general el paciente adulto mayor que se le diagnostica diabetes, tiene artrosis, o sea, decirle vaya a caminar 30 minutos al día, no lo va a hacer eeh y sí el tema de del sexo es importante porque el hombre de partida el hombre está acostumbrado a que, si la esposa le cocina con poca sal porque es diabético es hipertenso le pone sal en la mesa y con eso le hace más daño que comprándola ¿ya? Entonces es un tema de costumbre, un tema de hábito, un tema de creencia, para y para iniciar el tratamiento primero uno primero que nada tiene que preguntarle las creencias, cuáles son sus creencias qué es lo que usted cree de la enfermedad, qué sabe usted de la diabetes y ahí irlo orientando y otro que nos juega en contra es el tiempo en que uno le hace los controles, porque si estamos hablando de una persona que ha llevado 50 60 años realizando hábitos pensar en que con 30 minutos que no son efectivos los 30 minutos cuando es XXXX (13:04), cada 6 meses cada 3 meses no se va a cambiar, entonces el paciente de aquí puede salir motivado, pero es tan largo el tiempo en que va a venir al control que dice o si no, por a o b motivo hay que cambiarle la hora, una el paciente no puede venir de pronto otra porque el profesional no pudo llegar por a o b se enfermó lo que sea, entonces el tema de llamar al paciente y decirle: hoy día te tengo que cambiar la hora para tal fecha, entonces el paciente dice: a mí me está costando y si mi médico no se preocupa, entonces de pronto él puede sentir que un poco muy poco el interés que uno le dedica a ellos. Eso…

**I** ¿Y te plantean de repente los pacientes o no? Que les cuesta venir o que a lo mejor les cambian las horas…

**PS:** Hay gente que le gusta les gustaría venir más, hay gente que le gustaría venir menos y ahí es variable, o sea, depende el gusto del consumidor.

**PS:** Si hay gente que le gustaría venir en otro horario, por ejemplo, después de las 5 si es que trabajan o un sábado en la mañana, y no siempre tenemos la disponibilidad de hacer vespertino, entonces eso también complica.

**PS:** O… Me ha pasado también que hay gente que sólo considera importante el control con médico, por ejemplo, porque nosotros trabajamos entre 3 profesionales médico, enfermera nutricionista y según eso es ciclo según su riesgo cardiovascular y hay personas que dicen: ¿Y para qué voy a ir a la nutricionista? Si ya sé lo que tengo que comer o por ejemplo ¿y para qué voy a ir a la enfermera si me pesa y me mide y eso lo puede hacer el médico también? Entonces como que no le ven el enfoque como… integral… y multidisciplinario.

**PS:** Es que tampoco está como…

**PS:** Lo que pasa es que vienen como vienen como a la consulta con el médico, pero todavía no logran interiorizar que es control cardiovascular, sino que vienen con otras problemáticas como además XXXX (14:50) y quieren que el médico también solucione su otra morbilidad.

**I:** Cosa que el resto de los profesionales no podrían hacer…

**PS:** Porque quieren medicamentos…

**PS:** Y medicamentos hora al médico… Entonces ellos… Muy importante para ellos los medicamentos…

**PS:** Claro, quieren venir de hecho se saltan los otros controles no, o sea, teniendo la hora del nutricionista o de enfermera, no llegan, se saltan horas, se las saltan no más y vienen al año después con el médico, por supuesto con la glicemia la XXXX (15:18) súper descompensada, entonces… Tal vez es es un tema de… De educación este el decirle al paciente cuál es la función que cumple cada uno, porque la enfermera está… La función de la enfermera es básicamente educación ¿ya? Y la nutricionista el tema de la alimentación, o sea, el paciente no tiene concientizado eso, entonces como que engloba todo en una sola cosa y, como el doctor es que el me va los medicamentos, con los medicamentos yo me voy a mejorar, voy donde el médico y por supuesto a nosotros, a nosotros se nos carga porque uno va con una carga enorme a… Saliendo de aquí uno va con una carga enorme de ver que por más que uno hace, el paciente sigue con la glicemia alta, sigue con la presión alta y y muchas veces es responsabilidad de ellos.

**I:** Tú ibas a comentar algo…

**PS:** Ah, sí, yo quería comentar que el tratamiento cardiovascular no está hecho a la medida para todo estándar, o sea pacientes hipertensos, diabéticos, riesgo alto, control cada 3 meses y da lo mismo si tú quieres o no hacerlo eeh te van a tocar igual y para los dos lados, o sea si el paciente no quiere venir, no va a venir, da lo mismo lo que uno haga y al revés, si uno quiere tratarlo antes tampoco puede hacerlo. Y ese es el problema es demasiado rígida la estructura del programa como para que… Fuera hecho a la medida, por ejemplo, de salud mental es totalmente distinto, ahí uno elige quiero que venga el psicólogo, ¿el psicólogo va a entrar ahora o va a entrar en 4 semanas más cuando esté más despierta? Eeh, lo voy a seguir en 6 semanas, lo voy a seguir en 8 semanas, lo decide en 3 meses o cada 6 meses y ahí la cuestión cambia…

**PS:** Los profesionales deciden el control…

**PS:** El criterio clínico es mucho más crítico que una cuestión estándar, no… Esa… Está bien que no tengan cómo tenemos que hacerlo, ok, lo mismo, es estándar, pero cuando ya uno quiere hacer las cosas como corresponden no se puede.

**I:** ¿¿¿Aplicar el criterio profesional???…

**PS:** Claro, cuando uno quiere aplicar el criterio profesional que nos enseñaron, no se puede…

**PS:** Hay calendarios que son ministeriales que vienen por orden del Ministerio que hay que hacer cerca de determinado tiempo y localmente se modifican también…

**I:** ¿Ya?

**PS:** Entonces no hay una estructura… Lo que pasa es que, si se modifica localmente, esa modificación local también es rígida, ¿te das cuenta? Va para todos. O sea, no sólo el Ministerio te dice que… No solamente el Ministerio pone la rigidez en el tema de programa sino localmente se pone…

**I:** Ya …

**PS:** O sea, si tú quieres ver al paciente para convencerlo para que el paciente entienda su problemática, su patología para que la entienda, lo vas a ver mensualmente hasta que el paciente se convence, pero no se puede porque localmente te dicen que no, no hay horas disponibles.

**I:** Oye, y en el asunto de los medicamentos… Los pacientes les dan un valor grande a los medicamentos, ustedes decían que el médico es capaz de prescribir y todo, o sea ¿con los medicamentos se cumple?

**PS:** No.

**PS:** Tampoco son tan adherentes. Yo encuentro de repente que le dan demasiados medicamentos, entonces uno no se toma el de la tarde, porque son muchas. Claro, o para que no se le olvide, se toman todo junto en la mañana.

**PS:** O sienten que se le subió la presión y se toman otra pastilla para la presión por si acaso. O comieron más y se toman otra metformina.

**PS:** A mí me pasa que los medicamentos también tienen reacciones y también le afectan, entonces les empieza a doler la guatita y pum, dejan de tomar el medicamento. Entonces eso a veces no lo pueden, eso no lo conversan antes no lo conversan en XXXX, a lo mejor para ver un cambio de medicamento, algo así y ellos se niegan y lo dejan de tomar. O o pueden llegar a tomar otro medicamento que le prescribió alguien más… Puede ser también…

**PS:** Sí me ha pasado…

**PS:** Porque entre ellos se intercambian sus signos y síntomas en respuesta al tratamiento, no, ellos dicen: a mí me hace bien… Se intercambian, así de sencillo…

**I:** ¿y la posibilidad de los pacientes de hacerse cargo de su salud física?

**PS:** adultos mayores y uno… No salen a caminar mucho, otros están a cargo de los nietos, cuidan niños… Entonces de repente están como a la tarde, 7 de la tarde cuidando niños, entonces eso también le les complica un poco porque no cuidan el nieto, sino que son 2 3 nietos.

**PS:** Pero hay buenos pacientes que asisten acá al ejercicio de adulto mayor y que funciona súper bien, pero los cupos son limitados también.

**PS:** También.

**I:** Ah, o sea si hubiera más cupos ¿??…

**PS:** De todas maneras.

**PS:** Porque algunos vienen incentivados, esperando que poder hacer ejercio acá porque en las comunas hacen, pero por tiempos limitados, no sé, 4 meses y después se acaban, entonces no hay algo que ellas puedan seguir por todo el año. Entonces quieren asistir acá por lo mismo, pero los cupos limitados de repente no alcanzan.

**PS:** yo creo que la alimentación de partida tiene un factor económico importante porque muchas veces nosotros le exigimos a los pacientes que consuman, por ejemplo, tanta cantidad de frutas y verduras al día. Imagínese en una casa donde viven siete personas, el gasto económico es bastante alto por día, entonces también les afecta. Es mucho más fácil comprarse una sopaipilla o comprarse un completo que comprarse no sé un plato de comida que tenga arroz integral, que tenga harta verdura, es mucho… es muy distinto. Eeh bueno, tampoco se ofrece esa posibilidad a nivel social, ¿ya? Eso también debería ser otro tema, pero influye bastante…

**PS:** Yo creo que depende del sector. Ya, por ejemplo, en mi sector, yo siento que mi paciente como que se alimenta bien, pero sí hace gastos en cosas que no debería, pero yo siento que no, no hay como, no siento que el paciente coma mal, se alimente mal, sino que quizás la forma o la selección de alimentos puede que varíe, pero el paciente generalmente come, por ejemplo, pescado, vacuno, es como súper variada la alimentación. En un paciente de mi sector.

**I:** Ya, ¿y el resto?

**PS:** Yo siento que, a ver. Siempre cuando uno le hace la consulta eeh evalúa la eh cómo está el estado socioeconómico del paciente. No le voy a estar dando ciertos alimentos que nunca ha consumido, sino como los básicos. Ehm, pero en general eeh tienen como disposición de comprar ciertos alimentos, ya, o sea, uno le va a decir compre coma ensalada. No tiene que comprar la ensalada más cara, compre lo que haya disponible, lo que usted pueda obtener y lo mismo pasa con la fruta. La temporada, que sea más barata. Pero los pacientes también eh no eligen de repente muy bien sus alimentos. O sea, teniendo los recursos, ellos se compran la Coca Cola o le compran la galletita chiquitita al hijo en el supermercado que, claro, a lo mejor le vale $120.000, pero eso mismo podría ocuparlo para comprar fruta. No tienen…

**PS:** Comen harto pan y arroz.

**PS:** Es imposible sacar el pan.

**PS:** Es que yo creo que eso es estructural. Es mucho más barato comprar masas que comprar carne, entonces en nuestro sector, nuestros pacientes, pacientes adulto mayor con pensión, FONASA A, ¿le vas a decir compre pescado? O sea, tú, para uno mismo al… Yo estoy viviendo por acá cerca y te digo yo voy al supermercado acá y no hay pescado, hay carne de mala calidad, hamburguesas, entonces también es estructural como… La gente no… Es más caro, no lo compra, no hay oferta tampoco del lugar y eso es mucho más grande, no es de nosotros poder, es salud pública ya.

**PS:** Sí, pero acá en la comuna también tenemos hartas ferias libres.

**PS:** Eh claro, y también hay como… es verdad que la fruta no es tan barata, tampoco la verdura no es tan barata. Que los cereales, el arroz, el pan también, pero también es una cosa de que sí, la persona puede comer pan, pero tiene que comer menos cantidad. No es que no pueda. Sí puede comer arroz todos los días, si es que no tiene más disponibilidad, pero no se va a comer tres tazas de arroz.

**PS:** Yo creo que el problema es las porciones más que la disponibilidad.

**I:** ¿Y el tema del peso?

**[HABLAN DE MANERA SIMULTÁNEA]**

**PS:** Es como de todo el ciclo vital. Desde el embarazo, los niños, los adultos, hay mucho sobrepeso y obesidad.

**PS:** Existe todavía el concepto de que gordito está más sanito, entonces, de hecho, hay niños que están normales y las mamás vienen súper preocupadas que el niño está flaco y que la vecina le dijo que estaba flaco. Entonces, entonces de ahí ya viene parte de…

**I:** Existe prejuicios, de…

**PS:** Y también va de la mano que no está contemplado como enfermedad, es muy poca gente que venga “oye, estoy gordo”. Eso no es un motivo de consulta, no hay un plan para ver la obesidad y debería haberlo.

**I:** ¿Y hay conciencia de problemas de peso?

**PS:** No, ellos tienen como “estoy un poquito pasado, pero no tanto” …

**[HABLAN DE MANERA SIMULTÁNEA]**

**PS:** No tienen conciencia, como el peso extra que tienen y los problemas que lleva la obesidad. Es falta de educación, falta de educación en los colegios, como de ahí partimos mal.

**I:** Si de ustedes dependiera hacer algunos cambios ¿qué cosas creen ustedes que ayudarían?

**PS:** ¿Adherencia en general o adherencia a qué?

**PS:** Porque adherencia a fármacos, uno podría dejar la receta eterna y los pacientes no vienen a buscarla, yo lo sé.

**I:** ¿Y se lo toman?

**PS:** Sí, se lo van a tomar. De hecho, lo suspenden porque no vienen a los controles, entonces se les vence la receta y vuelven después “estuve dos meses comprando el fármaco porque no tenía hora contigo, así que, por favor, hazme la receta”

**PS:** Y el tema de que tomen los medicamentos, también influye mucho la cantidad de medicamentos que toman. Claro, o sea es distinto que tomen dos o tres medicamentos a quienes tienen seis, siete u ocho medicamentos. Entonces, evidentemente, lo van empezando a dejar. Empiezan con gastritis, lo suspenden no más, y a veces ni avisan.

**PS:** Terminando un poco la idea, a mí me pasa que nos dicen, por ejemplo, y me retan, no podí dejar los fármacos un año, porque el paciente no va a venir a los controles. El paciente no va a venir igual. Entonces, ¿qué estamos tratando?, ¿estamos tratando que venga a controles o que se tome los fármacos o estamos haciendo que siga el tratamiento a su pinta?, ¿cuál es la mejor opción, que lo haga como quiere o como nosotros queremos que lo haga él imponiéndole una cuestión? No po.

**I:** Y nuevamente, el estándar y la estructura ………

**PS:** No, y tú… el sistema entero, o sea está envuelto en que la receta se tiene que acabar para que vengan, entonces… chuta, están obligados a venir para que le demos los fármacos y los fármacos son derecho a AUGE. Eeh yo al final, acá uno hace lo que quiere en esta cuestión al final del día y yo les dejo la receta ojalá el mayor tiempo posible, porque sé que, si es así, se lo van a tomar, que no van a tener que comprarlo, abandono por comprar fármacos…

**I:** ¿Y tienes mejores resultados con esos pacientes?

**PS:** Se pierden, como el control es cada tres meses. Entre comillas tres meses porque lo voy a ver en nueve meses más po. Entonces, yo, la verdad, como acordarme de ese caso y ver cómo sigue…

**I:** ustedes pueden igual evaluar por áreas. Tienen pacientes asignados…

**PS:** Sí, masas. Así como el paciente individual que sirvió de estrategia a la otra… no.

**PS:** Estamos limitados por la agenda también. Por ejemplo, nosotros somos una enfermera, en médico y un nutricionista que tenemos que atender a todos a todos los crónicos de un sector. ¿Y cuántos crónicos hay por sector? Como 1.400 crónicos, ¿ya? Eehm entonces eeh, si yo quisiera, por ejemplo, ver a un paciente nuevo que está descompensado y que tengo que revisarle la dieta, como hacerle refuerzo y verlo al mes siguiente. Entonces, ¿de dónde saco horas para verlo? Tengo que derivarlo a médico. Al médico lo puede ver al tiro, lo llamo por teléfono, él hace unos ajustes, pido hora de morbilidad, no hay horas de morbilidad. ¿Horas de crónicos? No, no le corresponden, no hay horas de crónicos hasta tres meses más. Entonces, es como muy complejo llegar a un tratamiento como individual para el paciente.

**I:** ¿qué es lo más complejo de llevar de acuerdo con lo que ustedes perciben?

**PS:** Pastillas de por vida, etiquetado, complicaciones posibles que son catastróficas, eso es lo más pesado, y lo que ven, lo que ven del resto. Es un duelo.

**I:** Y respecto a lo práctico, del día a día, ¿qué es lo más complejo?

**PS:** Acordarse de tomar las pastillas. Saben que están limitados, sí. Saben que están limitados, que tienen que tomarse la pastilla todos los días a las ocho de la mañana, todos los días a las tres de la tarde, todos los días a las cuatro…

**PS:** O sea, yo creo que el tema del fármaco es tomarse las pastillas no más. Cambiar el hábito de vida de salir a caminar, trotar, o sea, ¡esa cuestión no va a pasar! No va a suceder esa cuestión porque…

**PS:** Y asistir, porque tienen que pedir las horas. Tienen que pedir la hora para venir, tienen que pedir la hora para los exámenes…

**I:** Y eso tienen que compatibilizarlo con su vida…

**PS:** Pedir permiso en el trabajo para pacientes cardiovascular…

**I:** ¿Los apoyan del trabajo?

**PS:** Muy poco, muy mal…

**PS:** Hay gente que la despiden por lo mismo, porque tienen que venir mucho a controles. Por ejemplo, los diabéticos tienen que venir cada tres meses a controles. Entonces, no todo el mundo les da la oportunidad de salir y quizás tomarse la mañana…

**PS:** Sobre todo el diabético que inicia insulina que tiene que venir varias veces para acá.

**I:** ¿Y cómo lo hacen en general?

**PS:** No… algunos vienen en pareja como para buscar el apoyo y para ver qué es lo que pueden supervisar.

**PS:** Cuando son adultos mayores, por lo general, viene un hijo o el cuidador y él les pone la medicación, ¿ya? Ahora el tema de qué es lo que más les pesa, qué es lo que más les cuesta, es como, habría que preguntar qué es lo que más le pesa al paciente y lo que más le crea conflicto, ¿ya? Porque el paciente está bien, o sea, el cambio del hábito es difícil, ¿ya? En el adulto mayor digamos que casi casi imposible, pero ¿le crea conflicto el cambio de hábito al paciente o le crea conflicto tomar el medicamento?, ¿te das cuenta?, ¿qué es lo que le crea conflicto? Porque de pronto a él, no sigue la dieta, no hace la actividad física, pero ¿eso le crea conflicto?

**I:** ¿Qué cree usted?

**PS:** Yo pienso que eso no le crea conflicto, o sea, que ellos siguen viviendo su vida, ¿ya? Pero el tema de tener que limitarte y estarte acordando de los medicamentos, yo creo que eso sí porque tienen la ansiedad de que “¡Uy! Hoy día no me tomé la pastilla” o que llegan acá y dicen “¡Uh! No me tomé el medicamento”. Esos están creando el conflicto. No, que te dicen “hoy desayuné un pan con mantequilla y jamón”, “No, es que lo tengo alto porque hoy no tomé el medicamento”

**PS:** Entonces, yo creo que el tema de los medicamentos, el no tomarlos, eso le crea el conflicto y va creando ansiedad, de no haberse acordado, de olvidarse de tomar muchos, ¿ya? Porque eso yo le pregunto, le digo “mire, su presión está alta o su glicemia está aquí”. “¡Ah! No, ¿sabe? Es que hace un mes que no tengo el medicamento” o “Hoy día no me lo tomé, hoy día me lo tomé” o me dicen “¡Qué raro! Porque en la mañana me tomé el medicamento”, ¿te das cuenta? Entonces, ninguno dice “No, es que, en realidad, la semana pasada me desordené un poco”

**PS:** Por eso no quieren venir a los otros controles, porque no entienden pa qué es la educación, pa qué es la nutrición. Es patología, fármaco, y nada más.

**PS:** Por ejemplo, cuando vienen y tienen la hemoglobina glicosilada alterada, yo le digo “Pero está hemoglobina glicosilada está alterada, eso quiere decir que usted hace un tiempito se está portando mal”. “Sí, es que hace un mes tuve un cumpleaños, comí torta…”. Ahí recién asocian el tema de la dieta, ¿te das cuenta? No dicen “¡Ah! Es que no me tomé el medicamento un día”, con la hemoglobina glicosilada, ellos como que toman conciencia cuando uno le dice que se está portando mal y ahí recién dice “Sí, es que la semana pasada hice esto”.

**PS:** Es que es lo más fácil po. Te tomas una pastilla y se acabó el tema. En cambio, lo otro implica esfuerzo real por hacer cosas distintas, plata por cambiar tu alimentación. Entonces eso va más allá de una pastilla. Por eso es por que el tratamiento es súper simple, es farmacológico, una pastilla te la tomai y chao y piensan que se va a acabar todo.

**PS:** Ojalá fuera igual que en el hipotiroidismo, que uno da una pastilla y chao se acabó, pero no es así con las otras patologías.

**PS:** Es un esfuerzo que tienen que adaptar, o sea es un cambio de hábito que tienen que ver de mucho antes. O sea, cuando se le diagnostica diabetes a un paciente, ya es tarde po, ¿ya? Son cambios que deben venir desde mucho antes, incluso desde que son adolescentes. Yo creo que hoy en día, actualmente, como que no se da mucho eso en salud. Como que hay un como un vacío ………a muchas personas, y todos esos cuidados deberían partir desde esa edad, incluso desde que son más niños. Entonces, es complicado cambiar el hábito de una persona de 50, 60 años.

**I:** Muchísimas gracias, estoy feliz. Recabamos un montón de cosas y créanme que nos va a servir muchísimo para poder… Todo eso ustedes lo viven en el día a día. Cuando la gente, o cuando la autoridad, cuando los tomadores de decisiones están un poco alejados de la realidad, de repente hay que mirar la realidad y con esos elementos ir y decir “¡Oye! Esto es lo que está pasando” y vamos a tener que hacernos cargo de esta realidad y eso es lo que en el fondo lo que estamos buscando, poder…

**PS:** Eh lo último, yo creo que también falta un trabajo más que nosotros del CESFAM, a un nivel más eeh no sé, a un nivel con un trabajo con otros centros de salud, la Municipalidad, por ejemplo, colegios, etc., centros de adulto mayor. Falta un trabajo más global.

**I:** Más como de redes.

**PS:** Más como, claro. Generar mejores redes de apoyo a los pacientes porque la diabetes no la ve solamente acá. Está en su casa va a tener diabetes, estando con su grupo de adulto mayor va a tener diabetes. Entonces, eh hay que hacer un trabajo también a nivel con las redes…

**I:** De la comunidad

**PS:** De la comunidad en general. Y a pesar de que, claro esté escrito en el aah cómo se llama éste el, en la guía, en los modelos de uso sanitario se nombran, pero a que se haga al cien por ciento, de buena manera, se trabaje eso.

**I:** ¿cómo la bajada?

**PS:** Claro, yo creo que ahí falta trabajar un poco más y que podría ser una estrategia útil para mantener no sólo al paciente acá, como se decía los controles de treinta minutos no son suficientes. Verlos en sus casas, con sus amigos, si es que trabaja en el trabajo. Yo creo que es difícil, pero hay que ir de a poco no más po.

**I:** Exacto, Muchísimas gracias, nos van a seguir viendo aquí. Porque vamos a seguir con una segunda parte después que va a ser acercarnos un poco a los pacientes eeh y tratar de tener una foto lo más completa posible, pero les agradezco el tiempo. Por favor llévense los sanguchitos incluso para el té de la tarde porque no sé qué puedo hace con todos esos sándwiches. Muchas gracias.
